# Base-Promoted *S_N_Ar* Reactions of Fluoro- and Chloroarenes as a Route to *N*-Aryl Indoles and Carbazoles

**DOI:** 10.3390/molecules24061145

**Published:** 2019-03-22

**Authors:** Muhammad Asif Iqbal, Hina Mehmood, Jiaying Lv, Ruimao Hua

**Affiliations:** Key Laboratory of Organic Optoelectronics & Molecular Engineering of Ministry of Education, Department of Chemistry, Tsinghua University, Beijing 100084, China; ykb15@mails.tsinghua.edu.cn (M.A.I.); hinamehmood123@gmail.com (H.M.); jy-lv18@mails.tsinghua.edu.cn (J.L.)

**Keywords:** base-promoted *S_N_Ar*, haloarene substitution, *N*-arylation of indoles and carbazole

## Abstract

KOH/DMSO-promoted C-N bond formation via nucleophilic aromatic substitution (*S_N_Ar*) between chloroarenes or fluoroarenes with indoles and carbazole under transition metal-free conditions affording the corresponding *N*-arylated indoles and carbazoles has been developed.

## 1. Introduction

Development of efficient methods for the formation of C-N bond via the arylation of N-H bonds is one of the important and perpetual subjects in organic synthetic chemistry. Two major classes of C−N bond formation processes are well-developed: (1) transition-metal-catalyzed *N*-arylation via activation of the C-X bond (X = I, Br, Cl, F) of haloarenes, which have been well-investigated by Hartwig, Buchwald’s [1,2,3], and other groups [4,5,6] and (2) base-promoted nucleophilic aromatic substitution (*S_N_Ar*) reactions of electron-deficient fluoroarenes [7,8,9,10] and bromoarenes [11] with amines. On the other hand, KOH/DMSO has shown versatile diverse activity in a variety of organic transformations developed by Trofimov [12,13,14,15,16,17], Bolm [18,19,20,21] and other groups [22,23,24]. Recently, we have also developed the application of KOH/DMSO in the synthesis of five-membered heterocycles via the cycloaddition of 1,3-butadiynes with H_2_O, primary amines, Na_2_S·9H_2_O [25], and in nucleophilic fluoroarene substitutions with a variety of nucleophiles to provide an alternative base-promoted *S_N_Ar* of C−F bonds [26]. In continuation of our interest in the development of highly atom-economic reactions through C-Cl bond activation in aryl chlorides and their transformation [27,28,29,30,31,32], we have investigated the *N*-arylation of indoles and carbazole by the nucleophilic aromatic substitution (*S_N_Ar*) protocol from chloroarenes and fluoroarenes in the presence of KOH in DMSO. The *N*-arylation of indoles and carbazoles through transition- metal-catalyzed catalysis have been well studied [33,34,35,36,37,38], and a microwave-assisted *N*-arylation of indoles via *S_N_Ar* in the presence of K_2_CO_3_ or Cs_2_CO_3_ under microwave irradiation in DMSO [39], KO*^t^*Bu-promoted *N*-arylations of carbazoles using diaryliodonium salts [40] have also been reported.

## 2. Results and Discussion

The initial investigation was carried out by heating a mixture of 3-methylindole (**1a**) and 1,2-dichlorobenzene (**2a**, 1.0 equiv.) in the presence of KOH (1.0 equiv.) in DMSO at 100 °C under a nitrogen atmosphere for 24 hours. The reaction produced 1-(2-chlorophenyl)-3-methylindole (**3aa**) in 25% isolated yield, accompanied by the formation of 1,2-bis(3-methylindolyl)benzene (**3’aa**, confirmed by MS, yield <5%) as by-product via double *S_N_Ar* of the C−Cl bond of **2a** (entry 1). By doubling the amount of KOH, the yield of **3aa** could be increased up to 55% (entry 2), and the yield of **3aa** could be further improved to 71% by using 2.5 equivalents of **2a** and 3.0 equivalents of KOH (entry 3). Base screening using different inorganic bases such as NaOH, Cs_2_CO_3_ and K_2_CO_3_ in DMSO disclosed that NaOH can also promote the present *S_N_Ar* reaction albeit with relatively low efficiency (entry 4), while Cs_2_CO_3_ and K_2_CO_3_ are ineffective under similar reaction conditions (entries 5,6). On the other hand, when other solvents such as dimethyl acetamide (DMAc), THF, DMF and 1,4-dioxane were used instead of DMSO, no desired product formed at all (entries 7–10).

With the reaction conditions shown in entry 3 of Table 1, the *S_N_Ar* between chloroarenes or fluoroarenes and a variety of indoles were then examined, and the obtained results are listed in Table 2. Among the chloroarenes **2b**~**2i** used, chlorobenzene (**2b**) and 4-chlorotoluene (**2c**) showed relatively low reactivity, while the substitution of 1-chloronaphthalene (**2d**) and 2-chlorothiophene (**2e**) gave the corresponding products **3ad** and **3ae** in good yields. As expected, the chloroarenes bearing electron-withdrawing group(s) undergo the nucleophilic substitution smoothly to give *N*-arylated indoles in good to high yields. It is worth noting that the reaction of **2d** also produced the isomer of 3-methyl-1-(naphthalen-2-yl)indole in trace amounts, and *o*-chlorobenzamide (**2h**), which is an electron-poor chloroarene, shows moderate reactivity, due possibly to its steric hindrance. As expected, when fluoroarenes were employed, the corresponding products could be obtained in good to high yields, owing to the high nucleophilic substitution reactivity exhibited by the C-F bond. In addition, indole (**1b**), 5-substituted indoles **1c** and **1d**, 6-chloroindole (**1e**) and 3-phenylindole (**1f**) can be also used as the nucleophiles, and their nucleophilic substitutions with chloroarenes afforded the corresponding *N*-arylated indoles in fair to good yields.

It can be also concluded from the chemoselective formation of **3am** and **3an** that C-F bonds shows much higher nucleophilic substitution reactivity than C-Cl and C-Br ones. The selective formation of **3ao**, **3ap** and **3aq** indicate that it is difficult for the second *S_N_Ar* reaction of a C-F bond to take place in these products under the reaction conditions. The structure of **3aq** was confirmed by an x-ray diffraction study [41].

In order to evaluate the scope of the present *S_N_Ar*, carbazole was used as nucleophiles under similar reaction conditions, since *N*-arylated carbazoles are important *N*-heterocyclic compounds, which have been widely applied as organic materials [42,43]. As shown in Table 3, when the *S_N_Ar* reactions were performed at 135 °C for 24 h, chloroarenes **2b** and **2c** show low reactivity, and the reactions of electron-poor chloroarenes such as **2f** and **2g** gave the corresponding products **5c** and **5d** in good yields. Fluorobenzene (**2j**) and fluoroarenes having electron-withdrawing groups show good reactivity under the reaction conditions, and the corresponding *N*-arylated carbazoles could be obtained in good yields. However, *p*-fluoroaniline (**2w**) shows a reactivity similar to that of *p*-fluorotoluene (**2r**). In addition, the selective formation of **5g** and **5h** indicates that the second *S_N_Ar* reaction of C-F bond in the products cannot occur under these reaction conditions.

## 3. Materials and Methods 

### 3.1. General Methods

All organic starting materials and solvents were analytically pure and used without further purification. KOH (99.99%) was obtained from Sigma-Aldrich (St. Louis, MO, USA). Nuclear magnetic resonance (NMR) spectra were recorded on ECA-400 or 600 spectrometers (JEOL, Tokyo, Japan) using CDCl_3_ and DMSO-*d*_6_ as a solvent at 298 K. ^1^H-NMR (400 MHz, 600 MHz) chemical shifts (δ) were referenced to internal standard TMS (for ^1^H, δ = 0.00 ppm). ^13^C-NMR (100 MHz, 125 MHz) chemical shifts were referenced to internal solvent (δ = 77.16 ppm in CDCl_3_; 39.52 ppm in DMSO-*d*_6_). Mass spectra (MS) were obtained on a GCMS-QP2010S system (Shimadzu Kyoto, Japan), the high-resolution mass spectra (ESI) were obtained with a micrOTOF-Q 10142 spectrometer (Agilent, California, CA, USA). The melting points are uncorrected.

### 3.2. Typical Experiment Procedure for the Synthesis of 3aa

To a 50 mL screw-capped thick-walled Pyrex tube equipped with a magnetic stirrer, 3-methylindole (**1a**, 131.0 mg, 1.0 mmol), 1,2-dichlorobenzene (**2a**, 365.0 mg, 2.5 mmol), KOH (168.2 mg, 3.0 mmol) and DMSO (5.0 mL) were added sequentially under a nitrogen atmosphere. The tube was then sealed and stirred at 100 °C for 24 h. After removal of the solvent under reduced pressure, purification was performed by flash column chromatography on silica gel with petroleum ether/ethyl acetate (gradient mixture ratio from 100:0 to 90:10) as eluent to afford *N*-(2-chlorophenyl)-3-methylindole (3aa, 171.8 mg, 0.71 mmol, 71% yield).

### 3.3. Typical Experiment Procedure for the Synthesis of ***5a***

To a 50 mL screw-capped thick-walled Pyrex tube equipped with a magnetic stirrer, carbazole (**4a**, 167.2 mg, 1.0 mmol), chlorobenzene (**2b**, 281.4 mg, 2.5 mmol), KOH (168.2 mg, 3.0 mmol) and DMSO (5.0 mL) were added sequentially under nitrogen atmosphere. The tube was then sealed and stirred at 135 °C in an oil bath for 48 h. After removal of the solvent under reduced pressure, purification was performed by flash column chromatography on silica gel with petroleum ether/ethyl acetate (gradient mixture ratio from 100:0 to 85:15) as eluent to afford *N*-phenylcarbazole (**5a**, 77.8 mg, 0.32 mmol, 32% yield).

### 3.4. Characterization Data of Products

*N-(2-Chlorophenyl)-3-methylindole* (**3aa**): White waxy oil (171.8 mg, 71%); ^1^H-NMR (400 MHz, CDCl_3_) δ 7.66 (d, *J* = 6.8 Hz, 1H), 7.60 (d, *J* = 9.4 Hz, 1H), 7.44–7.35 (m, 3H), 7.26–7.12 (m, 3H), 7.06 (s, 1H), 2.43 (s, 3H); ^13^C-NMR (100 MHz, CDCl_3_) δ 137.2, 137.1, 131.7, 130.9, 129.4, 129.1, 128.7, 127.7, 126.3, 122.3, 119.8, 119.1, 112.6, 110.67, 9.7; HRMS (ESI): *m*/*z* Calcd. For: C_15_H_12_ClN [M + H]^+^: 242.0731; found 242.0721.

*3-Methyl-N-phenylindole* (**3ab**) [44]: White waxy oil (from **2b**, 64.2 mg, 31%; from **2j**, 140.9 mg, 68%); ^1^H-NMR (400 MHz, CDCl_3_) δ 7.64 (d, *J* = 7.6 Hz, 1H), 7.58 (d, *J* = 8.0 Hz, 1H), 7.54–7.47 (m, 4H), 7.35–7.31 (m, 1H), 7.24–7.16 (m, 2H), 7.16 (s, 1H), 2.40 (s, 3H); ^13^C-NMR (100 MHz, CDCl_3_) δ 140.1, 136.0, 129.9, 129.6, 126.0, 125.6, 124.1, 122.4, 119.8, 119.3, 112.9, 110.5, 9.7; GC-MS *m*/*z*: 207 (M^+^).

*3-Methyl-N-(p-tolyl)indole* (**3ac**) [38]: White waxy oil (53.1 mg, 24%); ^1^H-NMR (400 MHz, CDCl_3_) δ 7.66 (d, *J* = 7.5 Hz, 1H), 7.55 (d, *J* = 8.0 Hz, 1H), 7.39 (d, *J* = 8.3 Hz, 2H), 7.31 (d, *J* = 8.3 Hz, 2H), 7.26–7.16 (m, 2H), 7.14 (s, 1H), 2.45 (s, 3H), 2.42 (s, 3H); ^13^C-NMR (100 MHz, CDCl_3_) δ 137.6, 136.2, 135.9, 130.2, 129.7, 125.7, 124.0, 122.3, 119.7, 119.2, 112.5, 110.5, 21.1, 9.7; GC-MS *m*/*z*: 221 (M+).

*3-Methyl-N-(naphthalen-1-yl)indole* (**3ad**): White waxy oil (146.6 mg, 57%); ^1^H-NMR (400 MHz, CDCl_3_) δ 7.96 (t, *J* = 8.7 Hz, 2H), 7.70 (d, *J* = 7.7 Hz, 1H), 7.61–7.50 (m, 4H), 7.43–7.39 (m, 1H), 7.21–7.12 (m, 3H), 7.03 (d, *J* = 8.1 Hz, 1H), 2.47 (s, 3H); ^13^C-NMR (100 MHz, CDCl_3_) δ 138.3, 136.4, 134.6, 130.6, 129.0, 128.3, 128.2, 127.5, 126.9, 126.6, 125.6, 125.1, 123.7, 122.2, 119.5, 119.1, 112.2, 110.8, 9.8; HRMS (ESI): *m*/*z* Calcd. For: C_19_H_15_N [M + H]^+^: 258.1277; found 258.1275.

*3-Methyl-1-(thiophen-2-yl)indole* (**3ae**): White waxy oil (138.4 mg, 65%,); ^1^H-NMR (600 MHz, CDCl_3_) δ 7.60 (dd, *J* = 13.2, 8.0 Hz, 2H), 7.30–7.25 (m, 1H), 7.23–7.19 (t, *J* = 7.4 Hz, 1H), 7.16–7.13 (t, *J* = 4.0 Hz, 1H), 7.10 (s, 1H), 7.05 (d, *J* = 3.1 Hz, 2H), 2.37 (s, 3H); ^13^C-NMR (125 MHz, CDCl_3_) δ 142.2, 137.2, 129.7, 126.8, 126.1, 122.9, 121.0, 120.4, 119.5, 119.2, 113.6, 110.6, 9.6; HRMS (ESI): *m*/*z* Calcd. For: C_13_H_11_NS [M + H]^+^: 214.0685; found 214.0681.

*3-Methyl-N-(4-nitrophenyl)indole* (**3af**) [45]: Yellow solid (176.4 mg, 70%); ^1^H-NMR (400 MHz, CDCl_3_) δ 8.36 (d, *J* = 8.9 Hz, 2H), 7.68–7.57 (m, 4H), 7.33–7.20 (m, 2H), 7.17 (s, 1H), 2.38 (s, 3H); ^13^C-NMR (100 MHz, CDCl_3_) δ 145.5, 144.6, 135.5, 130.9, 125.6, 124.5, 123.6, 122.7, 121.2, 119.8, 115.8, 110.5, 9.7; GC-MS *m*/*z*: 252 (M^+^).

*3-Methyl-N-(pyrimidin-2-yl)indole* (**3ag**) [46]: White solid (165.3 mg, 79 %); ^1^H-NMR (400 MHz, CDCl_3_) δ 8.77 (d, *J* = 9.0 Hz, 1H), 8.66 (d, *J* = 4.8 Hz, 2H), 8.03 (s, 1H), 7.56 (d, *J* = 8.4 Hz, 1H), 7.42–7.19 (m, 2H), 6.98 (t, *J* = 4.8 Hz, 1H), 2.35 (s, 3H); ^13^C-NMR (100 MHz, CDCl_3_) δ 158.0, 157.7, 135.7, 132.1, 123.7, 122.9, 121.8, 118.8, 116.3, 116.0, 115.5, 9.8; GC-MS *m*/*z*: 209 (M^+^).

*2-(3-Methyl-indol-1-yl)benzamide* (**3ah**) [47]: White waxy oil (140.1 mg, 56%); ^1^H-NMR (400 MHz, DMSO-d6) δ 7.67–7.53 (m, 4H), 7.50–7.41 (m, 2H), 7.32 (s, 1H), 7.19–7.05 (m, 4H), 2.29 (s, 3H); ^13^C-NMR (100 MHz, DMSO-*d*_6_) δ 168.8, 165.0, 136.5, 135.9, 134.8, 130.4, 128.8, 127.3, 127.1, 126.9, 121.8, 119.3, 118.6, 111.0, 110.2. 9.5; GC-MS *m*/*z*: 250 (M^+^).

*N-(2-Chloro-4-nitrophenyl)-3-methylindole* (**3ai**): Orange solid (249.4 mg, 87%); mp 125~130 °C; ^1^H-NMR (600 MHz, CDCl_3_) δ 8.49 (s, 1H), 8.24 (d, *J* = 8.7 Hz, 1H), 7.65 (d, *J* = 7.3 Hz, 1H), 7.62 (d, *J* = 8.7 Hz, 1H), 7.27–7.19 (m, 4H), 7.11 (s, 1H), 2.41 (s, 3H); ^13^C-NMR (125 MHz, CDCl_3_) δ 146.2, 142.8, 136.4, 131.3, 129.8, 128.8, 126.8, 125.5, 123.1, 122.9, 120.9, 119.6, 114.6, 110.6, 9.7; HRMS (ESI): *m*/*z* Calcd. For: C_15_H_11_ClNO_2_ [M + H]^+^: 287.0582; found 287.0576.

*N-(2-Bromophenyl)-3-methylindole* (**3am**) [48]: White waxy oil (214.6 mg, 75%); ^1^H-NMR (400 MHz, CDCl_3_) δ 7.80 (d, *J* = 7.9 Hz, 1H), 7.70–7.64 (m, 1H), 7.50–7.40 (m, 2H), 7.37–7.29 (m, 1H), 7.28–7.19 (m, 2H), 7.17–7.10 (m, 1H), 7.07 (s, 1H), 2.46 (s, 3H); ^13^C-NMR (100 MHz, CDCl_3_) δ 138.8, 137.1, 134.0, 129.8, 129.2, 129.0, 129.3, 122.3, 121.8, 119.7, 119.1, 112.5, 110.6, 9.8; GC-MS *m*/*z*: 287 (M^+^).

*N-(3-Bromo-5-chlorophenyl)-3-methylindole* (**3an**): White solid (221.2 mg, 69 %); mp 142~144 °C; ^1^H-NMR (400 MHz, CDCl_3_) δ 7.62 (d, *J* = 8.4 Hz, 1H), 7.57–7.55 (m, 2H), 7.45–7.44 (m, 2H), 7.29–7.19 (m, 2H), 7.09 (s, 1H), 2.37 (s, 3H); ^13^C-NMR (100 MHz, CDCl3) δ 141.9, 136.0, 135.6, 130.3, 128.4, 124.8, 124.7, 123.4, 123.2, 122.4, 120.7, 119.6, 114.5, 110.2, 9.6; HRMS (ESI): *m*/*z* Calcd. For: C_15_H_11_BrClN [M + H]^+^: 319.9836; found 319.9834.

*N-(2-Fluoro-3-propylphenyl)-3-methylindole* (**3ao**): White waxy oil (216.5 mg, 81%); ^1^H-NMR (400 MHz, CDCl_3_) δ 7.65 (d, *J* = 7.4 Hz, 1H), 7.34–7.30 (m, 2H), 7.24–7.15 (m, 4H), 7.10 (s, 1H), 2.74 (t, *J* = 7.6 Hz, 2H), 2.42 (s, 3H), 1.77–1.68 (m, 2H), 1.03 (t, *J* = 7.3 Hz, 3H); ^13^C-NMR (100 MHz, CDCl_3_) δ 155.2 (d, *J*_C-F_ = 248.0 Hz), 136.8, 131.4 (d, *J*_C-F_ = 15.0 Hz), 129.4, 128.9 (d, *J*_C-F_ = 5.0 Hz), 127.4 (d, *J*_C-F_ = 13.0 Hz), 126.3 (d, *J*_C-F_ = 2.0 Hz), 125.2 (d, *J*_C-F_ = 1.0 Hz), 124.1 (d, *J*_C-F_ = 5.0 Hz), 122.4, 119.8, 119.2, 112.8, 110.7, 31.3, 23.5, 13.9, 9.7; HRMS (ESI): *m*/*z* Calcd. For: C_18_H_18_FN_2_ [M + H]^+^: 268.1496; found 268.1492.

*N-(3-Fluorophenyl)-3-methylindole* (**3ap**): White waxy oil (164.7 mg, 73%); ^1^H-NMR (400 MHz, CDCl_3_) δ 7.66–7.60 (m, 2H), 7.49–7.43 (m, 1H), 7.31–7.20 (m, 4H), 7.14 (s, 1H), 7.05–7.01 (m, 1H), 2.41 (s, 3H); ^13^C-NMR (100 MHz, CDCl_3_) δ 164.4 (d, *J*_C-F_ = 246.0 Hz), 141.6 (d, *J*_C-F_ = 10.0 Hz), 135.8, 130.9 (d, *J*_C-F_ = 10.0 Hz), 130.1, 125.2, 122.8, 120.3, 119.4 (d, *J*_C-F_ = 13.0 Hz), 113.7, 112.7 (d, *J*_C-F_ = 21.0 Hz), 111.2, 111.0, 110.4, 9.7; HRMS (ESI): *m*/*z* Calcd. For: C_15_H_12_FN [M + H]^+^: 226.1027; found 226.1025.

*2-Fluoro-6-(3-methylindol-1-yl)benzamide* (**3aq**): White solid (203.9 mg, 76%); mp 205~208 **°**C; ^1^H-NMR (400 MHz, CDCl_3_) δ 7.61 (d, *J* = 6.7 Hz, 1H), 7.55–7.47 (m, 1H), 7.34–7.24 (m, 2H), 7.23–7.15 (m, 3H), 7.09 (s, 1H), 5.43 (d, *J* = 63.2 Hz, 2H), 2.35 (s, 3H); ^13^C-NMR (100 MHz, CDCl_3_) δ 164.8, 160.2 (d, *J*_C-F_ = 251.0 Hz), 138.3, 136.9, 131.5 (d, *J*_C-F_ = 10.0 Hz), 129.6, 126.3, 123.2, 122.8, 120.3, 119.4, 115.0, 114.8, 113.7, 110.1, 9.7; HRMS (ESI): *m*/*z* Calcd. For: C_16_H_13_FN_2_O [M + H]^+^: 269.1085; found 269.1082.

*N-(2-Chlorophenyl)indole* (**3ba**) [49]: White waxy oil (111.5 mg, 49%); ^1^H-NMR (400 MHz, CDCl_3_) δ 7.70 (d, *J* = 6.5 Hz, 1H), 7.61–7.58 (m, 1H), 7.47–7.39 (m, 3H), 7.26–7.13 (m, 4H), 6.71 (d, *J* = 3.3 Hz, 1H); ^13^C-NMR (100 MHz, CDCl_3_) δ 137.0, 136.8, 131.9, 130.9, 129.5, 129.1, 128.8, 128.6, 127.7, 122.4, 121.1, 120.4, 110.7, 103.3; GC-MS *m*/*z*: 227 (M^+^).

*N-Phenylindole* (**3bb**) [50]: White solid (from **2j**, 98.5 mg, 51%); ^1^H-NMR (400 MHz, CDCl_3_) δ 7.70 (d, *J* = 8.3 Hz, 1H), 7.58 (d, *J* = 8.2 Hz, 1H), 7.53–7.51 (m, 4H), 7.39–7.33 (m, 2H), 7.25–7.15 (m, 2H), 6.70 (d, *J* = 3.3 Hz,1H); ^13^C-NMR (100 MHz, CDCl_3_) δ 139.9, 135.9, 129.7, 129.4, 128.0, 126.5, 124.5, 122.4, 121.2, 120.4, 110.6, 103.6; GC-MS *m*/*z*: 193 (M^+^). 

*N-(2-Chlorophenyl)indole-5-carbonitrile* (**3ca**): White solid (75.6 mg, 30%); mp 50~54 **°**C; ^1^H-NMR (400 MHz, CDCl_3_) δ 8.04 (s, 1H), 7.63–7.61 (m, 1H), 7.47–7.41 (m, 4H), 7.35 (d, *J* = 3.3 Hz, 1H), 7.15 (d, *J* = 8.6 Hz, 1H), 6.78 (d, *J* = 3.9 Hz, 1H); ^13^C-NMR (100 MHz, CDCl_3_) δ 138.4, 135.9, 132.0, 131.2, 131.1, 130.1, 129.4, 128.3, 128.0, 126.7, 125.4, 120.6, 111.6, 104.1, 103.7; HRMS (ESI): *m*/*z* Calcd. For: C_15_H_9_ClN_2_ [M + H]^+^: 270.0793; found 270.0791.

*N-(2-Chlorophenyl)-indole-5-carboxamide* (**3da**): White solid (157.0 mg, 58%); mp 130~134 °C; ^1^H-NMR (400 MHz, CDCl_3_) δ 8.21 (s, 1H), 7.69 (d, *J* = 10.4 Hz, 1H), 7.62–7.60 (m, 1H), 7.45–7.42 (m, 3H), 7.31 (d, *J* = 3.3 Hz, 1H), 7.14 (d, *J* = 8.6 Hz, 1H), 6.77 (d, *J* = 4.0 Hz, 1H), 6.02 (s_br_, 2H); ^13^C-NMR (100 MHz, CDCl_3_) δ 170.5, 138.7, 136.4, 131.9, 131.0, 130.4, 129.7, 129.4, 128.2, 127.9, 125.7, 121.9, 121.3, 110.7, 104.4; HRMS (ESI): *m*/*z* Calcd. For: C_15_H_11_ClN_2_O [M + H]^+^: 271.0633; found 271.0630.

*6-Chloro-N-phenylindole* (**3eb**): Pale yellow waxy oil (59.1 mg, 26%); ^1^H-NMR (400 MHz, CDCl_3_) δ 7.62–7.45 (m, 6H), 7.39 (t, *J* = 7.2 Hz, 1H), 7.33 (d, *J* = 3.3 Hz, 1H), 7.14 (d, *J* = 8.4 Hz, 1H), 6.66 (d, *J* = 3.0 Hz, 1H); ^13^C-NMR (100 MHz, CDCl_3_) δ 139.3, 136.4, 129.9, 128.8, 128.5, 127.8, 127.0, 124.5, 122.0, 121.1, 110.6, 103.7; HRMS (ESI): *m*/*z* Calcd. For: C_14_H_10_ClN [M + H]^+^: 228.0575; found 228.0574.

*1,3-Diphenylindole* (**3fb**) [51]: White solid (91.5 mg, 34%); ^1^H-NMR (400 MHz, CDCl_3_) δ 8.01 (d, *J* = 7.3 Hz, 1H), 7.74 (d, *J* = 7.3 Hz, 2H), 7.62 (d, *J* = 7.4 Hz, 1H), 7.57–7.46 (m, 7H), 7.42–7.24 (m, 4H); ^13^C-NMR (100 MHz, CDCl_3_) δ 139.6, 136.8, 135.2, 129.8, 128.9, 127.7, 127.2, 126.8, 126.3, 125.6, 124.6, 122.9, 121.0, 120.2, 119.2, 110.9; GC-MS *m*/*z*: 269 (M^+^).

*N-Phenylcarbazole* (**5a**) [37]: White solid (from **2j**, 121.6 mg, 70%); ^1^H-NMR (400 MHz, CDCl_3_) δ 8.15 (d, *J* = 7.7 Hz, 2H), 7.65–7.54 (m, 4H), 7.47 (t, *J* = 7.1 Hz, 1H), 7.41 (d, *J* = 4.0 Hz, 4H), 7.33–7.27 (m, 2H); ^13^C-NMR (100 MHz, CDCl_3_) δ 141.0, 137.8, 129.9, 127.5, 127.2, 126.0, 123.4, 120.4, 120.0, 109.8; GC-MS *m*/*z*: 243 (M^+^).

*N-(p-Tolyl)carbazole* (**5b**) [37]: White solid (from **2r**, 67.4 mg, 30%,); ^1^H-NMR (400 MHz, CDCl_3_) δ 8.15 (d, *J* = 8.3 Hz, 2H), 7.50–7.36 (m, 8H), 7.29 (t, *J* = 6.9 Hz, 12), 2.49 (s, 3H),^13^C-NMR (100 MHz, CDCl_3_) δ 141.1, 137.5, 135.1, 130.6, 127.1, 125.9, 123.3, 120.3, 119.8, 109.9, 21.3, GC-MS *m*/*z*: 257 (M^+^).

*N-(4-Nitrophenyl)carbazole* (**5c**) [52]: Yellow solid (from **2k**, 206.6 mg, 70%); ^1^H-NMR (400 MHz, CDCl_3_) δ 8.49 (d, *J* = 8.8 Hz, 2H), 8.16 (d, *J* = 7.7 Hz, 2H), 7.81 (d, *J* = 8.8 Hz, 2H), 7.48 (m, 4H), 7.36 (t, *J* = 7.4 Hz, 2H); ^13^C-NMR (100 MHz, CDCl_3_) δ 145.9, 143.9, 139.9, 126.8, 126.6, 125.6, 124.3, 121.3, 120.7, 109.7; GC-MS *m*/*z*: 288 (M^+^).

*N-(Pyrimidin-2-yl)carbazole* (**5d**) [53]: White solid (156.9 mg, 64%); ^1^H-NMR (400 MHz, CDCl_3_) δ 8.87 (d, *J* = 8.5 Hz, 2H), 8.83 (d, *J* = 4.8 Hz, 2H), 8.09 (d, *J* = 7.7 Hz, 2H), 7.53 (t, *J* = 7.8 Hz, 2H), 7.39 (t, *J* = 7.5 Hz, 2H), 7.10 (t, *J* = 4.8 Hz, 1H); ^13^C-NMR (100 MHz, CDCl_3_) δ 157.9, 139.2, 126.7, 125.9, 122.4, 119.6, 116.3, 116.1; GC-MS *m*/*z*: 245 (M^+^).

*N-(3-Fluorophenyl)carbazole* (**5e**) [37]: White solid (177.6 mg, 68%); ^1^H-NMR (400 MHz, CDCl_3_) δ 8.11 (d, *J* = 7.7 Hz, 2H), 7.57–7.47 (m, 1H), 7.46–7.24 (m, 8H), 7.21–7.08 (m, 1H); ^13^C-NMR (100 MHz, CDCl_3_) δ 163.5 (d, *J*_C-F_ = 246.0 Hz), 140.6, 139.4 (d, *J*_C-F_ = 10.0 Hz), 131.2 (d, *J*_C-F_ = 9.0 Hz), 126.2, 123.6, 122.4 (d, *J*_C-F_ = 6.0 Hz), 120.5, 120.4, 114.6 (d, *J*_C-F_ = 7.0 Hz), 114.4 (d, *J*_C-F_ = 9.0 Hz), 109.8; GC-MS *m*/*z*: 261 (M^+^).

*N-(2-Fluorophenyl)carbazole* (**5f**) [37]: White waxy oil (148.9 mg, 57%); ^1^H-NMR (400 MHz, CDCl_3_) δ 8.25 (d, *J* = 8.2 Hz, 2H), 7.72 (t, *J* = 7.8 Hz, 1H), 7.68–7.57 (m, 2H), 7.50 (t, *J* = 7.4 Hz, 1H), 7.43 (t, *J* = 7.7 Hz, 2H), 7.30 (t, *J* = 7.4 Hz, 2H), 7.19 (d, *J* = 8.2 Hz, 2H); ^13^C-NMR (100 MHz, CDCl_3_) δ 157.6 (d, *J*_C-F_ = 248.0 Hz), 140.2, 130.5 (d, *J*_C-F_ = 7.0 Hz), 129.9, 126.4, 125.9 (d, *J*_C-F_ = 3.0 Hz), 124.0 (d, *J*_C-F_ = 13.0 Hz), 122.8, 120.5, 120.3, 117.4 (d, *J*_C-F_ = 19.0 Hz), 109.6; HRMS (ESI): *m*/*z* Calcd. For: C_18_H_12_FN [M + H]^+^: 262.1027; found 262.1025.

*N-(2-Fluoro-3-propylphenyl)carbazole* (**5g**): White waxy oil (151.5 mg, 50%); ^1^H-NMR (400 MHz, DMSO-*d*_6_) δ 8.24 (d, *J* = 7.7 Hz, 2H), 7.55–7.35 (m, 5H), 7.29 (t, *J* = 7.5 Hz, 2H), 7.17 (d, *J* = 8.2 Hz, 2H), 2.70 (t, *J* = 7.5 Hz, 2H), 1.68–1.63 (m, 2H), 0.94 (t, *J* = 7.4 Hz, 3H); ^13^C-NMR (100 MHz, CDCl_3_) δ 155.9 (d, *J*_C-F_ = 247.0 Hz), 140.2, 131.0 (d, *J*_C-F_ = 16.0 Hz), 130.8 (d, *J*_C-F_ = 5.0 Hz), 127.2, 126.3, 125.1 (d, *J*_C-F_ = 4.0 Hz), 123.9 (d, *J*_C-F_ = 20.0 Hz), 122.7, 120.5, 120.2, 109.6, 30.2, 22.9, 13.5; HRMS (ESI): *m*/*z* Calcd. For: C_21_H_18_FN [M + H]^+^: 304.1496; found 304.1491.

*N-(2-Fluoro-4-nitrophenyl)-9H-carbazole* (**5h**): Orange solid (198.9 mg, 65%); mp 80~85 **°**C; ^1^H-NMR (400 MHz, CDCl_3_) δ 8.33–8.23 (m, 2H), 8.14 (d, *J* = 7.7 Hz, 2H), 7.84 (t, *J* = 7.7 Hz, 1H), 7.45 (t, *J* = 7.7 Hz, 2H), 7.35 (t, *J* = 7.5 Hz, 2H), 7.26 (d, *J* = 6.0 Hz, 2H); ^13^C-NMR (100 MHz, CDCl_3_) δ 157.2 (d, *J*_C-F_ = 251.0 Hz), 147.2 (d, *J*_C-F_ = 8.0 Hz), 140.0, 131.9 (d, *J*_C-F_ = 12.0 Hz), 129.7 (d, *J*_C-F_ = 2.0 Hz), 126.6, 124.3, 121.4, 120.7, 120.6 (d, *J*_C-F_ = 4.0 Hz), 113.8 (d, *J*_C-F_ = 25.0 Hz), 110.0 (d, *J*_C-F_ = 9.0 Hz); HRMS (ESI): *m*/*z* Calcd. For: C_18_H_11_FN_2_O_2_ [M − H]^−^: 305.0732; found 305.0731.

*4-(Carbazol-9-yl)benzamide* (**5i**) [52]: White solid (168.9 mg, 59%); ^1^H-NMR (400 MHz, CDCl_3_) δ 8.15 (d, *J* = 7.7 Hz, 2H), 8.07 (d, *J* = 8.3 Hz, 2H), 7.69 (d, *J* = 8.1 Hz, 2H), 7.45–7.38 (m, 4H), 7.32 (t, *J* = 7.5 Hz, 2H), 6.14 (s_br_, 2H); ^13^C-NMR (100 MHz, CDCl_3_) δ 168.6, 141.3, 140.4, 131.9, 129.3, 126.8, 126.3, 123.8, 120.6, 120.5, 109.8; GC-MS *m*/*z*: 286 (M^+^).

*4-(Carbazol-9-yl)aniline* (**5j**) [52]: Pale yellow waxy oil (77.5 mg, 30%); ^1^H-NMR (400 MHz, DMSO-*d*_6_) δ 8.20 (d, *J* = 7.7 Hz, 2H), 7.39 (t, *J* = 7.6 Hz, 2H), 7.32–7.15 (m, 6H), 6.80 (d, *J* = 8.6 Hz, 2H), 5.45 (s, 2H); ^13^C-NMR (100 MHz, CDCl_3_) δ 148.5, 140.9, 127.7, 125.9, 124.4, 122.1, 120.3, 119.3, 114.6, 109.6; GC-MS *m*/*z*: 258 (M^+^).

The charts of ^1^H- and ^13^C-NMR are available in Supplementary Materials.

## 4. Conclusions 

In summary, we have investigated the *S_N_Ar* reactions of chloroarenes and fluoroarenes to achieve the *N*-arylation of indoles and carbazole with the use of KOH/DMSO as a medium under transition-metal-free conditions, providing an alternative and efficient protocol for the synthesis of *N*-arylated indoles and carbazoles. The present procedure has the significant advantage of tolerance to various functional groups, which are important for further synthesis of indole- and carbazole-based organic materials.

## Figures and Tables

**Table 1:**
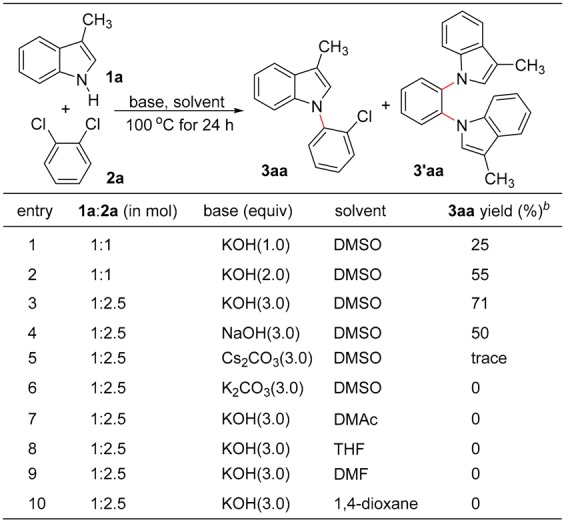
Optimizing conditions for the nucleophilic aromatic substitution of 3-methylindole (**1a**) with 1,2-dichlorobenzene (**2a**) *^a^*.

*^a^* Reactions were carried out using 1.0 mmol of **1a** in 5.0 mL of DMSO at 100 °C for 24 h. *^b^* The yields are isolated yields.

**Table 2:**
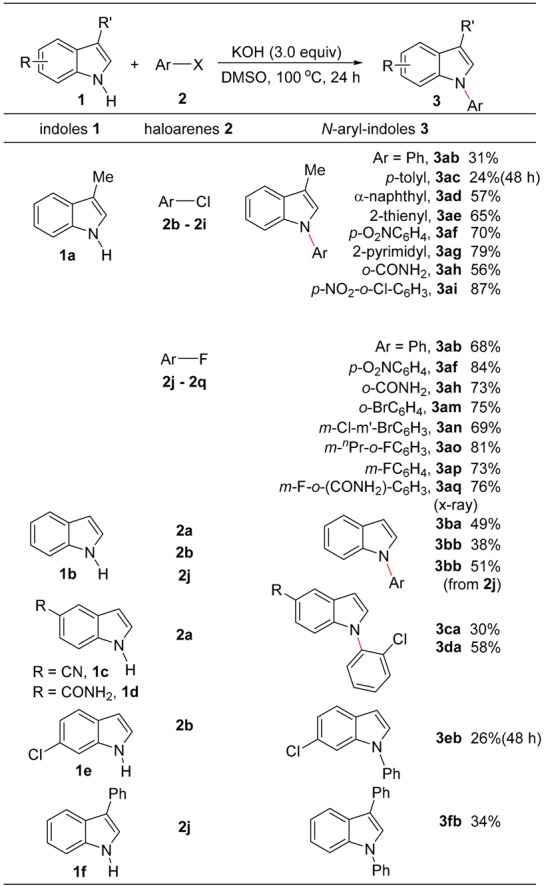
Substrate scope for *N*-arylation of indoles with chloro- and fluoroarenes *^a^*.

*^a^ Reaction conditions*: indoles (1.0 mmol), aryl halide (2.5 mmol), KOH (3.0 mmol), DMSO (5 mL), 100 °C for 24 h; isolated yields for all products.

**Table 3:**
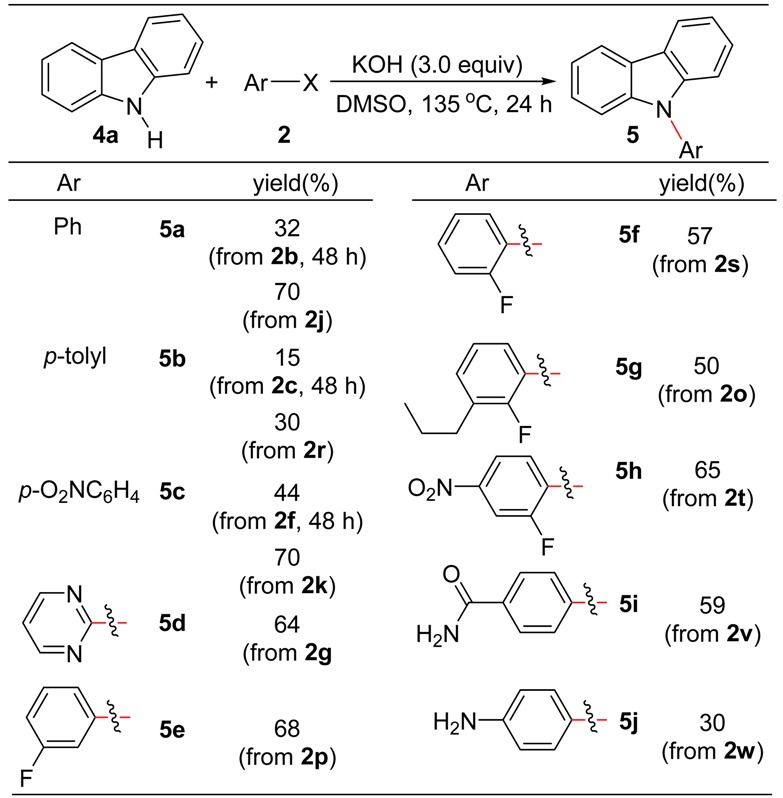
*N*-Arylation of carbazole with chloro- and fluoroarenes *^a^*.

*^a^ Reaction conditions*: carbazole (1.0 mmol), aryl halide (2.5 mmol), KOH (3.0 mmol), DMSO (5 mL), 135 °C for 24 h in N_2_; isolated yields for all products.

## Supplementary Materials

The ^1^H- and ^13^C-NMR spectra of the products are available online.
