# Supplementary material for: Base-Promoted SNAr Reactions of Fluoro- and Chloroarenes as a Route to N-Aryl Indoles and Carbazoles
Source: Molecules. 2019 Mar 22;24(6):1145. doi: 10.3390/molecules24061145 (PMC6470585; doi:10.3390/molecules24061145)
Supplement: Supplementary file 1 [file molecules-24-01145-s001.pdf]

Supplementary File

Base-promoted S_NAr reactions of fluoro- and chloroarenes as an access to *N*-aryl indoles and carbazoles

Muhammad Asif Iqbal, HinaMehmood, Jiaying Lv, Ruimao Hua*

Department of Chemistry, Tsinghua University, Key Laboratory of Organic Optoelectronics & Molecular Engineering of Ministry of Education, Beijing 100084, China

E-mail: ruimao@mail.tsinghua.edu.cn

1. **The ^{1}H- and ^{13}C-NMR charts of all the products** ……………………………… S1-S31
2. **The data of 3aq crystal Structure** ……………………………………………….. S32-S45

1. The ^{1}H- and ^{13}C-NMR charts of all the products

^{1}H-NMR spectrum of **3aa** (400 MHz, $CDCl_3$)

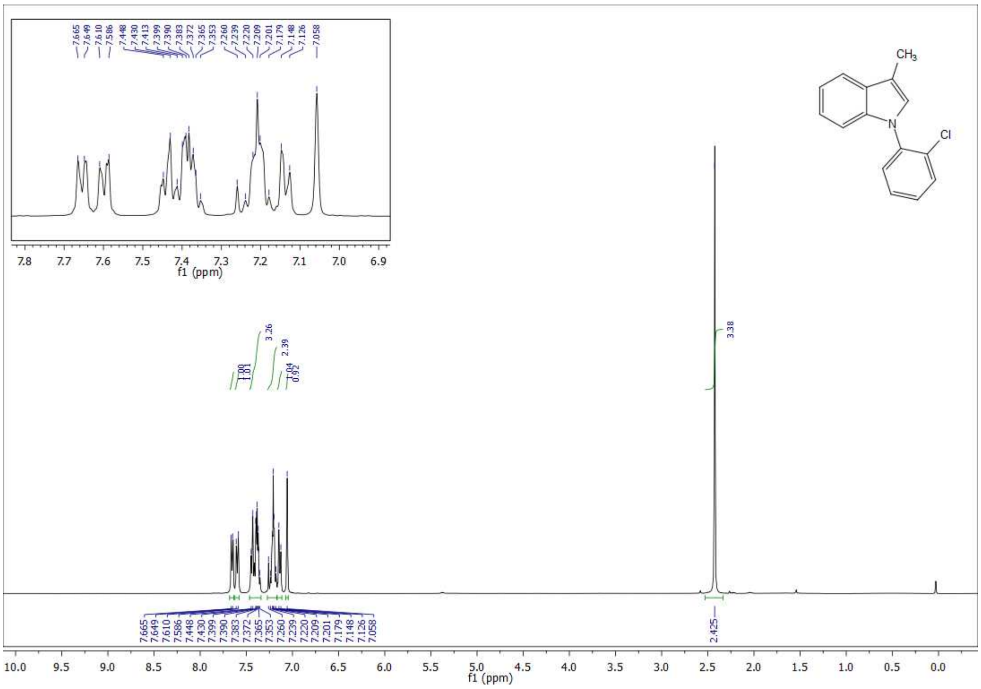

^{13}C-NMR spectrum of **3aa** (100 MHz, $CDCl_3$)

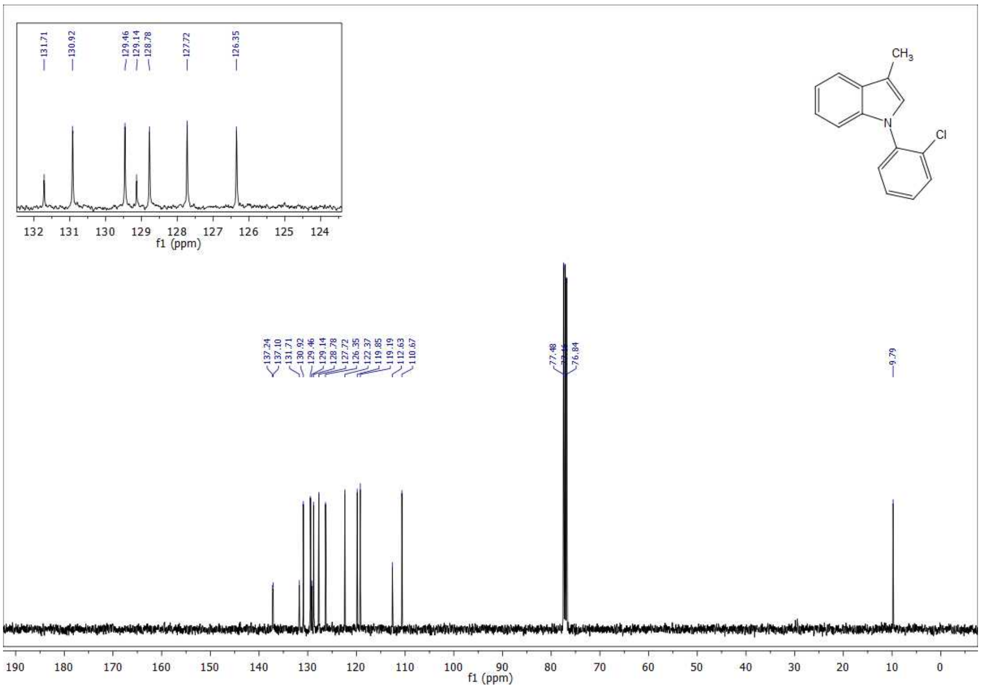

^{1}H-NMR spectrum of **3ab** (400 MHz, $CDCl_3$)

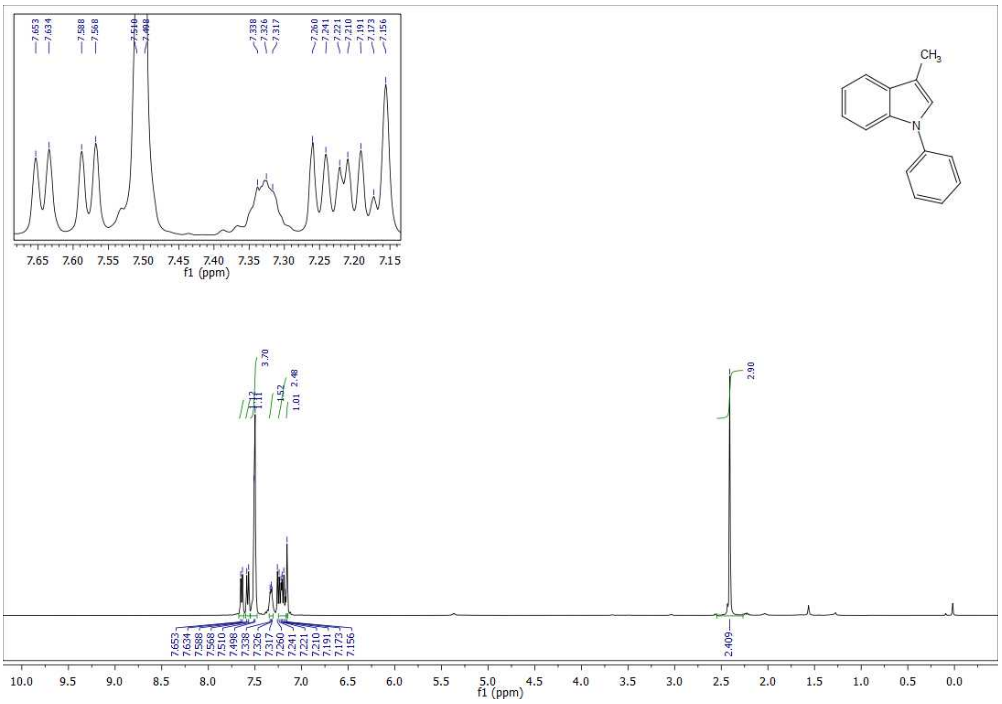

^{13}C-NMR spectrum of **3ab** (100 MHz, $CDCl_3$)

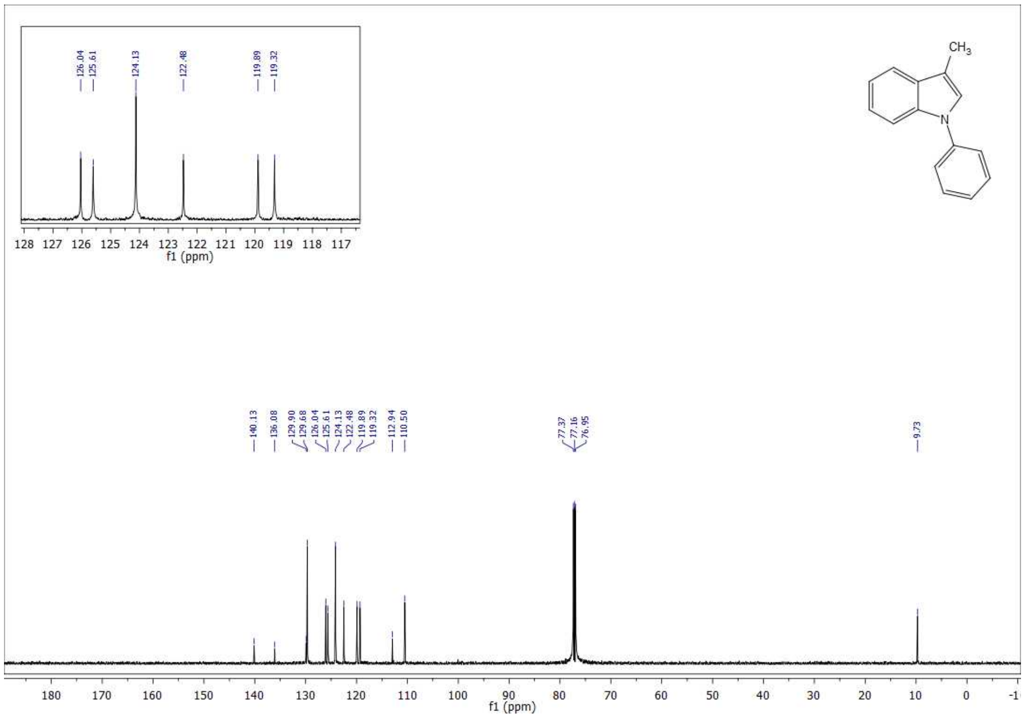

^{1}H-NMR spectrum of **3ac** (400 MHz, $CDCl_3$)

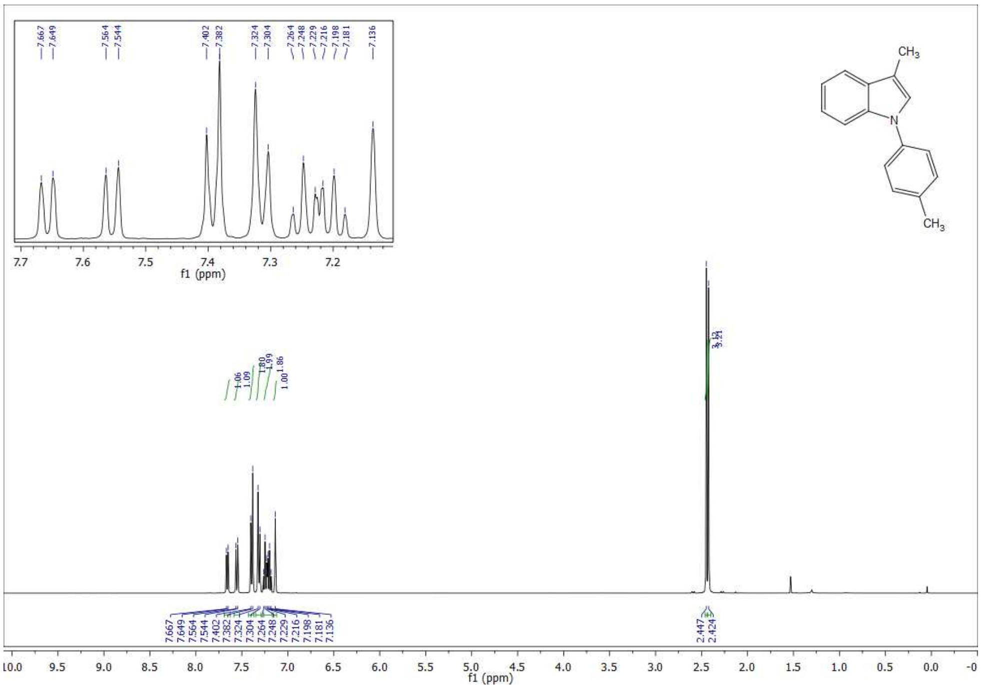

^{13}C-NMR spectrum of **3ac** (100 MHz, $CDCl_3$)

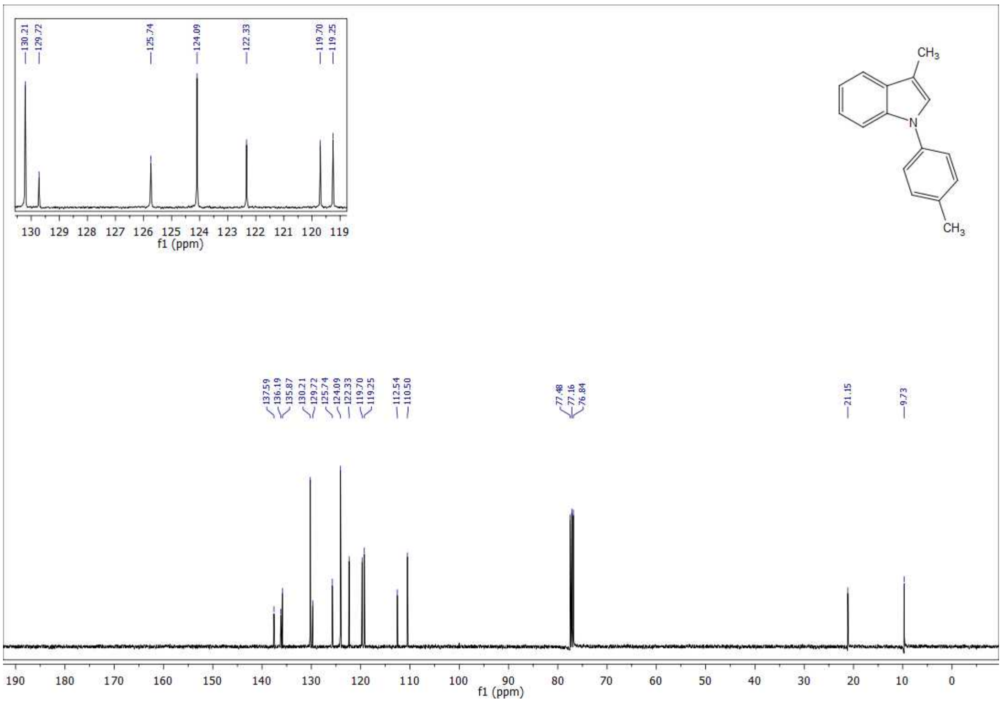

^{1}H-NMR spectrum of **3ad** (400 MHz, $CDCl_3$)

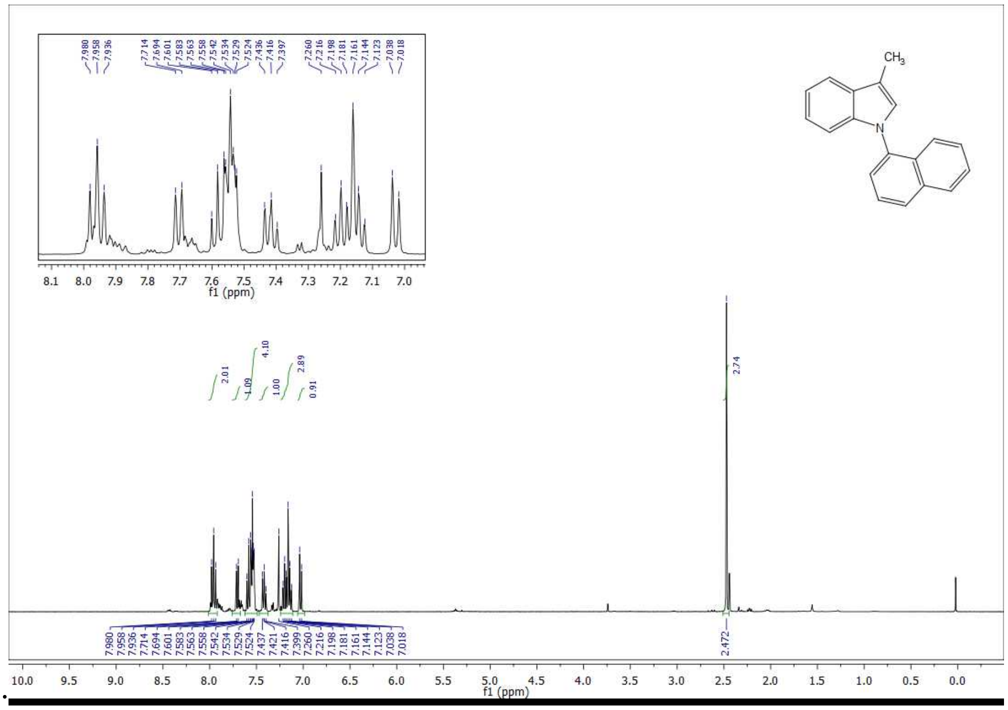

^{13}C-NMR spectrum of **3ad** (100 MHz, $CDCl_3$)

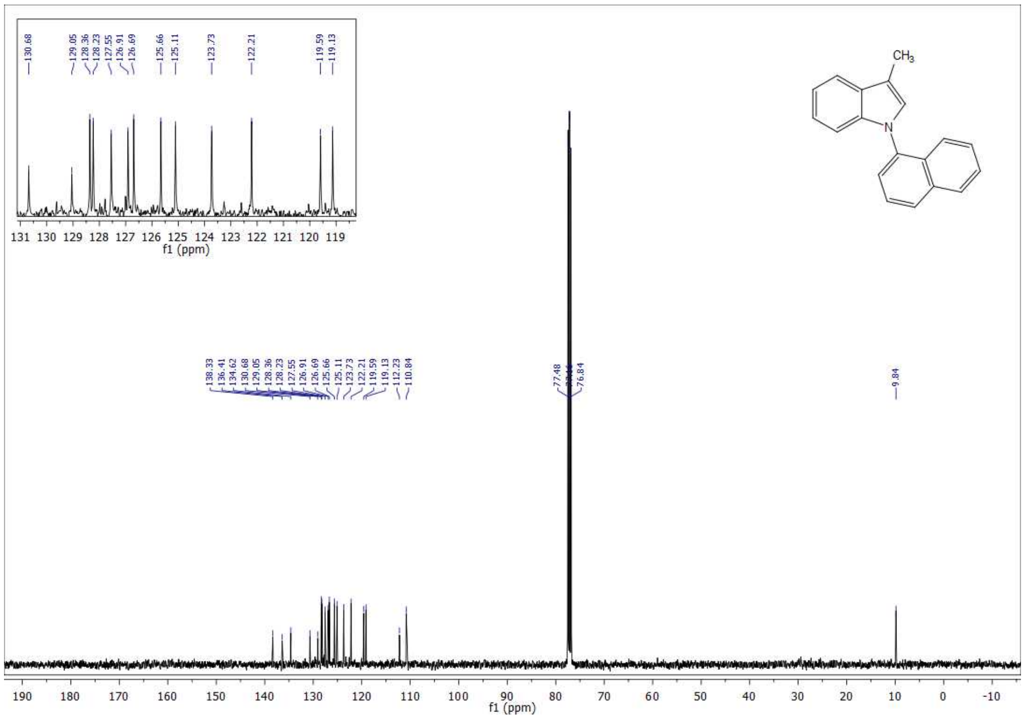

^{1}H-NMR spectrum of **3ae** (600 MHz, $CDCl_3$)

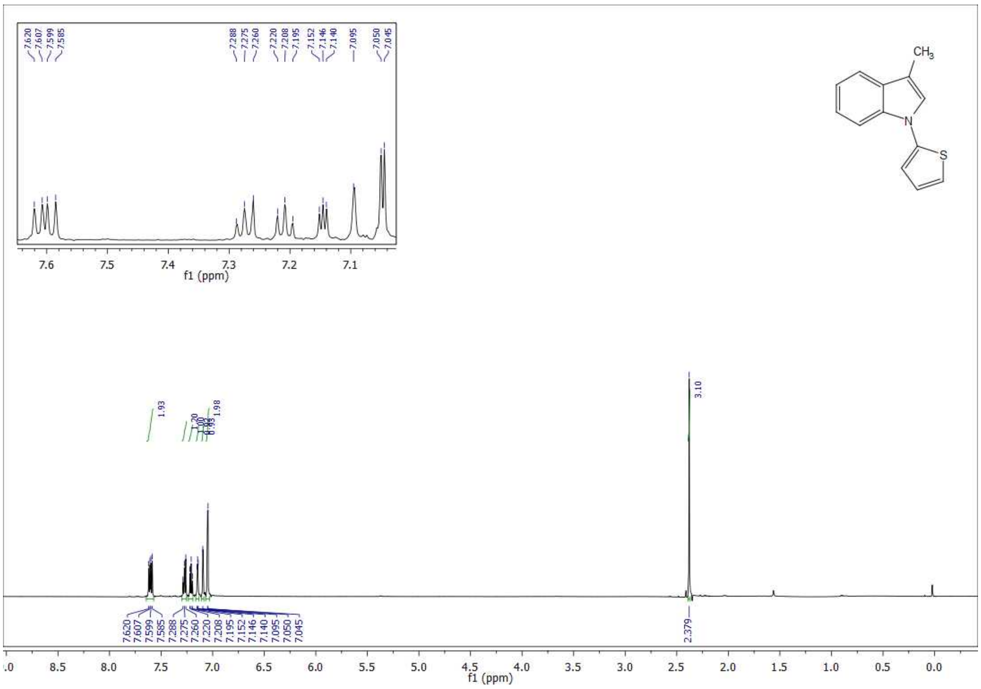

^{13}C-NMR spectrum of **3ae** (125 MHz, $CDCl_3$)

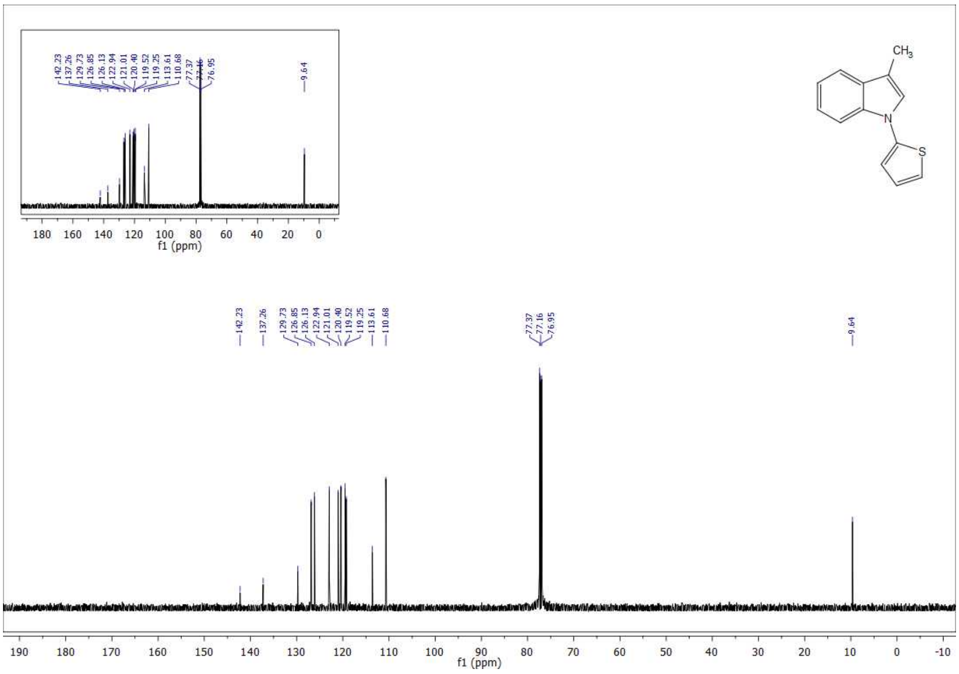

^{1}H-NMR spectrum of **3af** (400 MHz, $CDCl_3$)

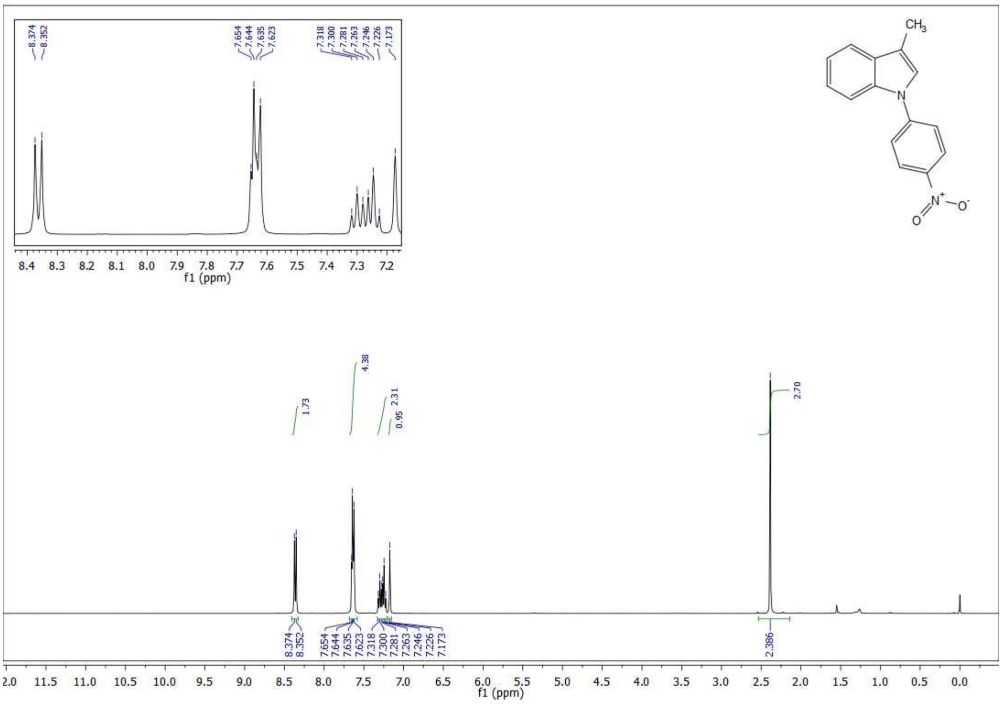

^{13}C-NMR spectrum of **3af** (100 MHz, $CDCl_3$)

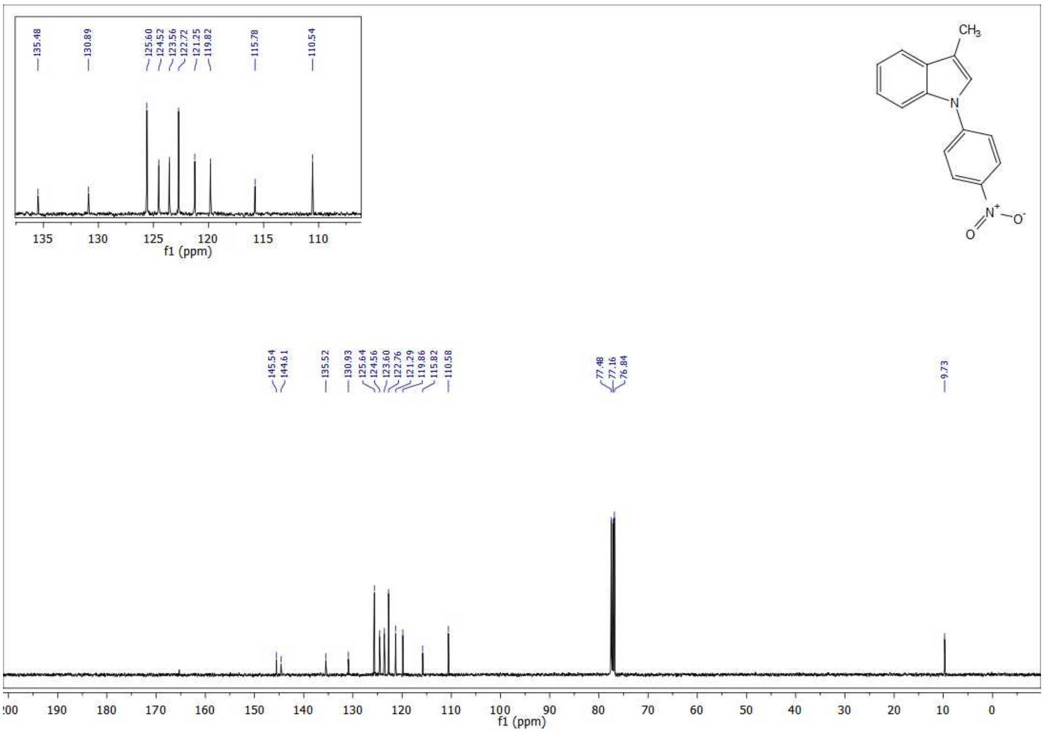

^{1}H-NMR spectrum of **3ag** (400 MHz, $CDCl_3$)

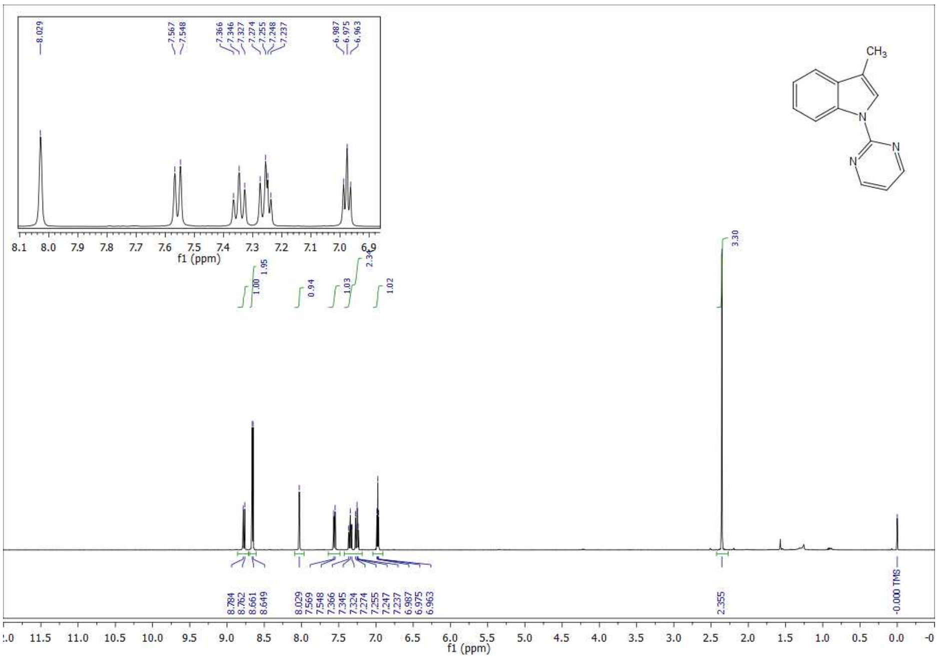

^{13}C-NMR spectrum of **3ag** (100 MHz, $CDCl_3$)

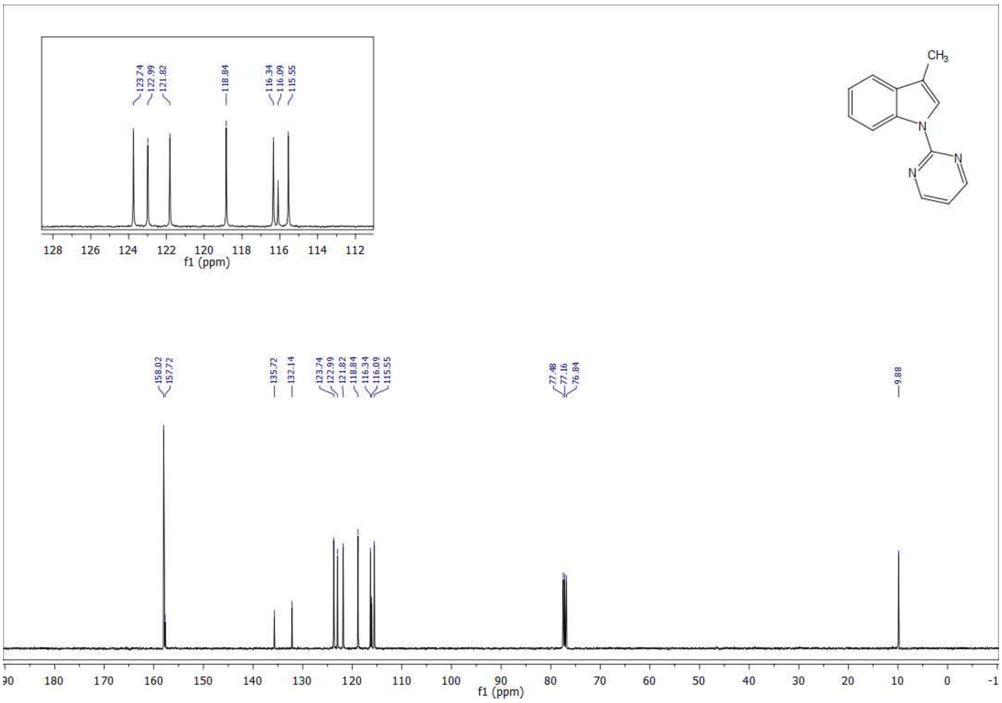

^{1}H-NMR spectrum of **3ah** (400 MHz, $CDCl_3$)

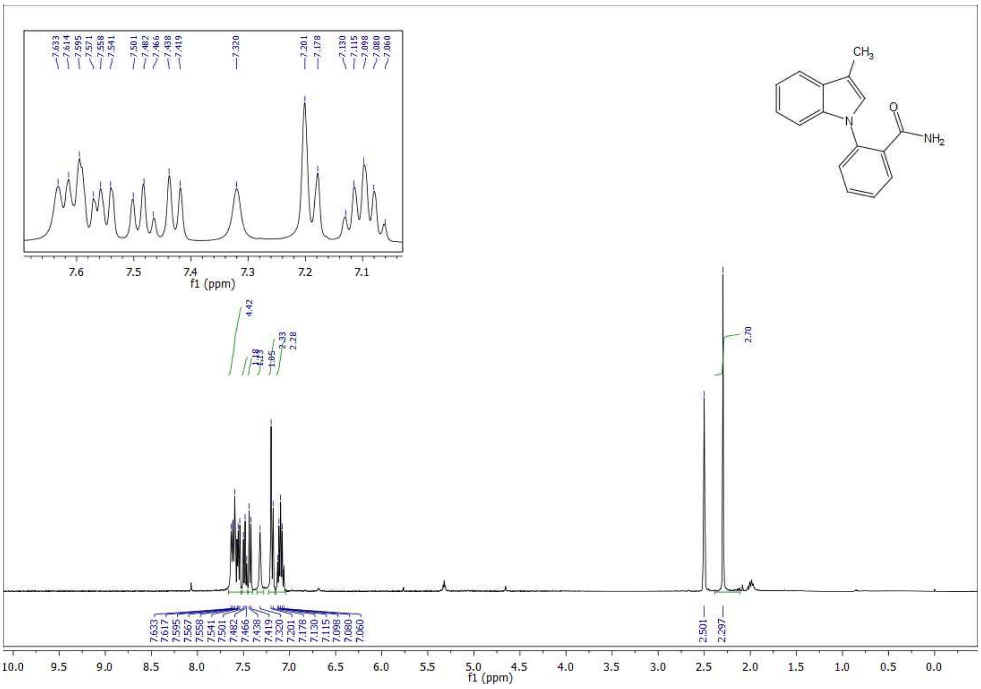

^{13}C-NMR spectrum of **3ah** (100 MHz, $CDCl_3$)

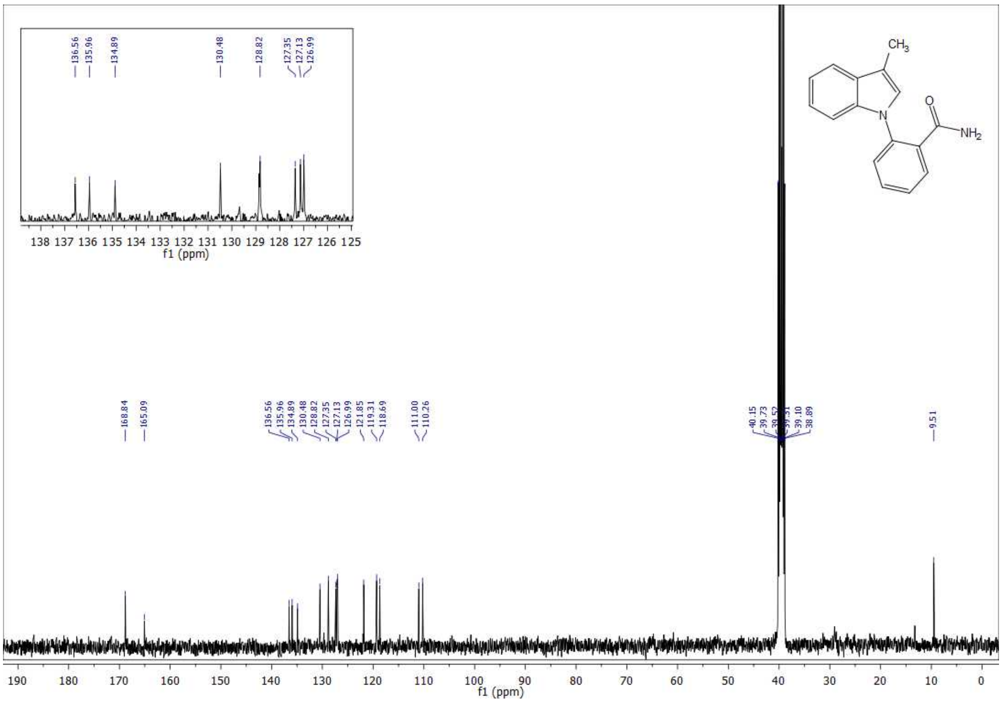

^{1}H-NMR spectrum of**3ai** (600 MHz, $CDCl_3$)

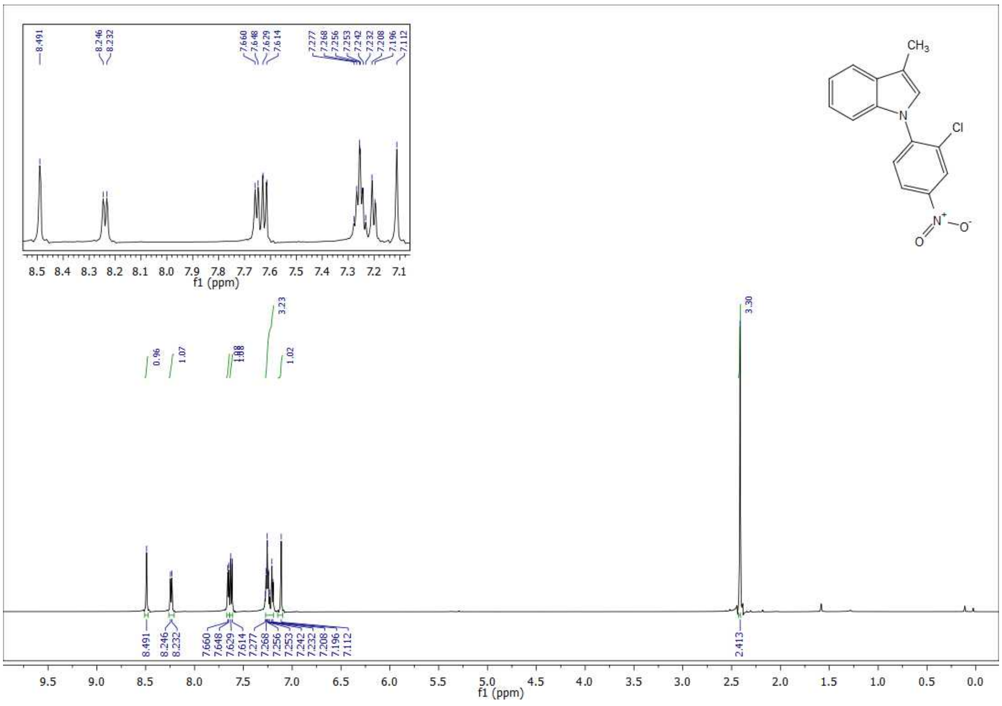

^{13}C-NMR spectrum of **3ai** (125 MHz, $CDCl_3$)

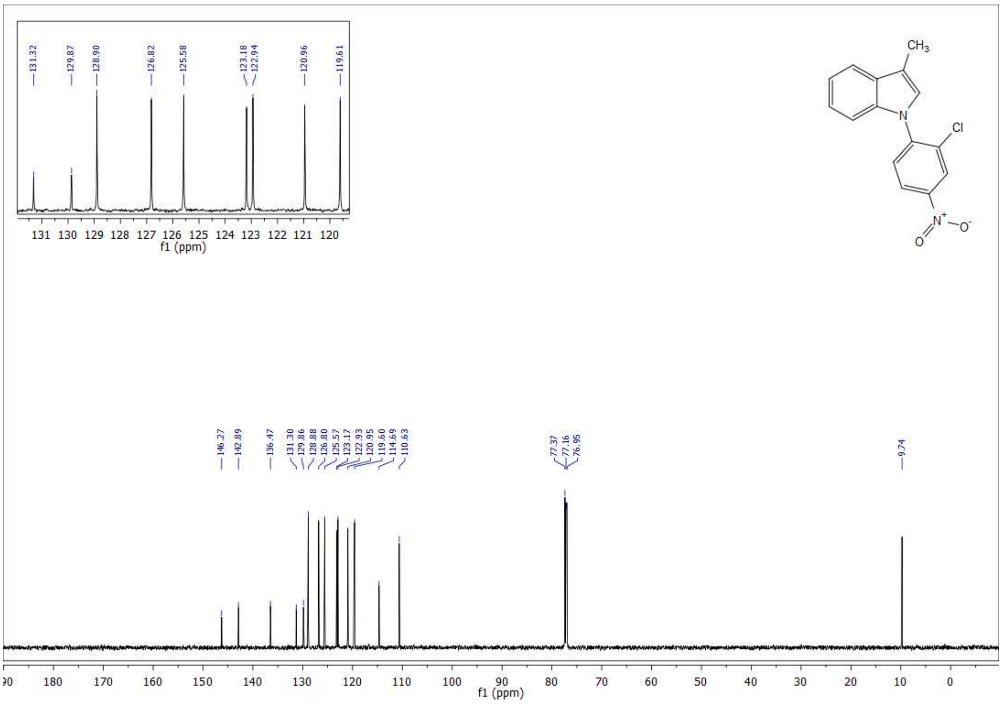

^{1}H-NMR spectrum of **3am** (400 MHz, $CDCl_3$)

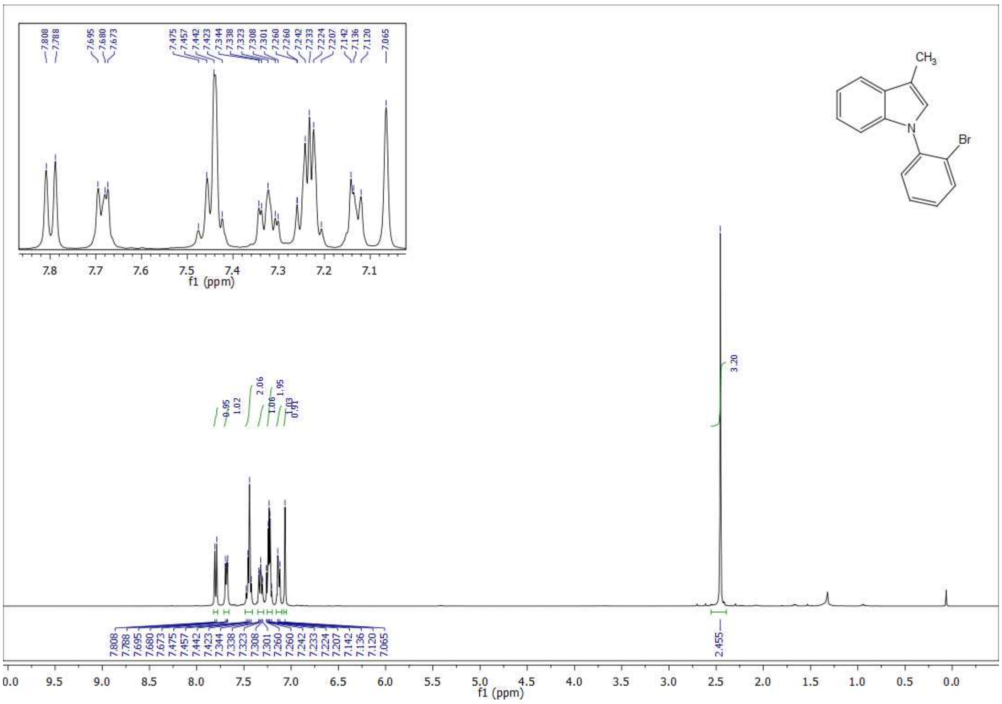

^{13}C-NMR spectrum of **3am** (100 MHz, $CDCl_3$)

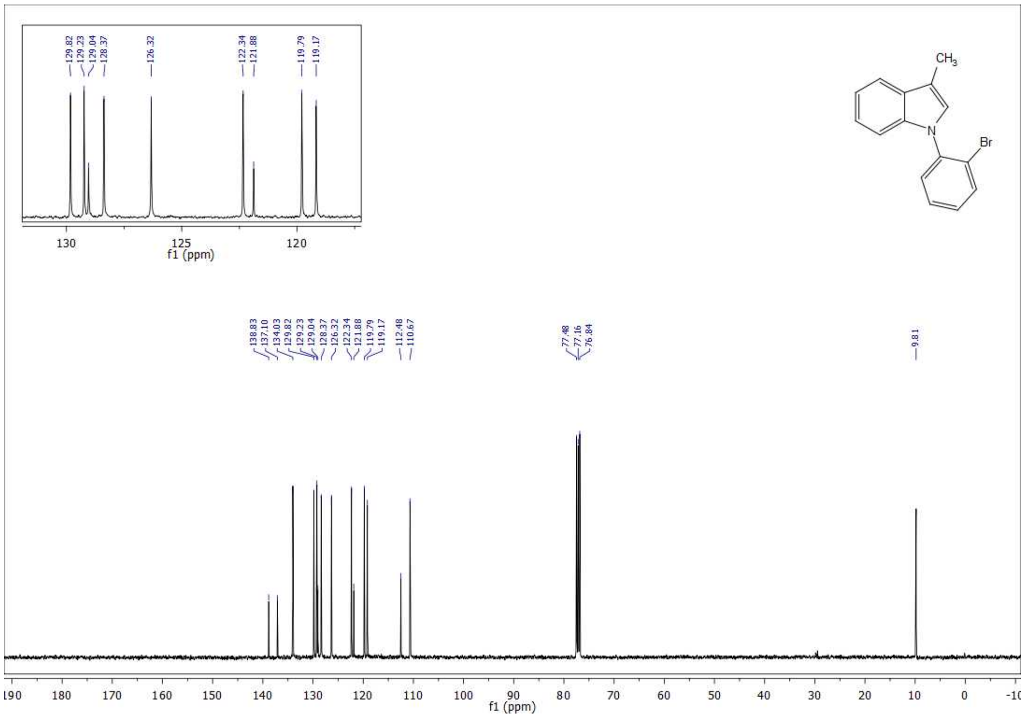

^{1}H-NMR spectrum of **3an** (400 MHz, $CDCl_3$)

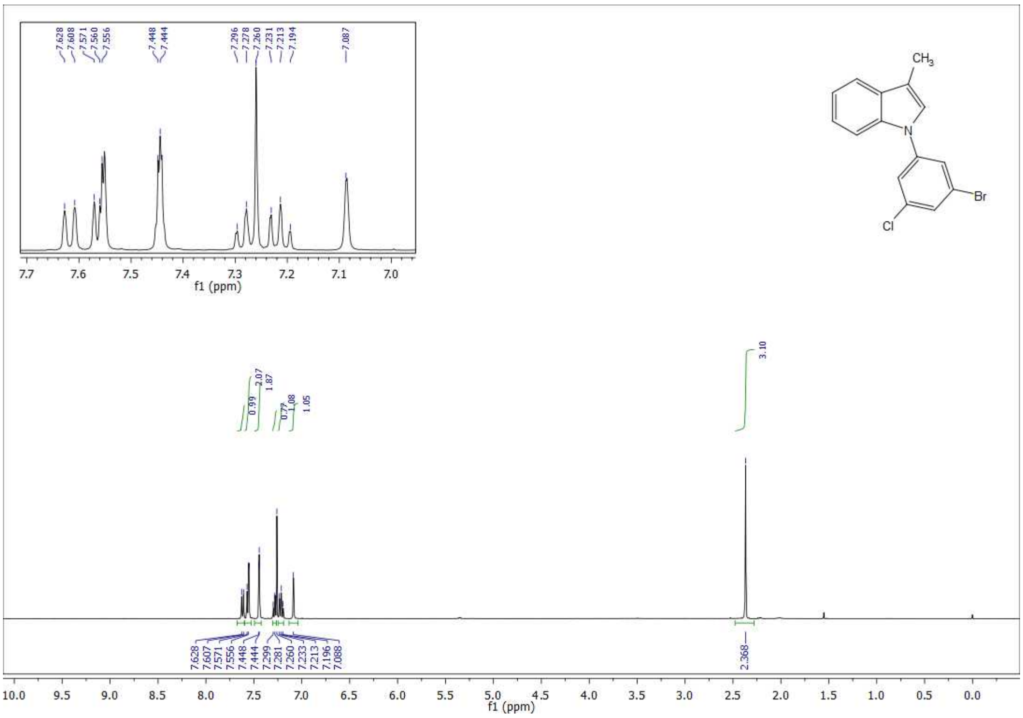

^{13}C-NMR spectrum of **3an** (100 MHz, $CDCl_3$)

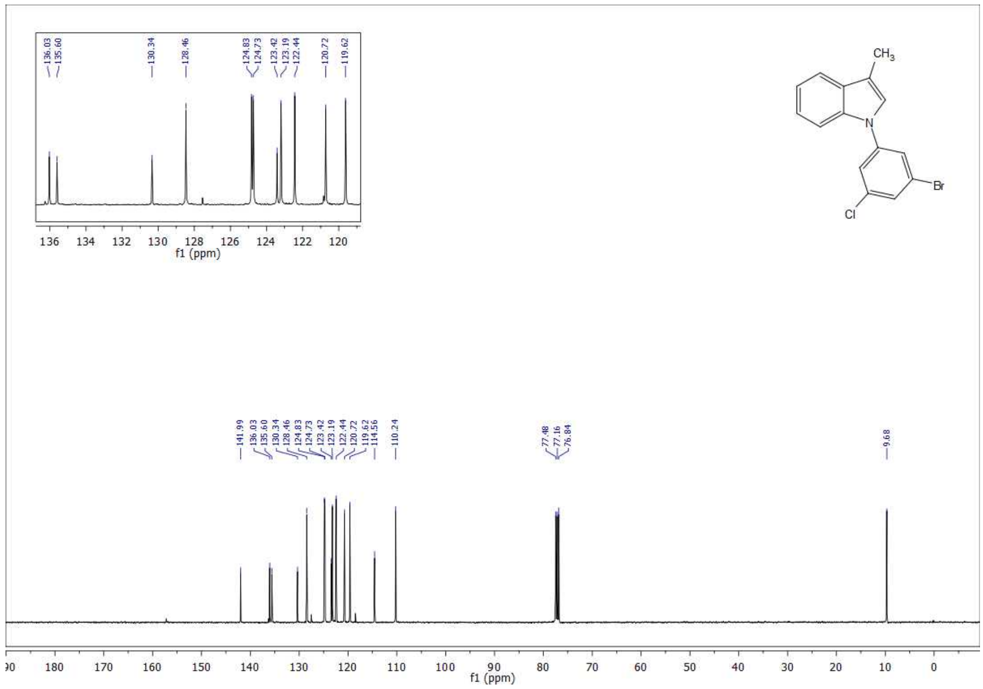

^{1}H-NMR spectrum of **3ao** (400 MHz, $CDCl_3$)

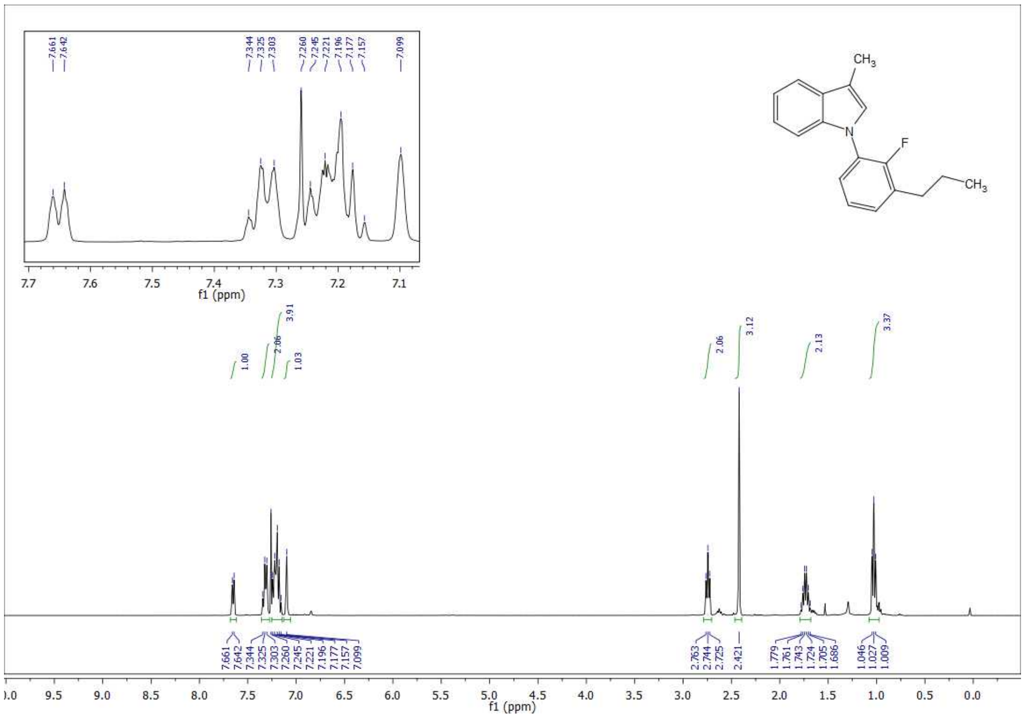

^{13}C-NMR spectrum of **3ao** (100 MHz, $CDCl_3$)

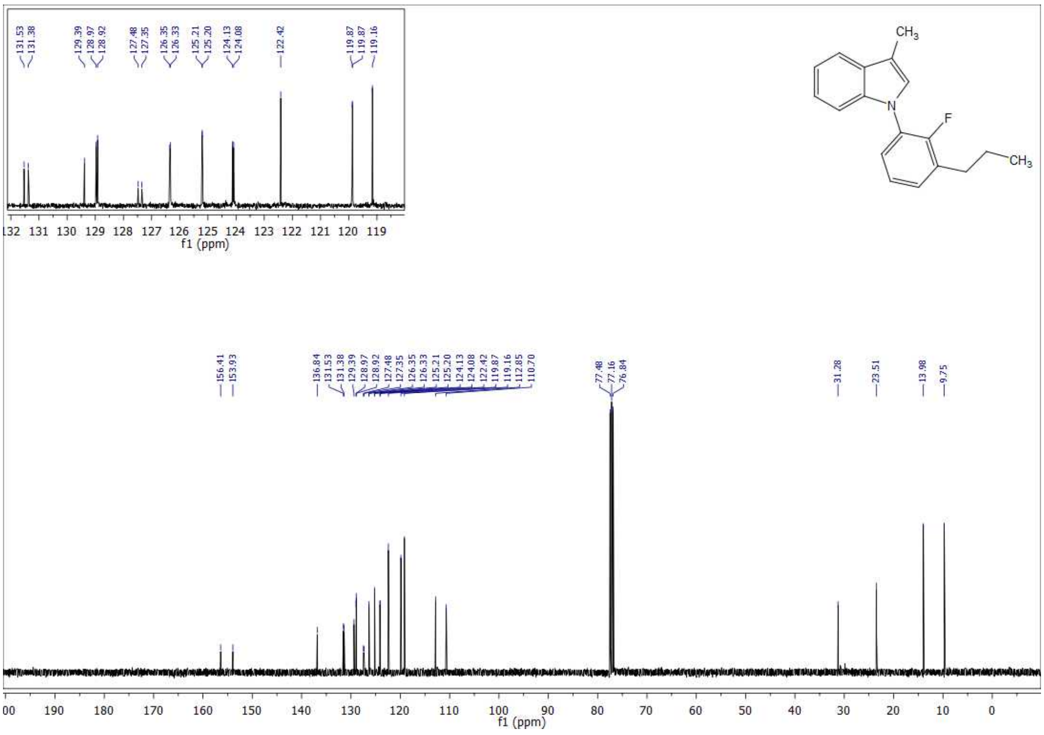

^{1}H-NMR spectrum of **3ap** (400 MHz, $CDCl_3$)

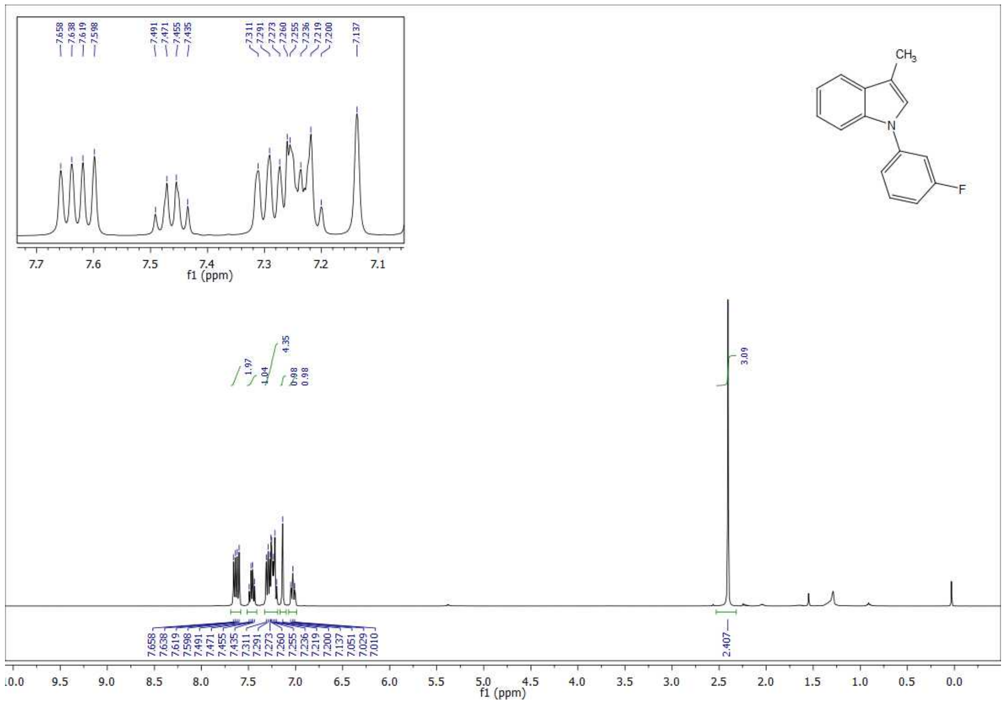

^{13}C-NMR spectrum of **3ap** (100 MHz, $CDCl_3$)

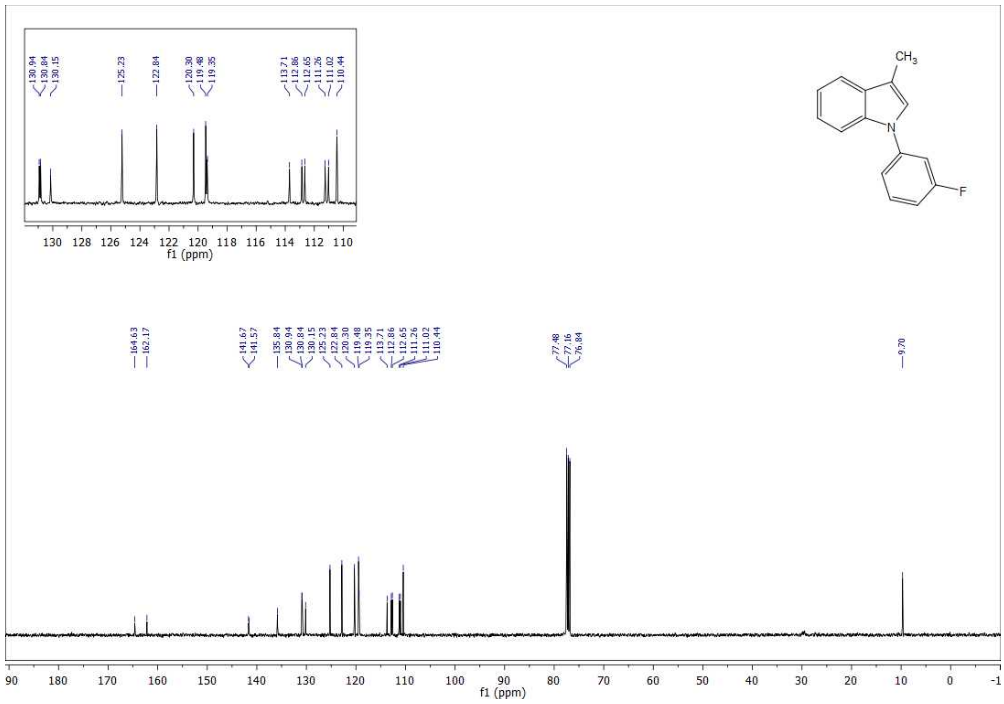

^{1}H-NMR spectrum of **3aq** (400 MHz, $CDCl_3$)

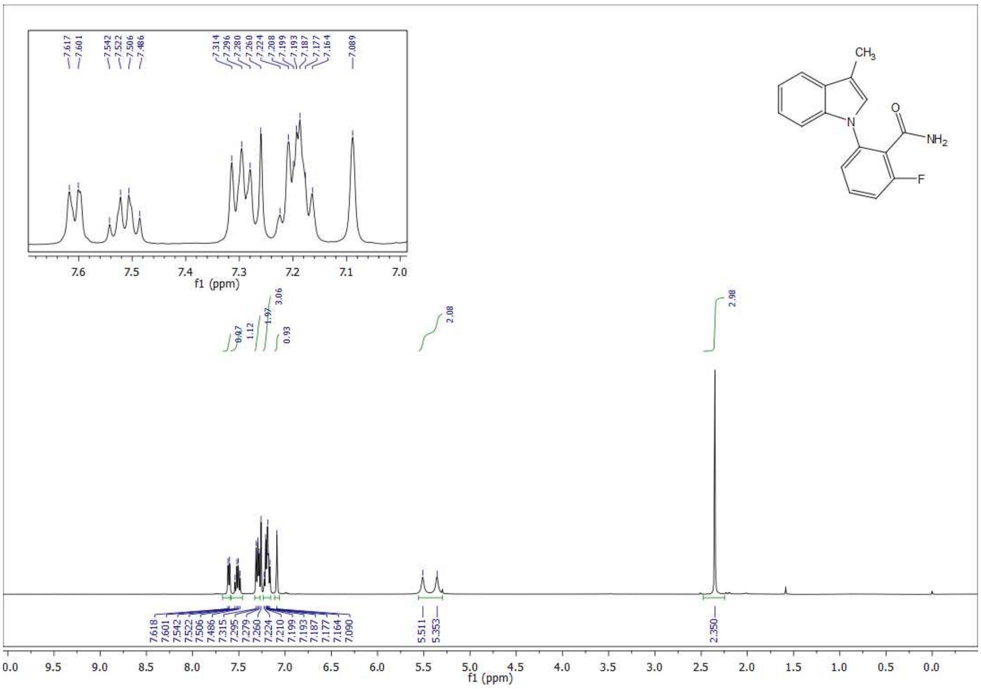

^{13}C-NMR spectrum of **3aq** (100 MHz, $CDCl_3$)

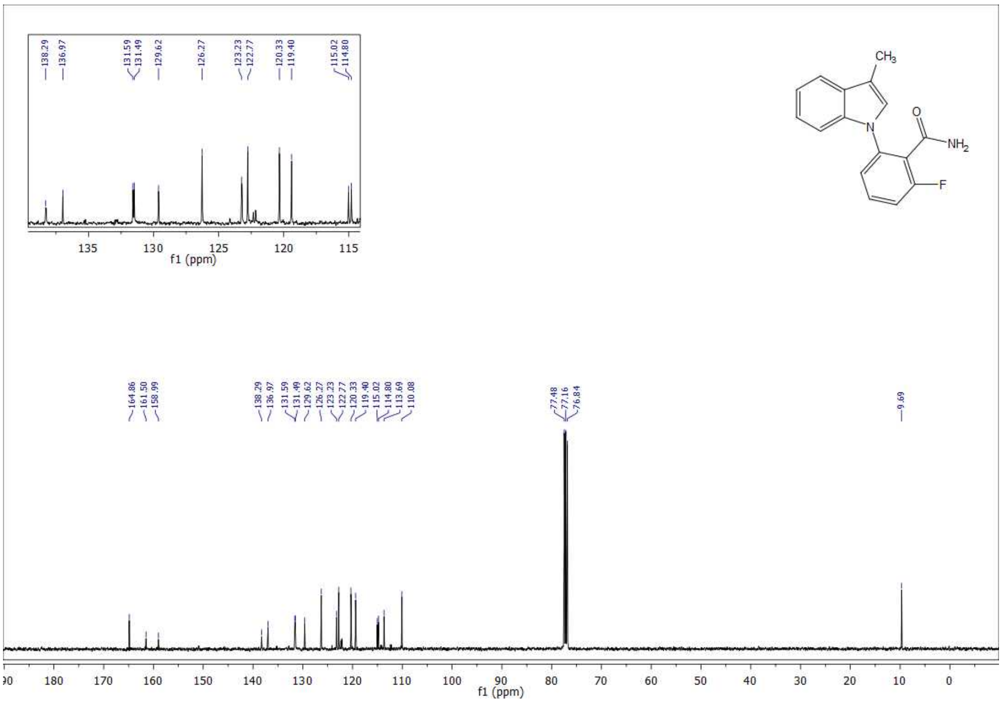

^{1}H-NMR spectrum of **3ba** (400 MHz, $CDCl_3$)

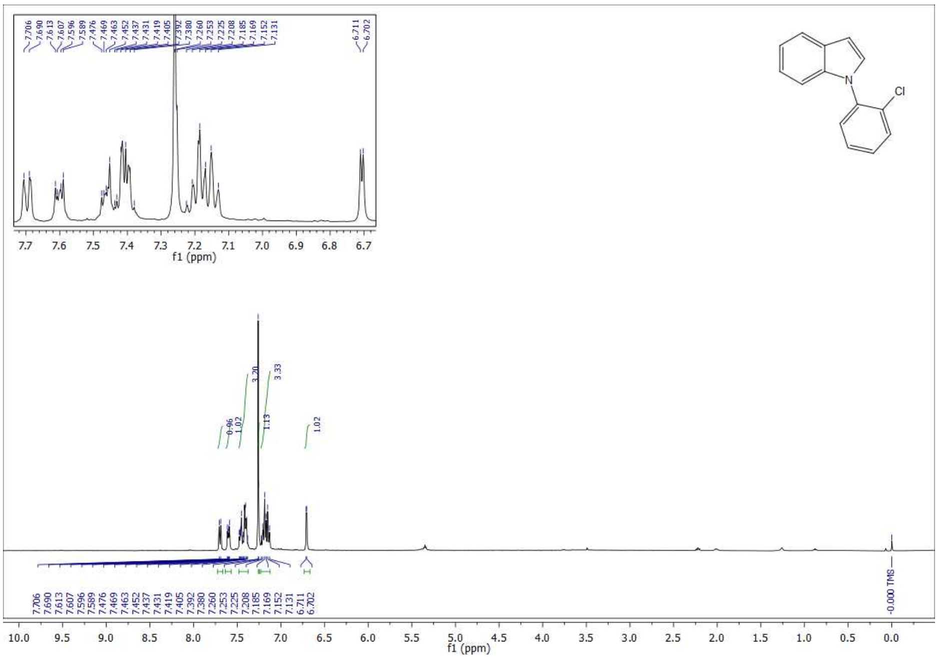

^{13}C-NMR spectrum of **3ba** (100 MHz, $CDCl_3$)

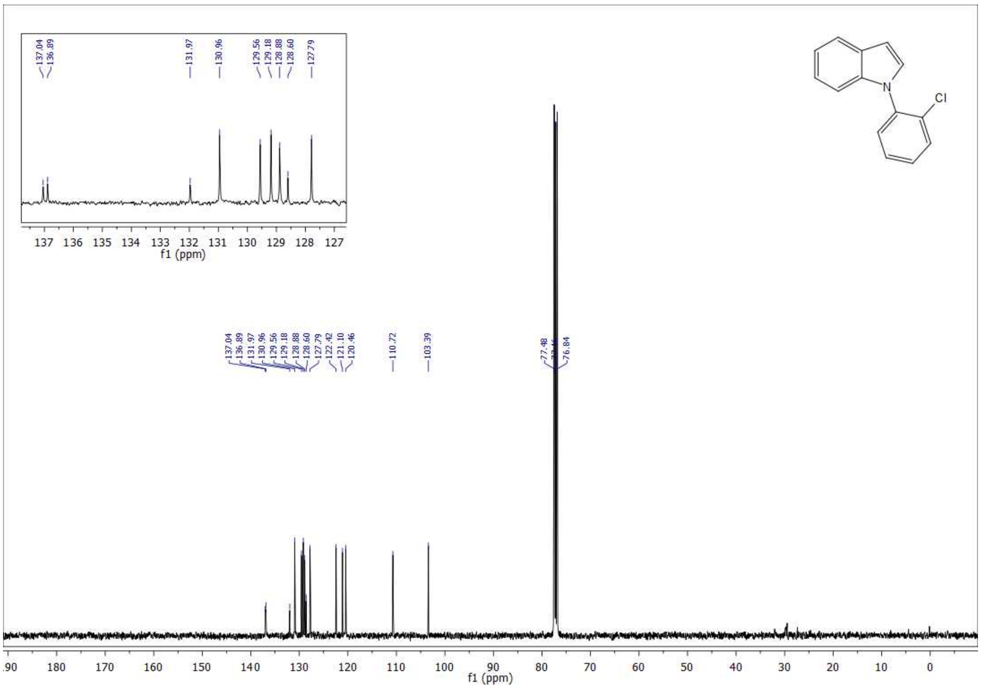

^{1}H-NMR spectrum of **3bb** (400 MHz, $CDCl_3$)

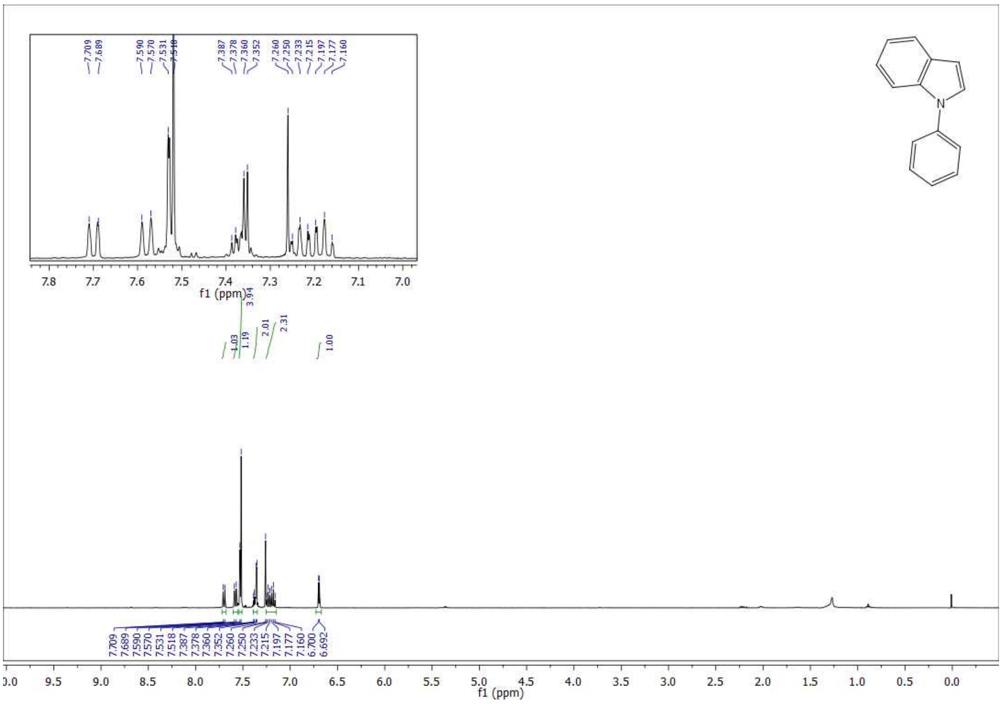

^{13}C-NMR spectrum of **3bb** (100 MHz, $CDCl_3$)

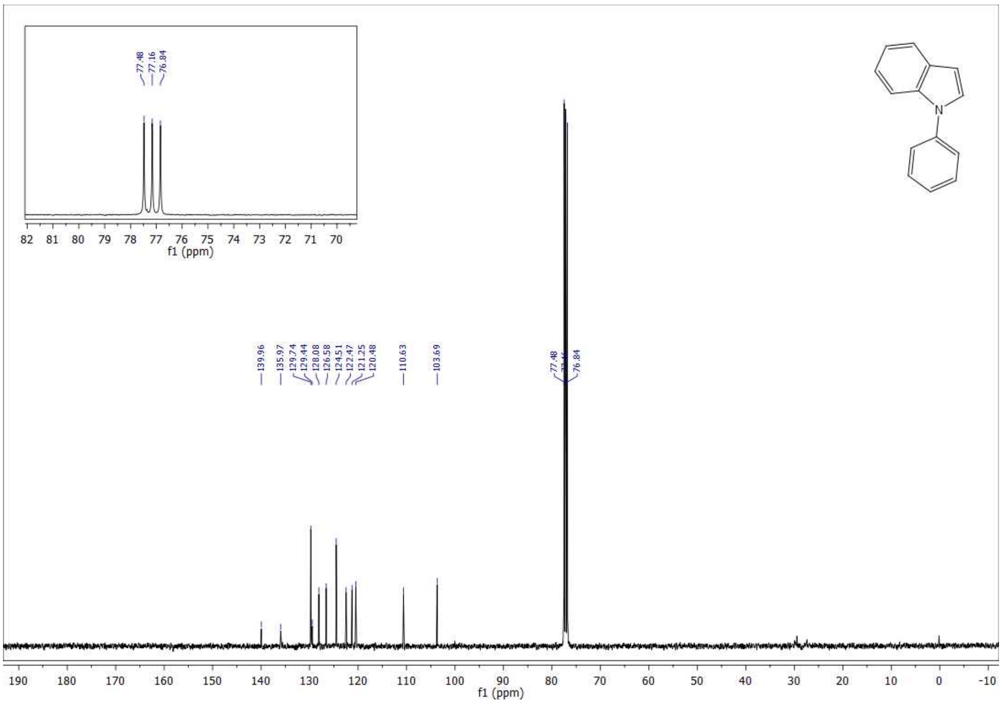

^{1}H-NMR spectrum of **3ca** (400 MHz, $CDCl_3$)

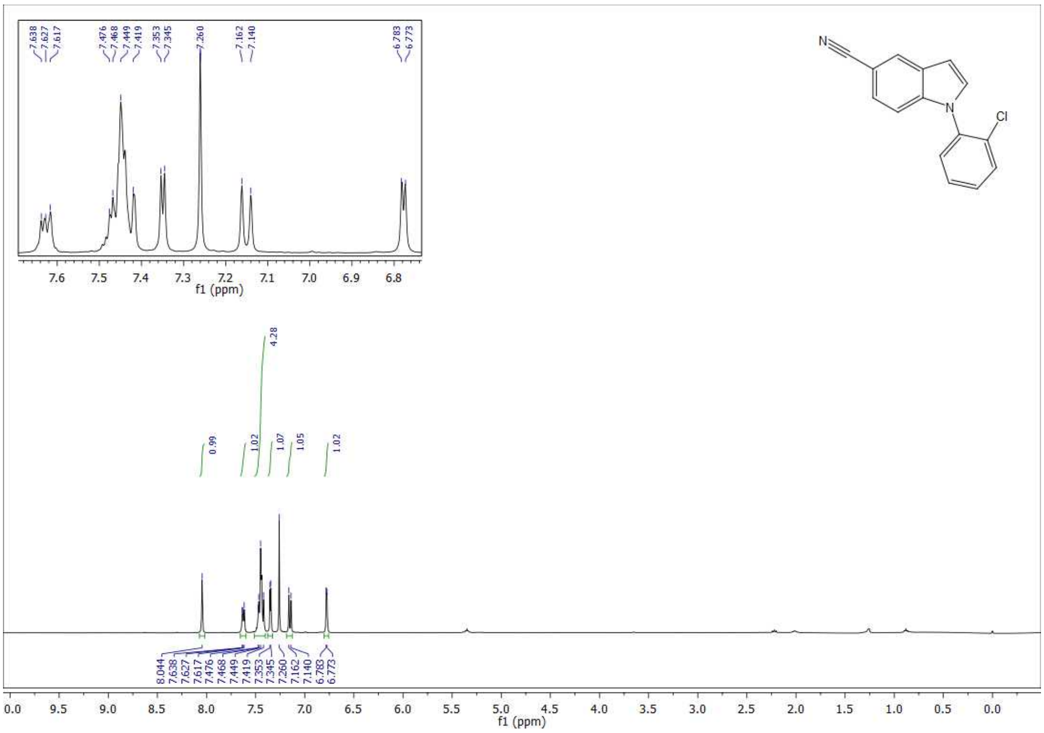

^{13}C-NMR spectrum of **3ca** (100 MHz, $CDCl_3$)

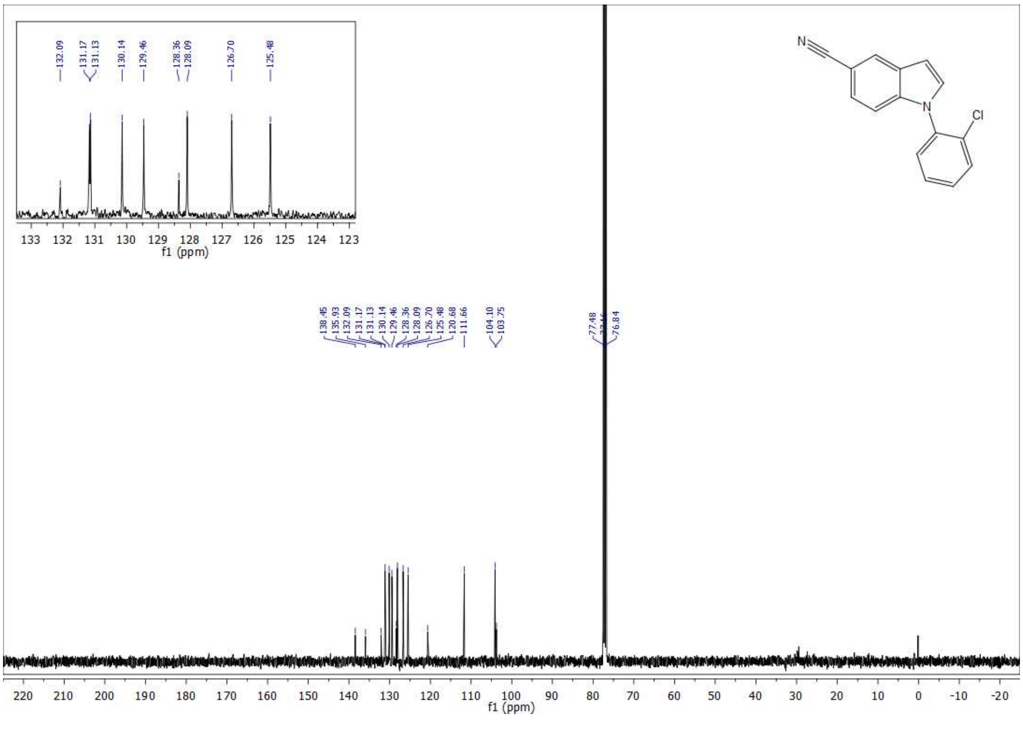

^{1}H-NMR spectrum of **3da** (400 MHz, $CDCl_3$)

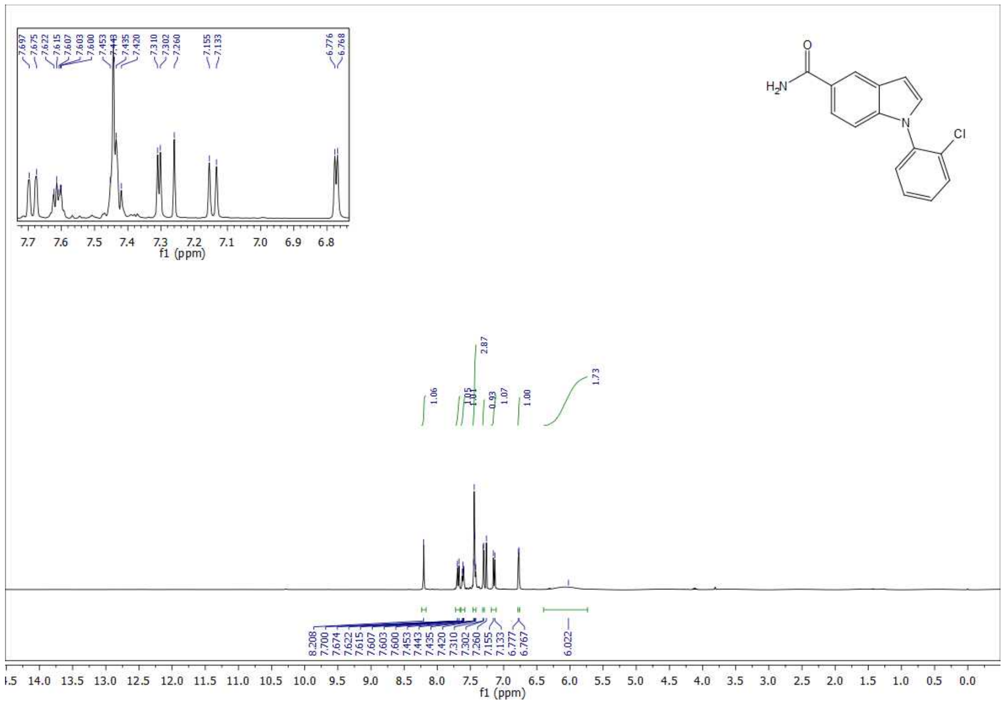

^{13}C-NMR spectrum of **3da** (100 MHz, $CDCl_3$)

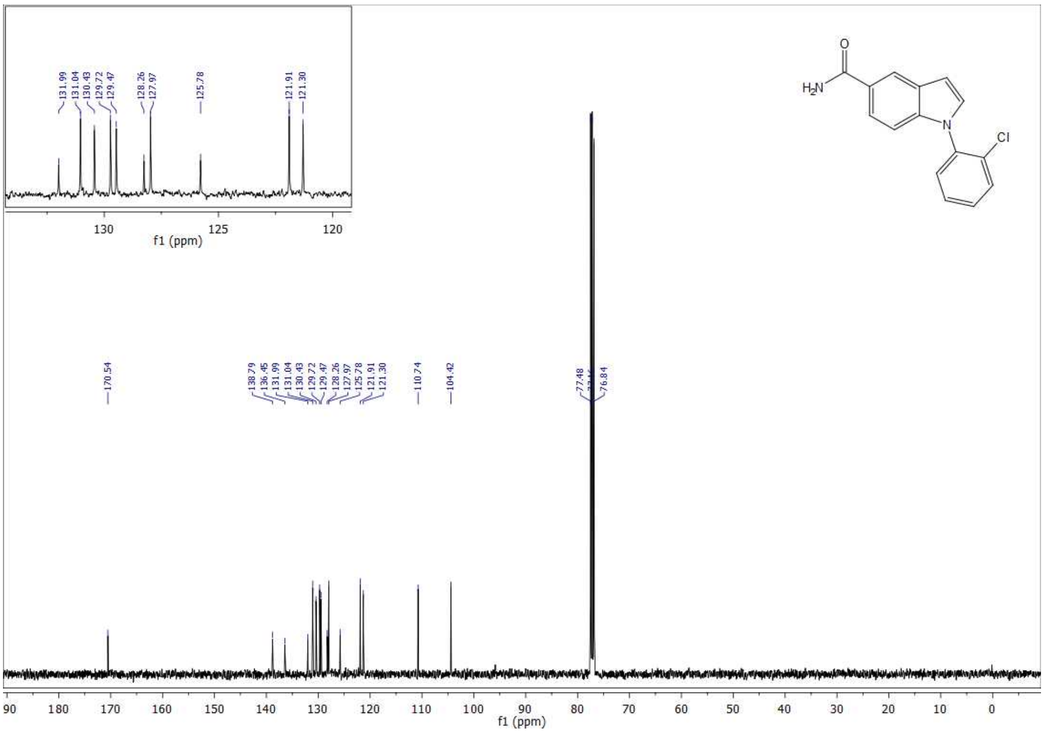

^{1}H-NMR spectrum of **3eb** (400 MHz, $CDCl_3$)

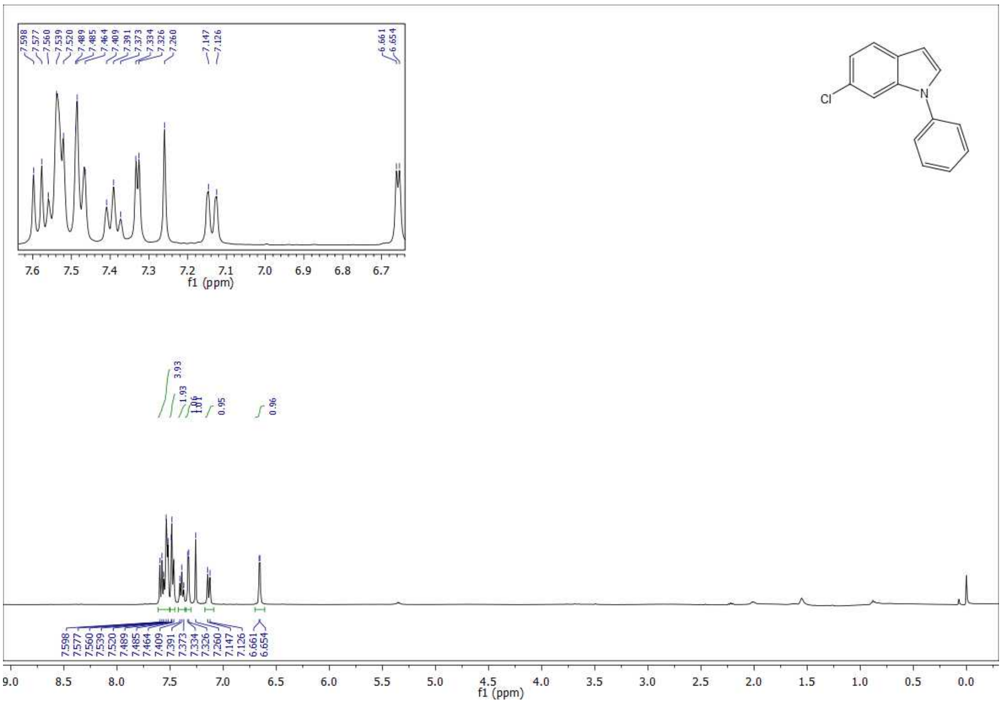

^{13}C-NMR spectrum of **3eb** (100 MHz, $CDCl_3$)

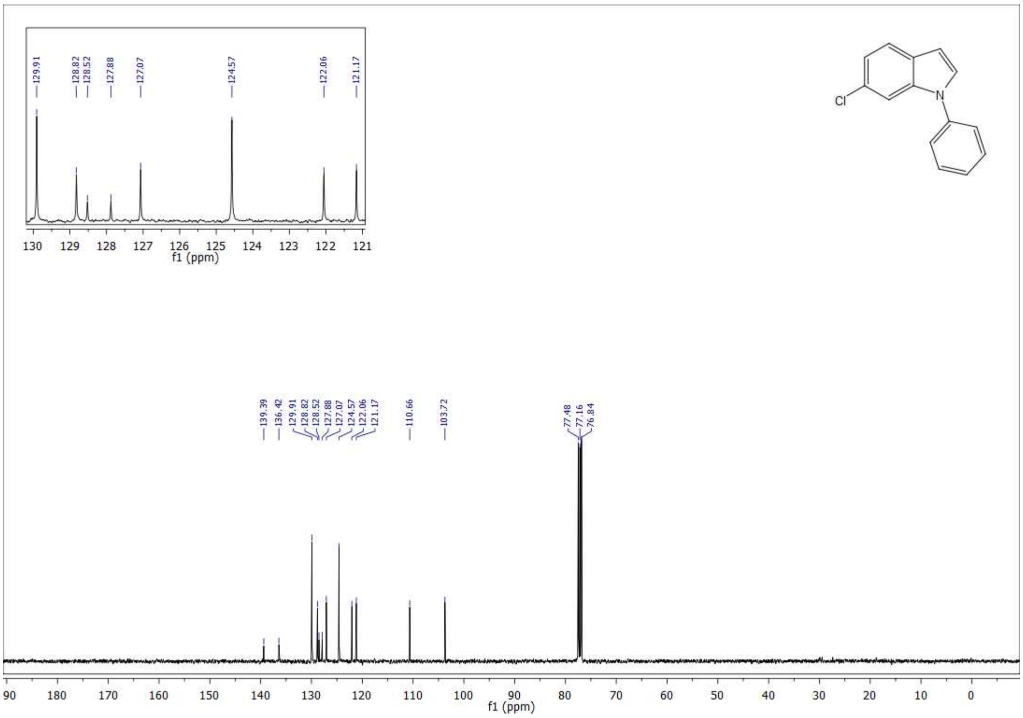

^{1}H-NMR spectrum of **3fb** (400 MHz, $CDCl_3$)

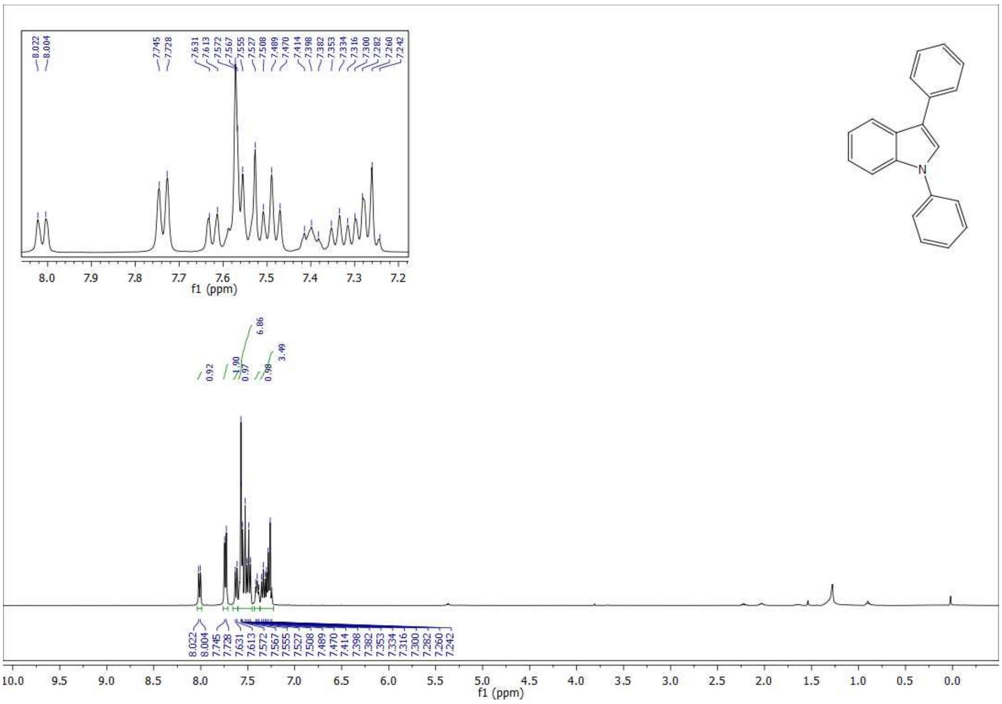

^{13}C-NMR spectrum of **3fb** (100 MHz, $CDCl_3$)

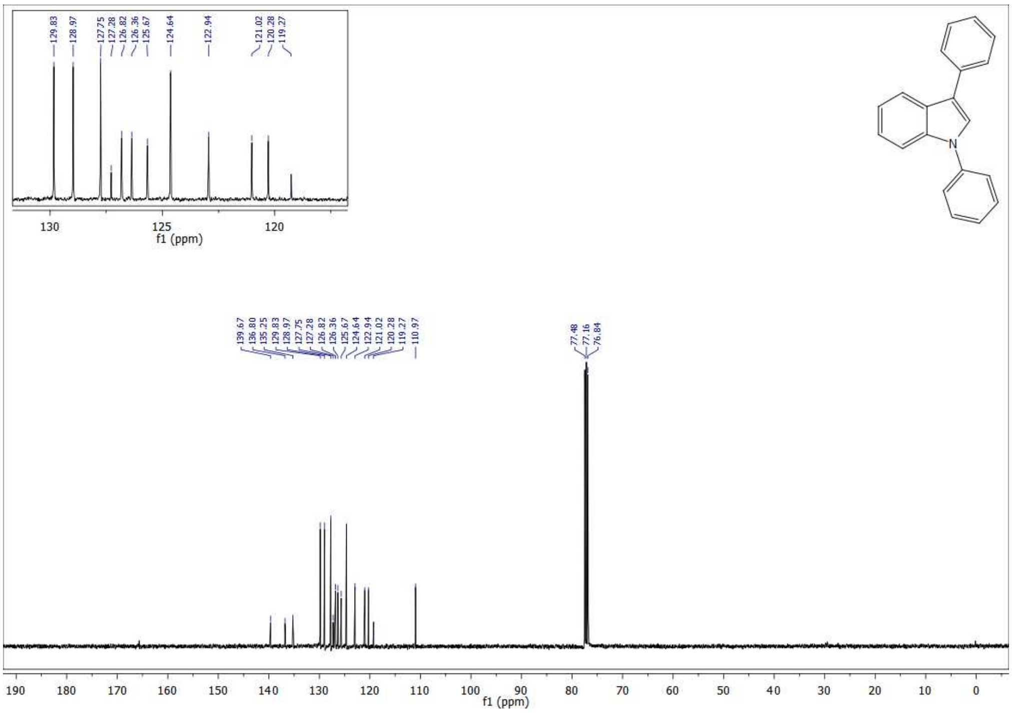

^{1}H-NMR spectrum of **5a** (400 MHz, $CDCl_3$)

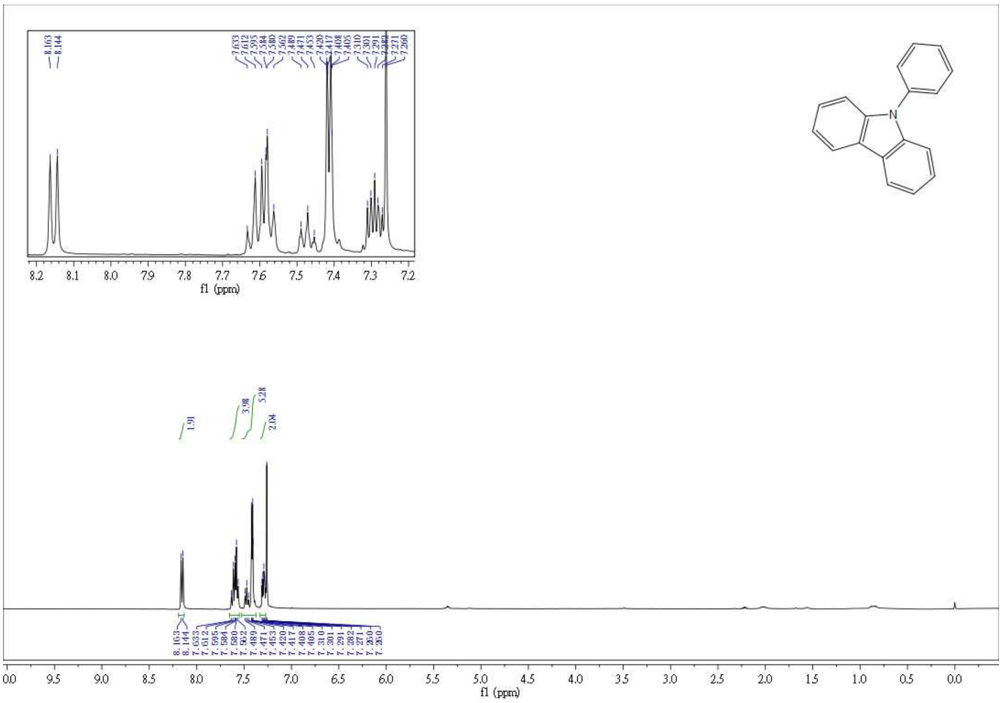

^{13}C-NMR spectrum of **5a** (100 MHz, $CDCl_3$)

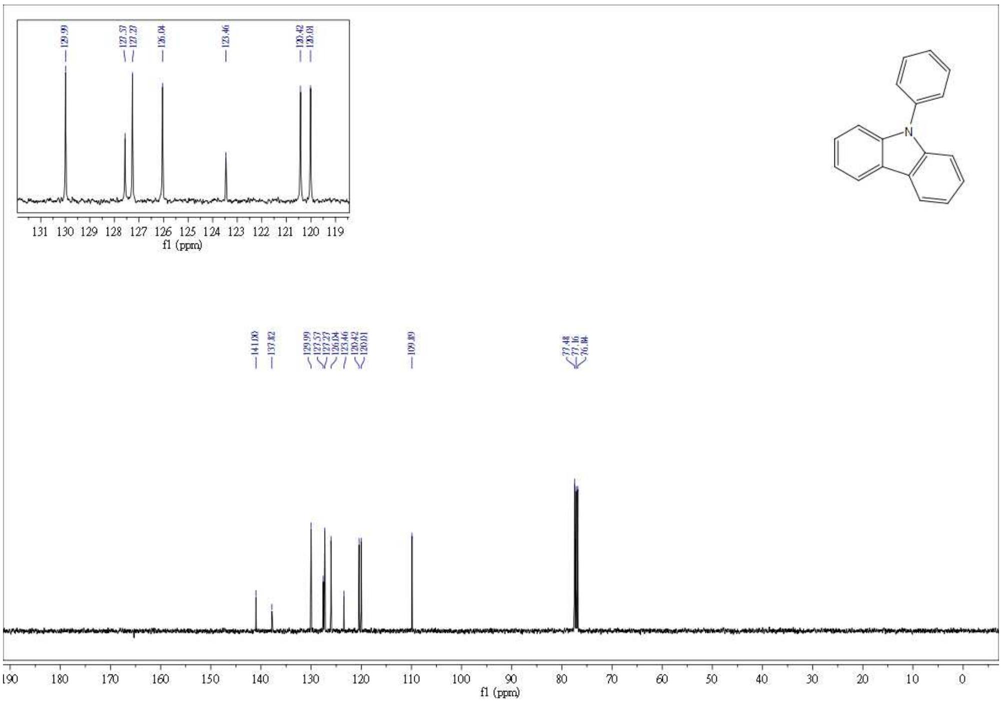

^{1}H-NMR spectrum of **5b** (400 MHz, $CDCl_3$)

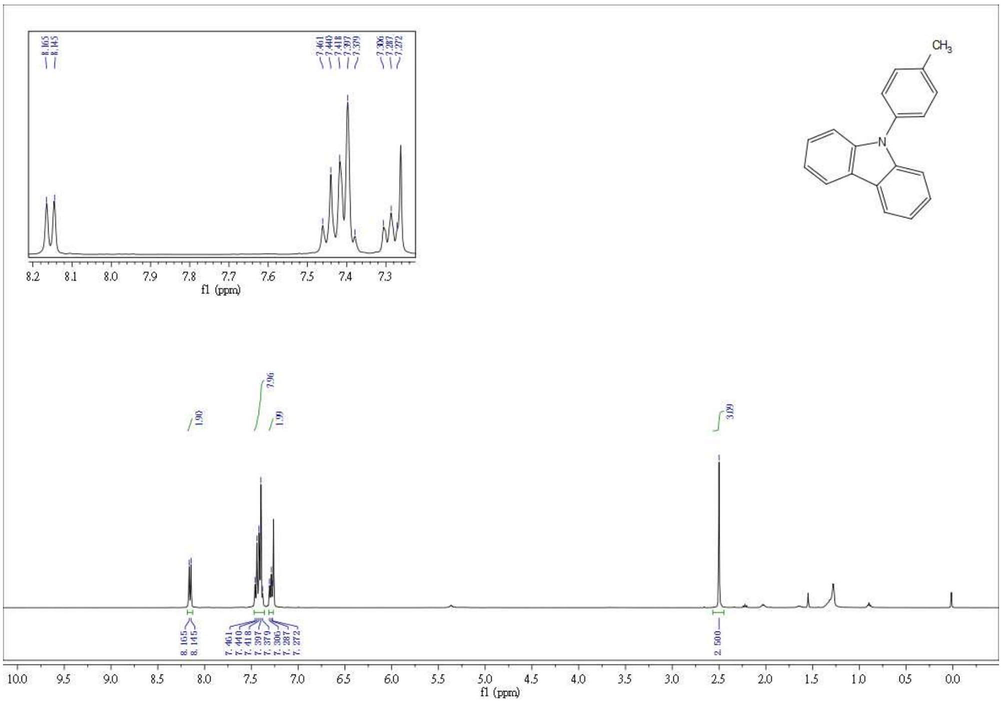

^{13}C-NMR spectrum of **5b** (100 MHz, $CDCl_3$)

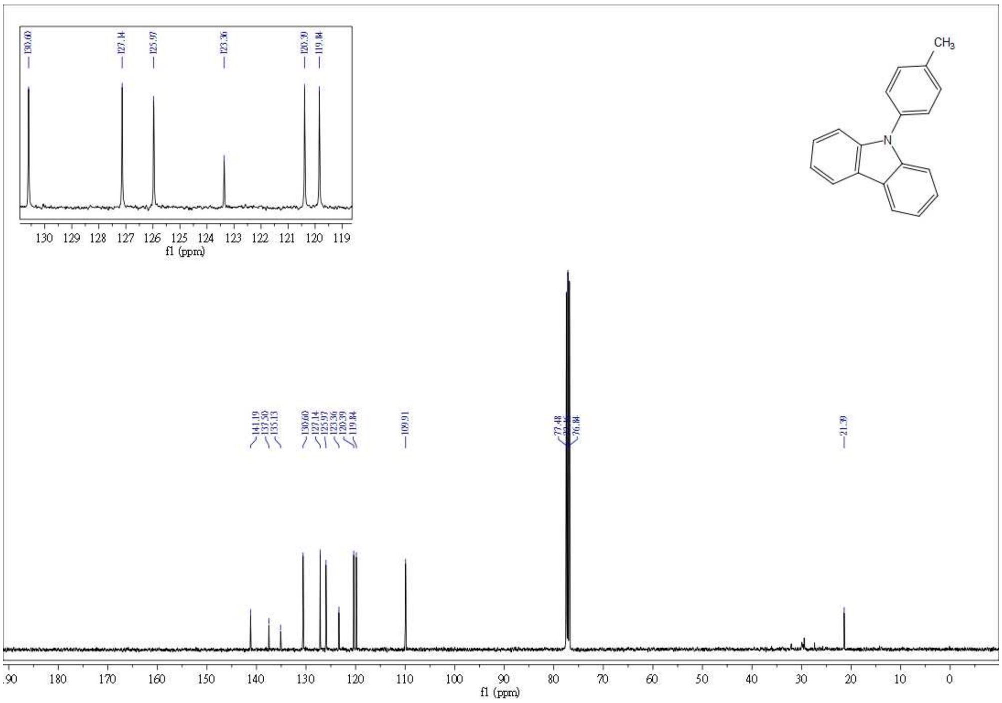

^{1}H-NMR spectrum of **5c** (400 MHz, $CDCl_3$)

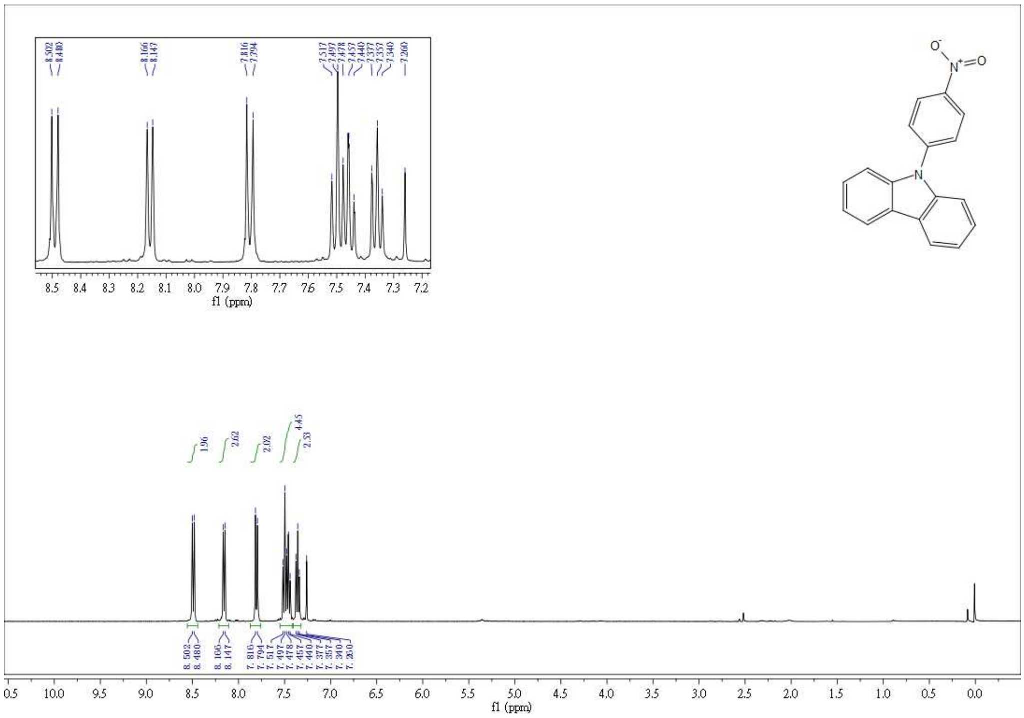

^{13}C-NMR spectrum of **5c** (100 MHz, $CDCl_3$)

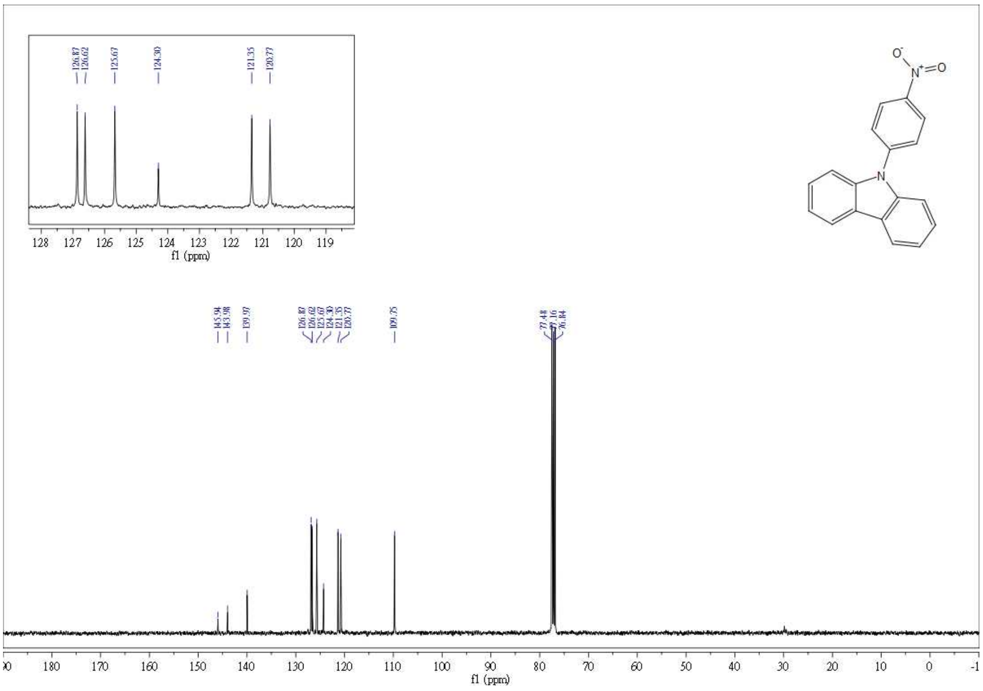

^{1}H-NMR spectrum of **5d** (400 MHz, $CDCl_3$)

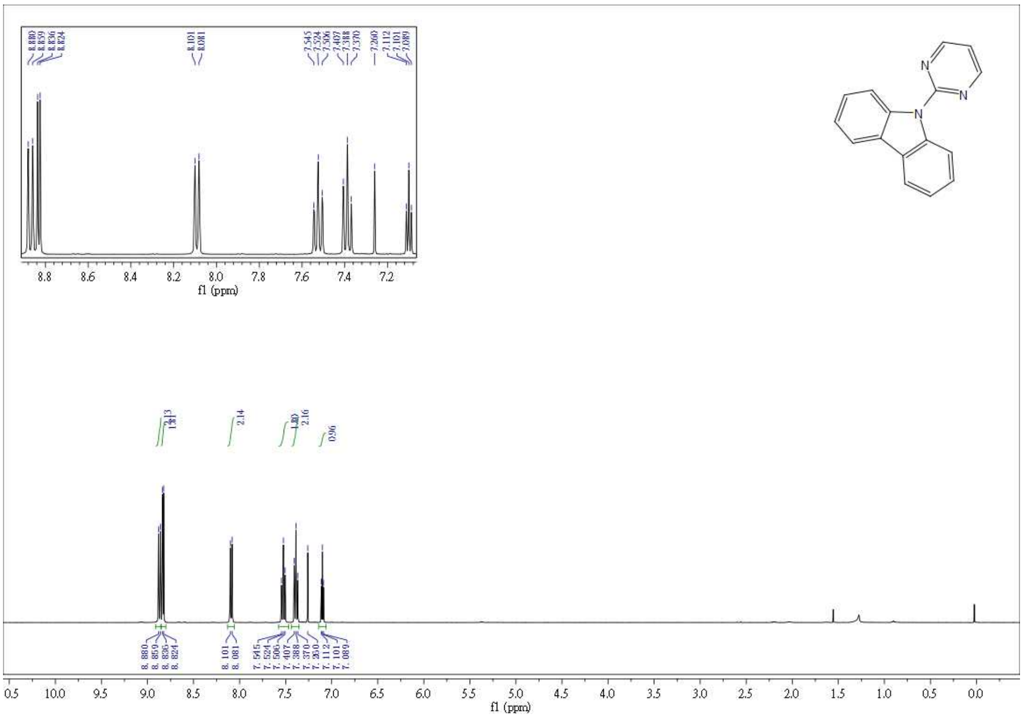

^{13}C-NMR spectrum of **5d** (100 MHz, $CDCl_3$)

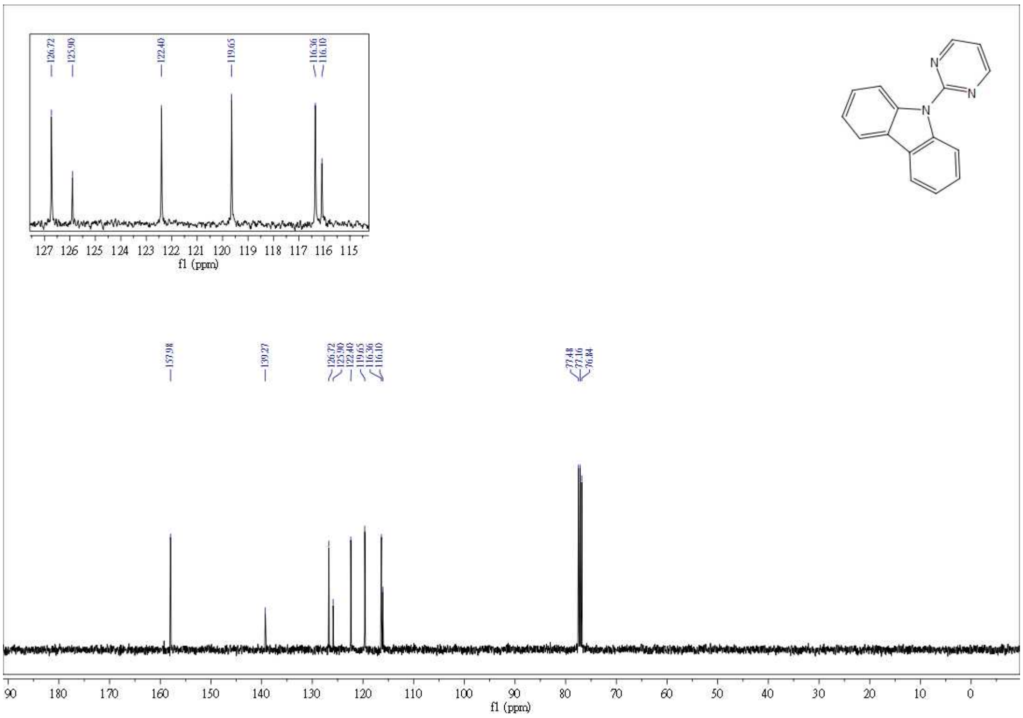

^{1}H-NMR spectrum of **5e** (400 MHz, $CDCl_3$)

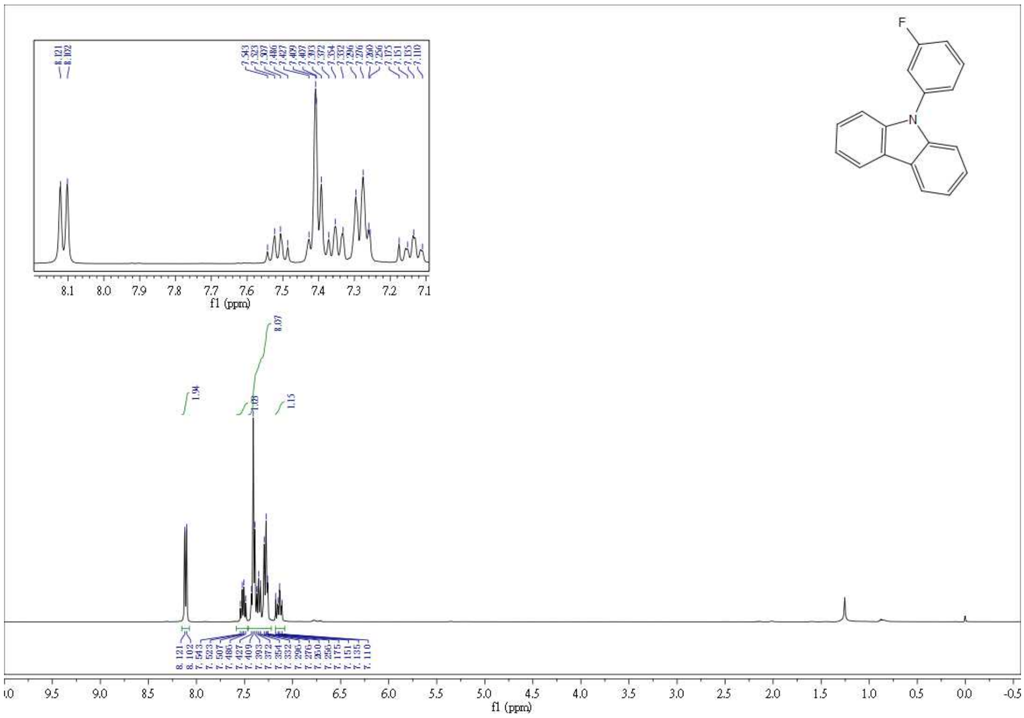

^{13}C-NMR spectrum of **5e** (100 MHz, $CDCl_3$)

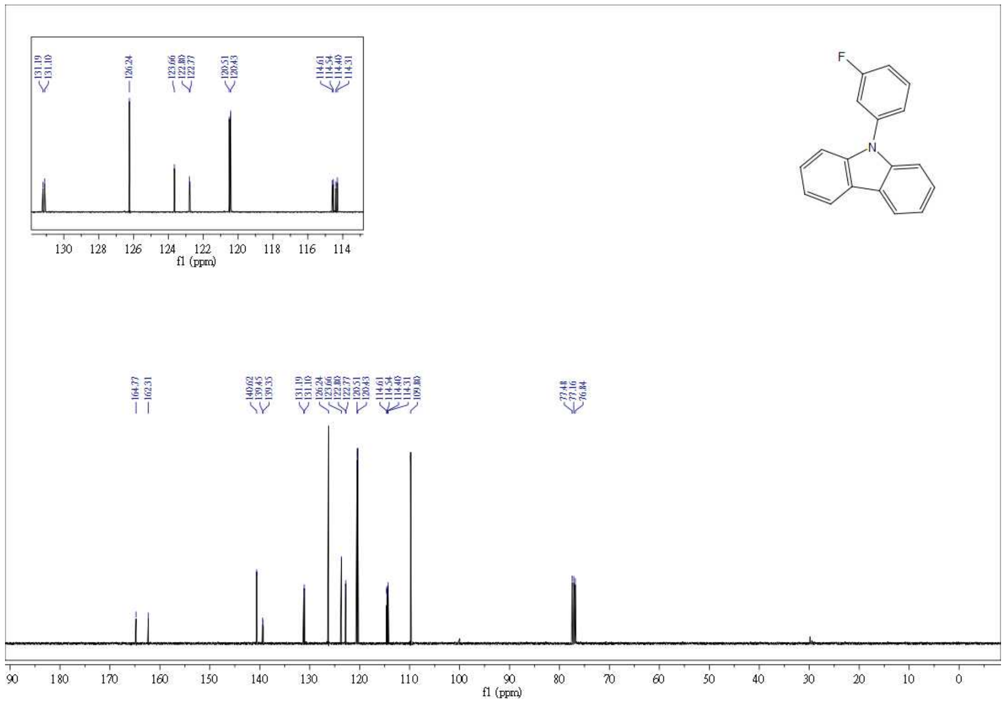

^{1}H-NMR spectrum of **5f** (400 MHz, DMSO-d_6)

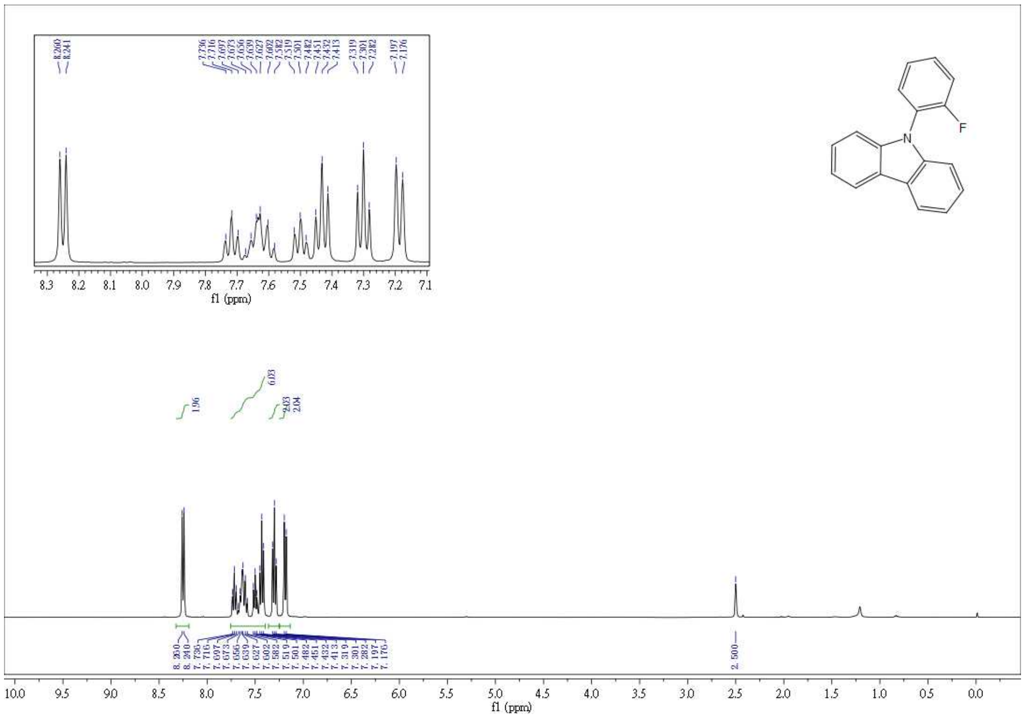

^{13}C-NMR spectrum of **5f** (100 MHz, DMSO-d_6)

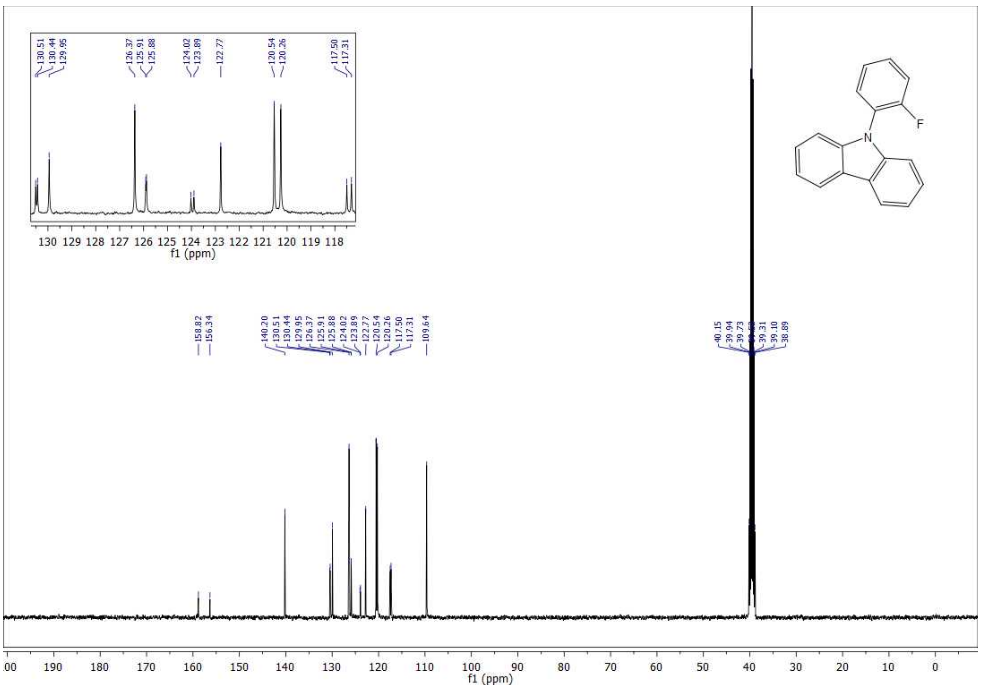

^{1}H-NMR spectrum of **5g** (400 MHz, DMSO-*d*6)

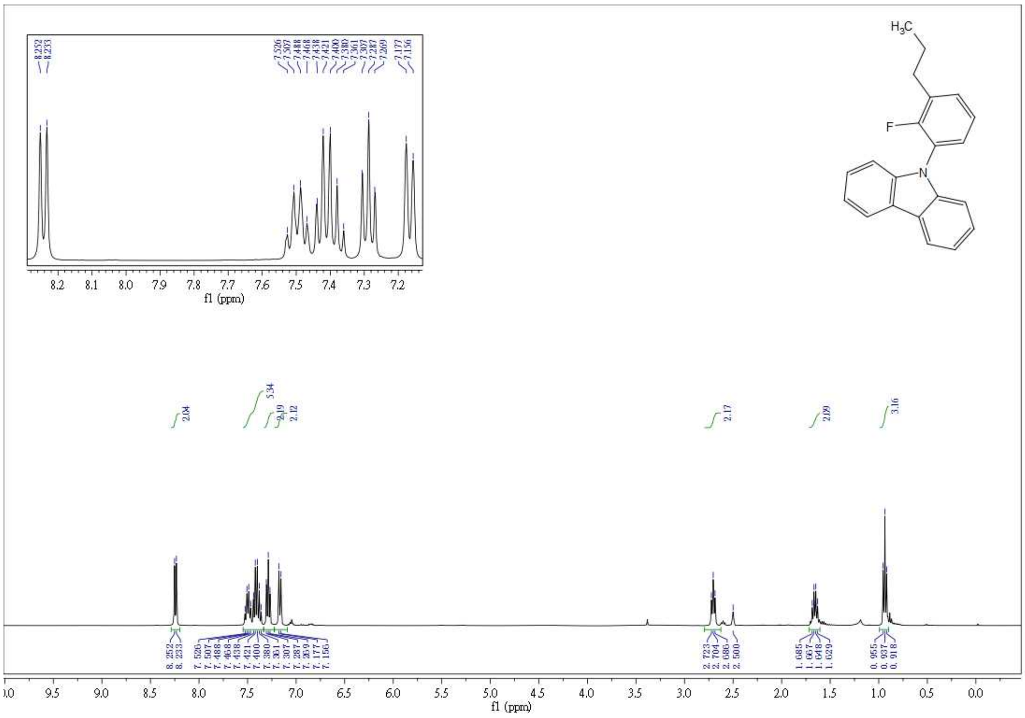

^{13}C-NMR spectrum of **5g** (100 MHz, DMSO-*d*6)

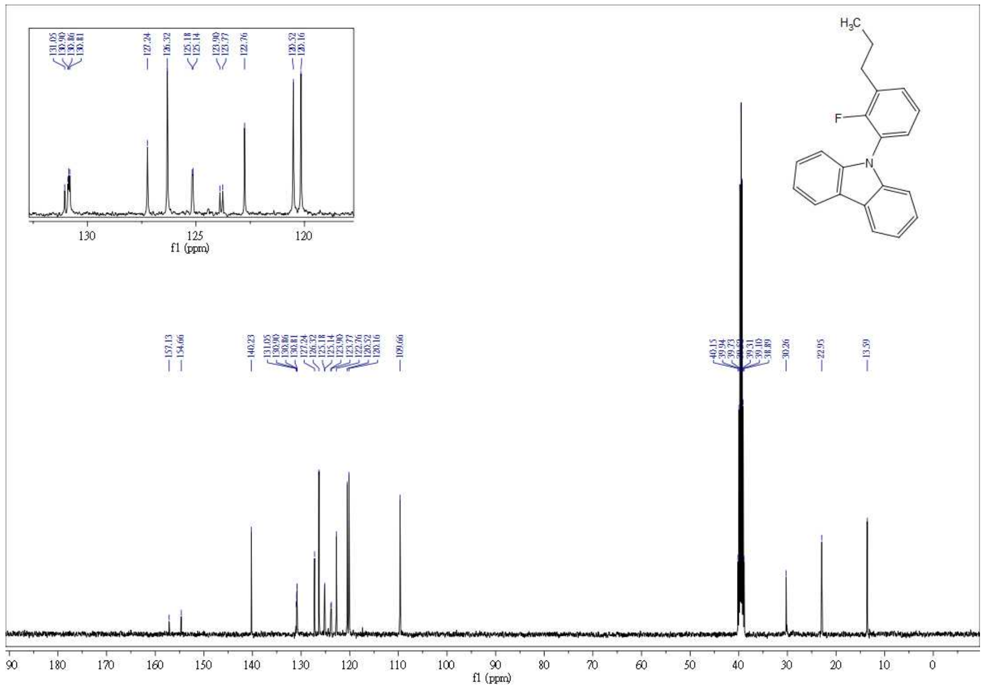

^{1}H-NMR spectrum of **5h** (400 MHz, $CDCl_3$)

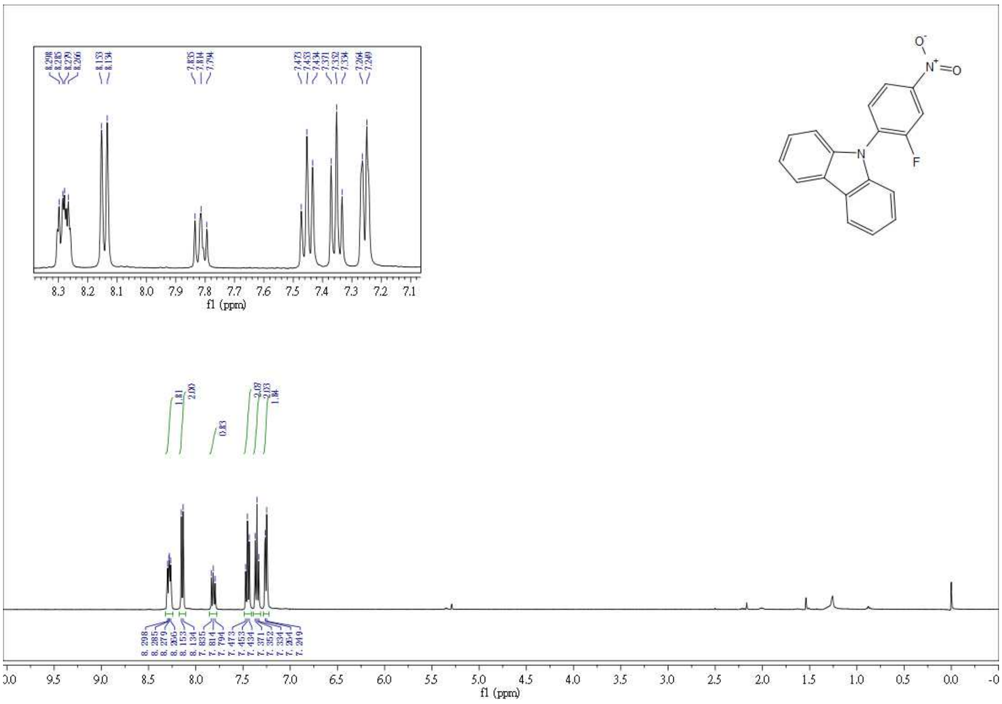

^{13}C-NMR spectrum of **5h** (100 MHz, $CDCl_3$)

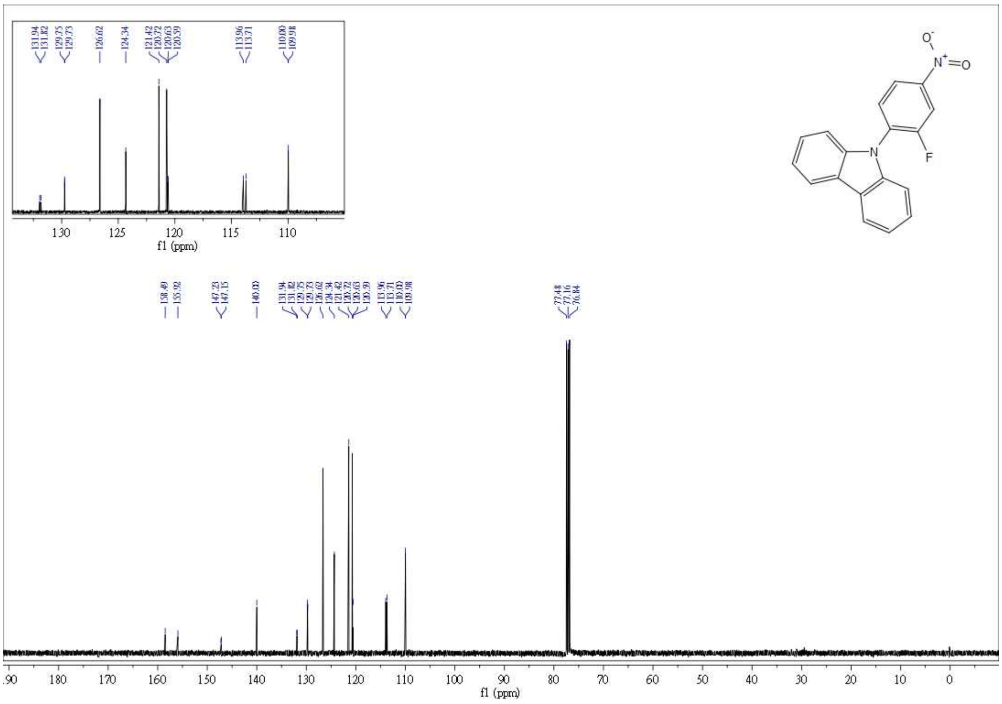

^{1}H-NMR spectrum of **5i** (400 MHz, $CDCl_3$)

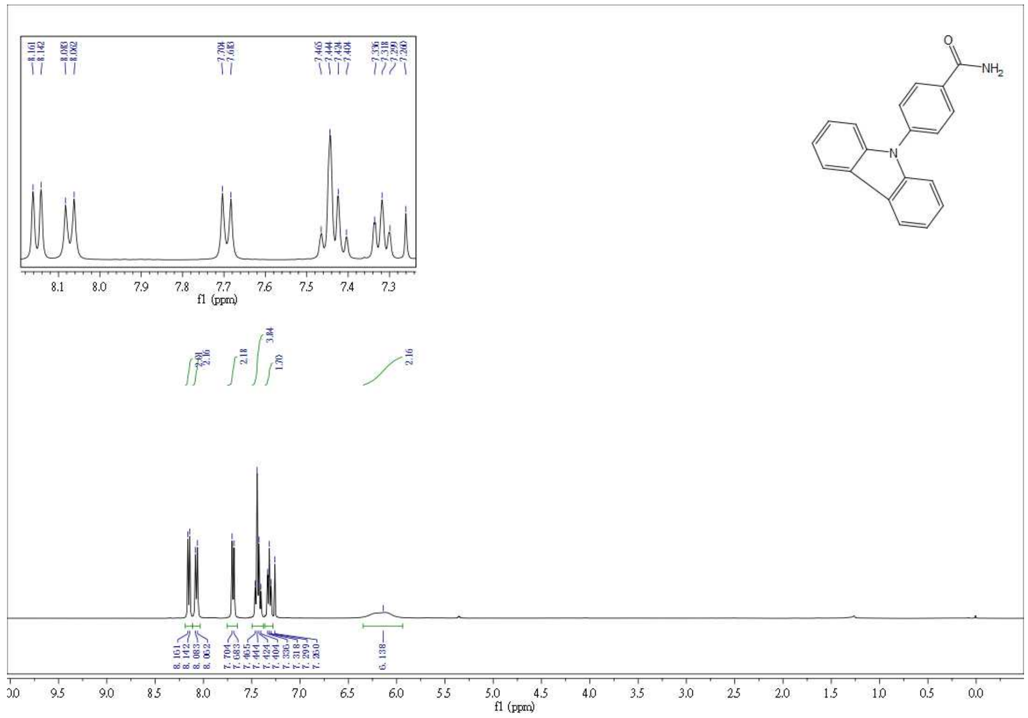

^{13}C-NMR spectrum of **5i** (100 MHz, $CDCl_3$)

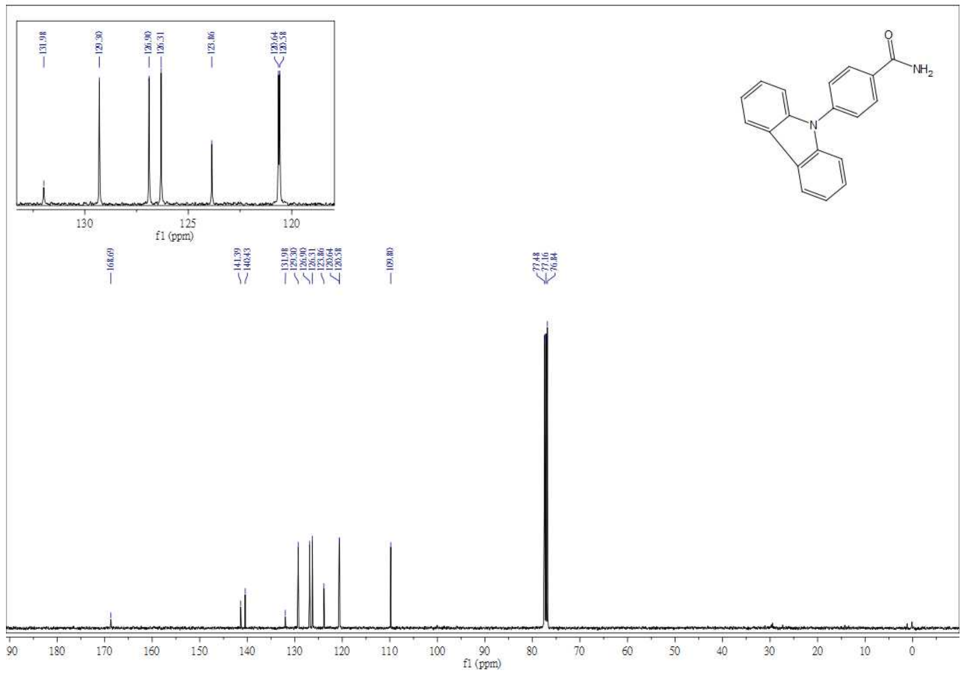

^{1}H-NMR spectrum of **5j** (400 MHz, DMSO-d_6)

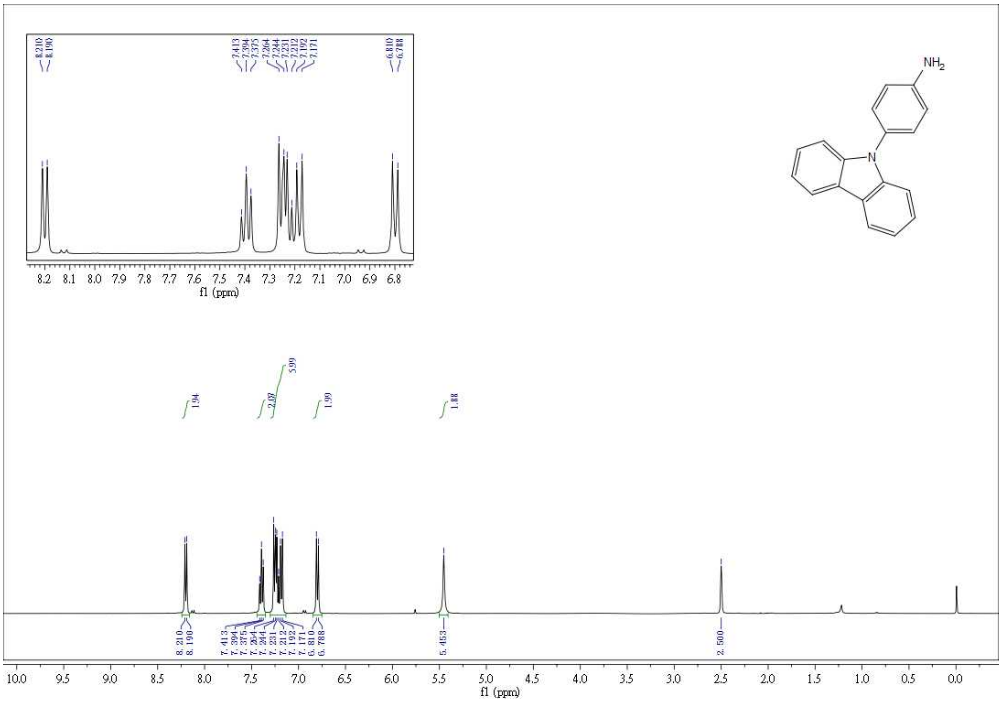

^{13}C-NMR spectrum of **5j** (100 MHz, DMSO-d_6)

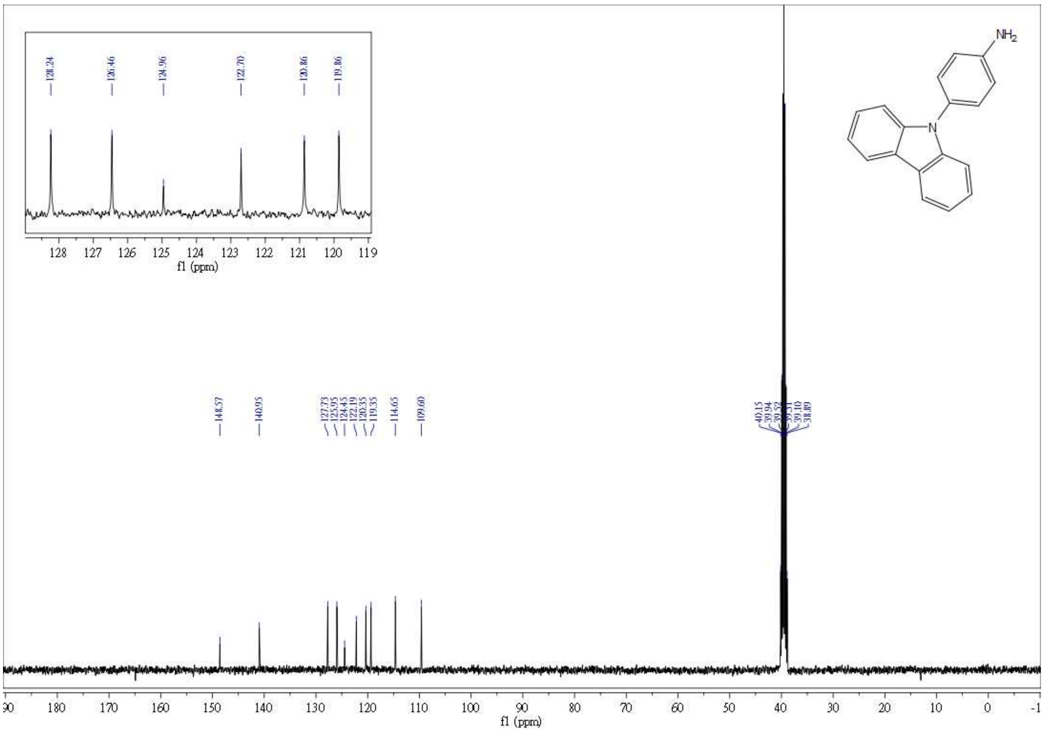

2. The data of 3aq crystal Structure

Crystal Structure of $C_{16}H_{13}FN_2O$

The room temperature [295(2)°K] single-crystal X-ray experiments were performed on a SuperNova diffractometer with Cu K_α radiation. Unit cell was obtained and refined by 3049 reflections with 5.1° < θ < 73.8°. No decay was observed in data collection. Raw intensities were corrected for Lorentz and polarization effects, and for absorption by empirical method. Direct phase determination yielded the positions of all non-hydrogen atoms. All non-hydrogen atoms were subjected to anisotropic refinement. The hydrogen atoms were generated geometrically with C-H bonds of 0.93-0.96 Å according to criteria described in the SHELXTL manual (Bruker, 1997). They were included in the refinement with $U_{iso}(H) = 1.2U_{eq}$ of their parent atoms. There were two molecules with the identical formula, $C_{16}H_{13}FN_2O$, in the independent area. However, the isoindolyl in one of them was diorientational disordered. The occupancies of the different orientational isoindolyl groups were refined to give 0.642(3) and 0.358(3), respectively. The final full-matric least-square refinement on F^2 converged with $R1 = 0.0873$ and $wR2 = 0.1451$ for 4361 observed reflections [$I \geq 2\sigma(I)$]. The final difference electron density map shows no features. Details of crystal parameters, data collection and structure refinement are given in Table 1.

Data collection was controlled by CrysAlis^{Pro} (Rigaku, 2016). Computations were performed using the SHELXTL NT ver. 5.10 program package (Bruker, 1997) on an IBM PC 586 computer. Analytic expressions of atomic scattering factors were employed, and anomalous dispersion corrections were incorporated (*International Tables for X-ray Crystallography*, 1989). Crystal drawings were produced with XP (Bruker, 1997).

References

Bruker. (1997) SHELXTL. Structure Determination Programs, Version 5.10, Bruker AXS Inc.,6300 Enterprise Lane, Madison, WI 53719-1173, USA.

International Tables for X-ray Crystallography: (1989) Vol. C (Kluwer Academic Publishers, Dordrecht) Tables 4.2.6.8 and 6.1.1.4.

Rigaku. (2016) CrysAlis^{Pro}, Data Collection and Process Software for Rigaku Oxford Diffraction X-ray Diffractometer, Version 5.4, February, 2016. Rigaku Corporation, 9009, New Trails Drive, The Woodlands, TX77381, USA.

Table 1. Details of Data Collection, Processing and Structure Refinement

Sample code	**20171016**
Molecular formula	$C_{16}H_{13}FN_2O$
Molecular weight	268.28
Color and habit	colorless block
Crystal size	0.2 × 0.25 × 0.6 mm
Crystal system	monoclinic
Space group	$P2_1/c$ (No. 14)
Unit cell parameters	a = 9.3959(2) Å α = 90.00°
	b = 21.1720(5) Å β = 100.676(3))°
	c = 14.3290(4) Å γ = 90.00°
	V = 2801.13(12) $Å^3$ Z = 8 $F(000)$ = 1120
Density (calcd)	1.272 g/cm^3
Diffractometer	SuperNova, Dual, Cu at zero, AtlasS2
Radiation	Cu K_α, λ = 1.54178 Å
Temperature	275(2)°K
Scan type	ω-scan
Data collection range	$-11 < h < 10$, $-24 < k < 25$, $-17 < l < 15$; $\theta_{max} = 74.4°$
Reflections measured	Total: 11381 Unique (n): 5578 Observed [I ≥ 2σ(I)]: 4361
Absorption coefficient	0.741 mm^{-1}
Minimum and maximum transmission	0.751, 1.000
No. of variables, p	399
Weighting scheme	$w = \frac{1}{\sigma^2(F_o^2) + (0.01P)^2 + 4.0P}$ $P = (F_o^2 + 2F_c^2)/3$
$R1 = \frac{\Sigma\|\|F_o\|-\|F_c\|\|}{\Sigma\|F_o\|}$ (for all reflections)	0.1023 0.0873 (for observed data)
$wR2 = \sqrt{\frac{\Sigma[w(F_o^2 - F_c^2)^2]}{\Sigma w(F_o^2)^2}}$ (for all reflections)	0.1513 0.1451 (for observed data)
Goof = S = $\sqrt{\frac{\Sigma[w(F_o^2 - F_c^2)^2]}{n - p}}$	1.028
Largest and mean Δ/σ	0.000, 0.000
Residual extrema in final difference map	-0.307 to 0.666 e $Å^{-3}$

Table 2. Atomic coordinates and equivalent isotropic temperature factors* (Å^2)

Atoms	*x*	*y*	*z*	$U_{eq.}$	*Occupancy*
F(1)	0.2409(3)	0.50672(12)	0.33687(16)	0.0968(8)	1.00
O(1)	0.3466(3)	0.49944(15)	0.1250(2)	0.0962(10)	1.00
N(1)	0.1612(4)	0.36123(14)	0.0806(2)	0.0837(10)	1.00
N(2)	0.1122(4)	0.51982(17)	0.1056(3)	0.0803(10)	1.00
C(1)	0.2876(6)	0.36219(18)	0.0322(3)	0.0947(14)	1.00
C(2)	0.2427(8)	0.3442(2)	-0.0578(4)	0.1191(16)	1.00
C(3)	0.0912(7)	0.33388(19)	-0.0704(3)	0.0982(14)	1.00
C(4)	-0.0114(10)	0.3149(3)	-0.1469(5)	0.163(3)	1.00
C(5)	-0.1559(10)	0.3086(4)	-0.1386(5)	0.174(3)	1.00
C(6)	-0.1854(9)	0.3175(4)	-0.0491(6)	0.178(3)	1.00
C(7)	-0.0858(6)	0.3374(2)	0.0316(4)	0.1028(15)	1.00
C(8)	0.0472(7)	0.3429(2)	0.0182(3)	0.0940(13)	1.00
C(9)	0.3329(7)	0.3374(2)	-0.1310(3)	0.142(3)	1.00
C(10)	0.1674(4)	0.37588(16)	0.1780(2)	0.0666(9)	1.00
C(11)	0.2030(3)	0.43599(15)	0.2095(2)	0.0506(7)	1.00
C(12)	0.2085(4)	0.44746(17)	0.3045(2)	0.0618(8)	1.00
C(13)	0.1803(5)	0.4031(2)	0.3671(2)	0.0806(11)	1.00
C(14)	0.1436(6)	0.3436(2)	0.3342(3)	0.0980(15)	1.00
C(15)	0.1387(6)	0.32945(19)	0.2398(3)	0.0995(16)	1.00
C(16)	0.2268(3)	0.48811(15)	0.1433(2)	0.0547(7)	1.00
F(2)	0.6546(4)	0.34613(12)	0.1729(2)	0.1208(10)	1.00
O(2)	0.8696(3)	0.46961(15)	0.1732(2)	0.0940(9)	1.00
N(4)	0.6438(4)	0.4947(2)	0.1080(3)	0.0854(11)	1.00
N(3)	0.7399(5)	0.5353(2)	0.3518(4)	0.0621(11)	0.642(3)
C(17)	0.8880(7)	0.5491(3)	0.3764(5)	0.0699(17)	0.642(3)
C(18)	0.9088(6)	0.6111(3)	0.4002(4)	0.0652(12)	0.642(3)
C(19)	0.7684(7)	0.6388(3)	0.3918(5)	0.0552(8)	0.642(3)
C(20)	0.7157(7)	0.6982(3)	0.4061(4)	0.0701(13)	0.642(3)
C(21)	0.5709(8)	0.7102(3)	0.3895(5)	0.0735(17)	0.642(3)
C(22)	0.4717(7)	0.6618(4)	0.3589(5)	0.0798(15)	0.642(3)
C(23)	0.5199(10)	0.5999(4)	0.3455(6)	0.0680(18)	0.642(3)
C(24)	0.6647(6)	0.5897(3)	0.3623(3)	0.0559(10)	0.642(3)
C(25)	1.0500(8)	0.6440(3)	0.4298(6)	0.091(2)	0.642(3)
N(3')	0.6615(10)	0.5429(5)	0.3382(8)	0.0621(11)	0.358(3)
C(17')	0.548(2)	0.5850(8)	0.3381(14)	0.0699(17)	0.358(3)
C(18')	0.5995(11)	0.6421(5)	0.3658(7)	0.0652(12)	0.358(3)
C(19')	0.7516(7)	0.6376(4)	0.3835(6)	0.0552(8)	0.358(3)
C(20')	0.8595(9)	0.6814(3)	0.4161(6)	0.0701(13)	0.358(3)
C(21')	1.0041(8)	0.6632(3)	0.4322(6)	0.0735(17)	0.358(3)

(Table 2. continued)

Atoms	*x*	*y*	*z*	$U_{eq.}$	*Occupancy*
C(22')	1.0408(7)	0.6011(4)	0.4158(7)	0.0798(15)	0.358(3)
C(23')	0.9329(9)	0.5573(3)	0.3832(6)	0.0680(18)	0.358(3)
C(24')	0.7883(8)	0.5755(3)	0.3671(5)	0.0559(10)	0.358(3)
C(25')	0.5105(17)	0.6994(8)	0.3715(14)	0.091(2)	0.358(3)
C(26)	0.6694(4)	0.47409(16)	0.3371(2)	0.0650(9)	1.00
C(27)	0.6835(3)	0.44118(14)	0.2563(2)	0.0524(7)	1.00
C(28)	0.6411(4)	0.37944(17)	0.2517(3)	0.0738(10)	1.00
C(29)	0.5830(5)	0.3500(2)	0.3213(3)	0.0931(13)	1.00
C(30)	0.5688(5)	0.3836(2)	0.3998(3)	0.0867(12)	1.00
C(31)	0.6125(5)	0.44563(19)	0.4088(3)	0.0791(11)	1.00
C(32)	0.7415(3)	0.47042(16)	0.1752(2)	0.0580(8)	1.00

*$U_{eq.}$ defined as one third of the trace of the orthogonalized **U** tensor.

Table 3. Bond lengths (Å) and bond angles (°)

F(1)-C(12)	1.353(4)	C(17)-C(18)	1.361(8)
O(1)-C(16)	1.226(3)	C(18)-C(19)	1.429(8)
N(1)-C(8)	1.320(6)	C(18)-C(25)	1.489(8)
N(1)-C(10)	1.420(4)	C(19)-C(20)	1.381(8)
N(1)-C(1)	1.483(6)	C(19)-C(24)	1.433(8)
N(2)-C(16)	1.299(4)	C(20)-C(21)	1.361(9)
C(1)-C(2)	1.336(6)	C(21)-C(22)	1.399(9)
C(2)-C(3)	1.417(8)	C(22)-C(23)	1.411(10)
C(2)-C(9)	1.473(6)	C(23)-C(24)	1.354(11)
C(3)-C(4)	1.379(8)	N(3')-C(24')	1.373(11)
C(3)-C(8)	1.419(6)	N(3')-C(17')	1.389(18)
C(4)-C(5)	1.392(11)	N(3')-C(26)	1.458(11)
C(5)-C(6)	1.374(10)	C(17')-C(18')	1.34(2)
C(6)-C(7)	1.410(8)	C(18')-C(19')	1.408(12)
C(7)-C(8)	1.304(7)	C(18')-C(25')	1.48(2)
C(10)-C(11)	1.371(5)	C(19')-C(20')	1.3900
C(10)-C(15)	1.383(5)	C(19')-C(24')	1.3900
C(11)-C(12)	1.374(4)	C(20')-C(21')	1.3900
C(11)-C(16)	1.499(4)	C(21')-C(22')	1.3900
C(12)-C(13)	1.358(5)	C(22')-C(23')	1.3900
C(13)-C(14)	1.367(6)	C(23')-C(24')	1.3900
C(14)-C(15)	1.379(5)	C(26)-C(27)	1.379(4)
F(2)-C(28)	1.357(4)	C(26)-C(31)	1.381(5)
O(2)-C(32)	1.209(4)	C(27)-C(28)	1.365(5)
N(4)-C(32)	1.307(5)	C(27)-C(32)	1.505(4)
N(3)-C(24)	1.374(7)	C(28)-C(29)	1.372(5)
N(3)-C(17)	1.402(7)	C(29)-C(30)	1.359(6)
N(3)-C(26)	1.454(6)	C(30)-C(31)	1.375(6)
C(8)-N(1)-C(10)	127.6(4)	C(5)-C(6)-C(7)	126.0(8)
C(8)-N(1)-C(1)	107.9(4)	C(8)-C(7)-C(6)	114.5(6)
C(10)-N(1)-C(1)	124.5(4)	C(7)-C(8)-N(1)	127.3(5)
C(2)-C(1)-N(1)	108.1(5)	C(7)-C(8)-C(3)	124.5(6)
C(1)-C(2)-C(3)	107.3(5)	N(1)-C(8)-C(3)	108.1(5)
C(1)-C(2)-C(9)	126.5(7)	C(11)-C(10)-C(15)	120.9(3)
C(3)-C(2)-C(9)	126.3(6)	C(11)-C(10)-N(1)	119.3(3)
C(4)-C(3)-C(2)	133.1(6)	C(15)-C(10)-N(1)	119.8(3)
C(4)-C(3)-C(8)	118.3(7)	C(10)-C(11)-C(12)	116.9(3)
C(2)-C(3)-C(8)	108.6(4)	C(10)-C(11)-C(16)	122.0(3)
C(3)-C(4)-C(5)	120.7(7)	C(12)-C(11)-C(16)	121.0(3)
C(6)-C(5)-C(4)	115.8(7)	F(1)-C(12)-C(13)	118.3(3)

(Table 3. continued)

F(1)-C(12)-C(11)	117.9(3)	C(17')-C(18')-C(25')	125.5(13)
C(13)-C(12)-C(11)	123.8(3)	C(19')-C(18')-C(25')	127.5(10)
C(12)-C(13)-C(14)	118.5(3)	C(20')-C(19')-C(24')	120.0
C(13)-C(14)-C(15)	119.9(4)	C(20')-C(19')-C(18')	131.9(7)
C(14)-C(15)-C(10)	120.0(4)	C(24')-C(19')-C(18')	108.0(7)
O(1)-C(16)-N(2)	122.3(3)	C(21')-C(20')-C(19')	120.0
O(1)-C(16)-C(11)	122.0(3)	C(20')-C(21')-C(22')	120.0
N(2)-C(16)-C(11)	115.7(3)	C(23')-C(22')-C(21')	120.0
C(24)-N(3)-C(17)	107.8(5)	C(24')-C(23')-C(22')	120.0
C(24)-N(3)-C(26)	122.3(4)	N(3')-C(24')-C(23')	132.6(7)
C(17)-N(3)-C(26)	128.8(5)	N(3')-C(24')-C(19')	107.4(7)
C(18)-C(17)-N(3)	110.7(6)	C(23')-C(24')-C(19')	120.0
C(17)-C(18)-C(19)	106.7(5)	C(27)-C(26)-C(31)	121.0(3)
C(17)-C(18)-C(25)	127.0(6)	C(27)-C(26)-N(3)	117.4(3)
C(19)-C(18)-C(25)	126.3(6)	C(31)-C(26)-N(3)	120.7(4)
C(20)-C(19)-C(18)	135.5(6)	C(27)-C(26)-N(3')	121.8(5)
C(20)-C(19)-C(24)	117.5(6)	C(31)-C(26)-N(3')	113.6(5)
C(18)-C(19)-C(24)	107.1(5)	C(28)-C(27)-C(26)	116.9(3)
C(21)-C(20)-C(19)	121.2(6)	C(28)-C(27)-C(32)	120.1(3)
C(20)-C(21)-C(22)	120.3(7)	C(26)-C(27)-C(32)	123.1(3)
C(21)-C(22)-C(23)	120.7(7)	F(2)-C(28)-C(27)	117.8(3)
C(24)-C(23)-C(22)	117.4(9)	F(2)-C(28)-C(29)	118.6(4)
C(23)-C(24)-N(3)	129.3(6)	C(27)-C(28)-C(29)	123.5(4)
C(23)-C(24)-C(19)	122.9(6)	C(30)-C(29)-C(28)	118.5(4)
N(3)-C(24)-C(19)	107.8(5)	C(29)-C(30)-C(31)	120.3(4)
C(24')-N(3')-C(17')	107.5(11)	C(30)-C(31)-C(26)	119.8(4)
C(24')-N(3')-C(26)	117.6(8)	O(2)-C(32)-N(4)	123.7(3)
C(17')-N(3')-C(26)	133.0(12)	O(2)-C(32)-C(27)	121.1(3)
C(18')-C(17')-N(3')	110.2(17)	N(4)-C(32)-C(27)	115.1(3)
C(17')-C(18')-C(19')	107.0(12)		
Hydrogen bondings			
H(2A)···O(2)[#1]	2.10(4)	H(4B)···O(1)	2.03(5)
N(2)-H(2A)···O(2)[#1]	160(4)	N(4)-H(4B)···O(1)	169(5)

Symmetry transformation codes: #1(-1+*x*, *y*, *z*).

Table 4. Anisotropic thermal parameters* (Å^2)

Atoms	U_{11}	U_{22}	U_{33}	U_{23}	U_{13}	U_{12}
F(1)	0.1151(19)	0.1036(18)	0.0760(14)	-0.0285(13)	0.0292(13)	-0.0400(15)
O(1)	0.0509(14)	0.111(2)	0.135(3)	0.035(2)	0.0397(16)	-0.0013(14)
N(1)	0.142(3)	0.0608(18)	0.0462(16)	0.0018(14)	0.0110(19)	-0.0002(19)
N(2)	0.0559(18)	0.084(2)	0.105(3)	0.043(2)	0.0269(18)	0.0106(16)
C(1)	0.178(4)	0.066(2)	0.0497(19)	0.0074(17)	0.046(2)	0.017(3)
C(2)	0.208(4)	0.086(3)	0.073(3)	0.010(2)	0.049(3)	0.025(4)
C(3)	0.194(4)	0.053(2)	0.0409(17)	0.0024(15)	0.003(2)	0.031(3)
C(4)	0.264(6)	0.109(5)	0.089(3)	-0.012(3)	-0.038(4)	0.038(6)
C(5)	0.229(6)	0.126(5)	0.122(4)	-0.018(4)	-0.082(6)	0.013(6)
C(6)	0.180(6)	0.153(7)	0.172(6)	0.001(6)	-0.040(5)	0.019(6)
C(7)	0.118(4)	0.087(3)	0.095(3)	-0.003(3)	0.001(3)	0.024(3)
C(8)	0.143(4)	0.059(2)	0.077(2)	0.012(2)	0.010(3)	0.023(3)
C(9)	0.271(8)	0.095(3)	0.089(3)	0.005(3)	0.109(4)	0.026(4)
C(10)	0.100(3)	0.058(2)	0.0428(16)	0.0068(14)	0.0175(17)	0.0102(18)
C(11)	0.0430(15)	0.0620(18)	0.0479(16)	0.0054(13)	0.0113(12)	0.0074(13)
C(12)	0.0580(19)	0.074(2)	0.0535(18)	-0.0066(16)	0.0118(15)	-0.0041(16)
C(13)	0.102(3)	0.099(3)	0.0432(18)	0.0026(19)	0.0198(19)	0.004(2)
C(14)	0.165(5)	0.080(3)	0.056(2)	0.018(2)	0.039(3)	0.005(3)
C(15)	0.186(5)	0.059(2)	0.059(2)	0.0098(18)	0.036(3)	0.000(3)
C(16)	0.0447(16)	0.0602(18)	0.0617(18)	0.0017(15)	0.0165(14)	-0.0012(14)
F(2)	0.184(3)	0.0766(16)	0.115(2)	-0.0366(15)	0.064(2)	-0.0363(18)
O(2)	0.0458(13)	0.124(2)	0.118(2)	0.0250(19)	0.0315(14)	0.0052(14)
N(4)	0.0525(18)	0.132(3)	0.076(2)	0.035(2)	0.0236(17)	0.0000(19)
N(3)	0.059(3)	0.057(2)	0.074(3)	-0.0128(19)	0.020(3)	-0.006(3)
C(17)	0.058(4)	0.072(3)	0.082(4)	-0.015(3)	0.018(3)	-0.007(3)
C(18)	0.069(3)	0.068(3)	0.059(3)	-0.003(2)	0.015(2)	-0.009(2)
C(19)	0.072(2)	0.0562(18)	0.0369(17)	-0.0002(14)	0.0101(16)	-0.0094(17)
C(20)	0.100(4)	0.058(3)	0.052(2)	0.002(2)	0.012(3)	-0.009(3)
C(21)	0.091(5)	0.058(3)	0.070(3)	0.007(3)	0.011(3)	0.003(3)
C(22)	0.071(4)	0.091(4)	0.076(4)	0.011(3)	0.010(3)	0.007(3)
C(23)	0.059(4)	0.080(5)	0.066(4)	0.004(3)	0.013(3)	0.000(3)
C(24)	0.064(3)	0.060(3)	0.045(2)	-0.0003(19)	0.015(2)	-0.003(2)
C(25)	0.075(4)	0.077(4)	0.120(6)	-0.013(4)	0.010(4)	-0.011(3)
N(3')	0.059(3)	0.057(2)	0.074(3)	-0.0128(19)	0.020(3)	-0.006(3)
C(17')	0.058(4)	0.072(3)	0.082(4)	-0.015(3)	0.018(3)	-0.007(3)
C(18')	0.069(3)	0.068(3)	0.059(3)	-0.003(2)	0.015(2)	-0.009(2)
C(19')	0.072(2)	0.0562(18)	0.0369(17)	-0.0002(14)	0.0101(16)	-0.0094(17)
C(20')	0.100(4)	0.058(3)	0.052(2)	0.002(2)	0.012(3)	-0.009(3)
C(21')	0.091(5)	0.058(3)	0.070(3)	0.007(3)	0.011(3)	0.003(3)

(Table 4. continued)

Atoms	U_{11}	U_{22}	U_{33}	U_{23}	U_{13}	U_{12}
C(22')	0.071(4)	0.091(4)	0.076(4)	0.011(3)	0.010(3)	0.007(3)
C(23')	0.059(4)	0.080(5)	0.066(4)	0.004(3)	0.013(3)	0.000(3)
C(24')	0.064(3)	0.060(3)	0.045(2)	-0.0003(19)	0.015(2)	-0.003(2)
C(25')	0.075(4)	0.077(4)	0.120(6)	-0.013(4)	0.010(4)	-0.011(3)
C(26)	0.078(2)	0.0561(19)	0.063(2)	-0.0024(16)	0.0197(17)	-0.0053(17)
C(27)	0.0440(15)	0.0539(17)	0.0600(18)	-0.0008(14)	0.0117(13)	-0.0009(13)
C(28)	0.089(3)	0.060(2)	0.075(2)	-0.0099(18)	0.022(2)	-0.0069(19)
C(29)	0.120(4)	0.061(2)	0.102(3)	0.010(2)	0.029(3)	-0.021(2)
C(30)	0.100(3)	0.085(3)	0.079(3)	0.022(2)	0.027(2)	-0.010(2)
C(31)	0.099(3)	0.079(3)	0.064(2)	0.0029(19)	0.026(2)	0.002(2)
C(32)	0.0479(17)	0.0617(19)	0.068(2)	-0.0057(16)	0.0200(15)	-0.0045(14)

*The exponent takes the form: $-2\pi^2\Sigma\Sigma U_{ij}h_ih_j\mathbf{a}_i^*\mathbf{a}_j^*$

Table 5. Coordinates and isotropic temperature factors* (Å^2) for H atoms

Atoms	*x*	*y*	*z*	$U_{eq.}$	*Occupancy*
H(2A)	0.038(5)	0.515(2)	0.121(3)	0.093(15)	1.00
H(2B)	0.117(4)	0.555(2)	0.063(3)	0.101(14)	1.00
H(1)	0.3818	0.3734	0.0597	0.114	1.00
H(4)	0.0163	0.3062	-0.2046	0.196	1.00
H(5)	-0.2280	0.2989	-0.1903	0.208	1.00
H(6)	-0.2797	0.3096	-0.0409	0.213	1.00
H(7)	-0.1127	0.3459	0.0896	0.123	1.00
H(9A)	0.2885	0.3596	-0.1874	0.213	1.00
H(9B)	0.3418	0.2935	-0.1456	0.213	1.00
H(9C)	0.4273	0.3547	-0.1079	0.213	1.00
H(13)	0.1858	0.4130	0.4309	0.097	1.00
H(14)	0.1220	0.3126	0.3755	0.118	1.00
H(15)	0.1161	0.2887	0.2176	0.119	1.00
H(4A)	0.663(4)	0.516(2)	0.065(3)	0.092(15)	1.00
H(4B)	0.557(5)	0.491(2)	0.111(3)	0.110(17)	1.00
H(17A)	0.9619	0.5199	0.3764	0.084	0.642(3)
H(20A)	0.7803	0.7307	0.4274	0.084	0.642(3)
H(21A)	0.5376	0.7507	0.3985	0.088	0.642(3)
H(22A)	0.3730	0.6705	0.3474	0.096	0.642(3)
H(23A)	0.4548	0.5674	0.3258	0.082	0.642(3)
H(25A)	1.1263	0.6183	0.4139	0.137	0.642(3)
H(25B)	1.0672	0.6511	0.4971	0.137	0.642(3)
H(25C)	1.0476	0.6838	0.3974	0.137	0.642(3)
H(17B)	0.4503	0.5748	0.3211	0.084	0.358(3)
H(20B)	0.8350	0.7230	0.4271	0.084	0.358(3)
H(21B)	1.0763	0.6925	0.4540	0.088	0.358(3)
H(22B)	1.1376	0.5889	0.4266	0.096	0.358(3)
H(23B)	0.9574	0.5157	0.3722	0.082	0.358(3)
H(25D)	0.4097	0.6883	0.3580	0.137	0.358(3)
H(25E)	0.5299	0.7300	0.3260	0.137	0.358(3)
H(25F)	0.5342	0.7170	0.4342	0.137	0.358(3)
H(29)	0.5539	0.3080	0.3148	0.112	1.00
H(30)	0.5293	0.3646	0.4477	0.104	1.00
H(31)	0.6039	0.4683	0.4631	0.095	1.00

*The exponent takes the form: $-8\pi^2 U \sin^2\theta/\lambda^2$

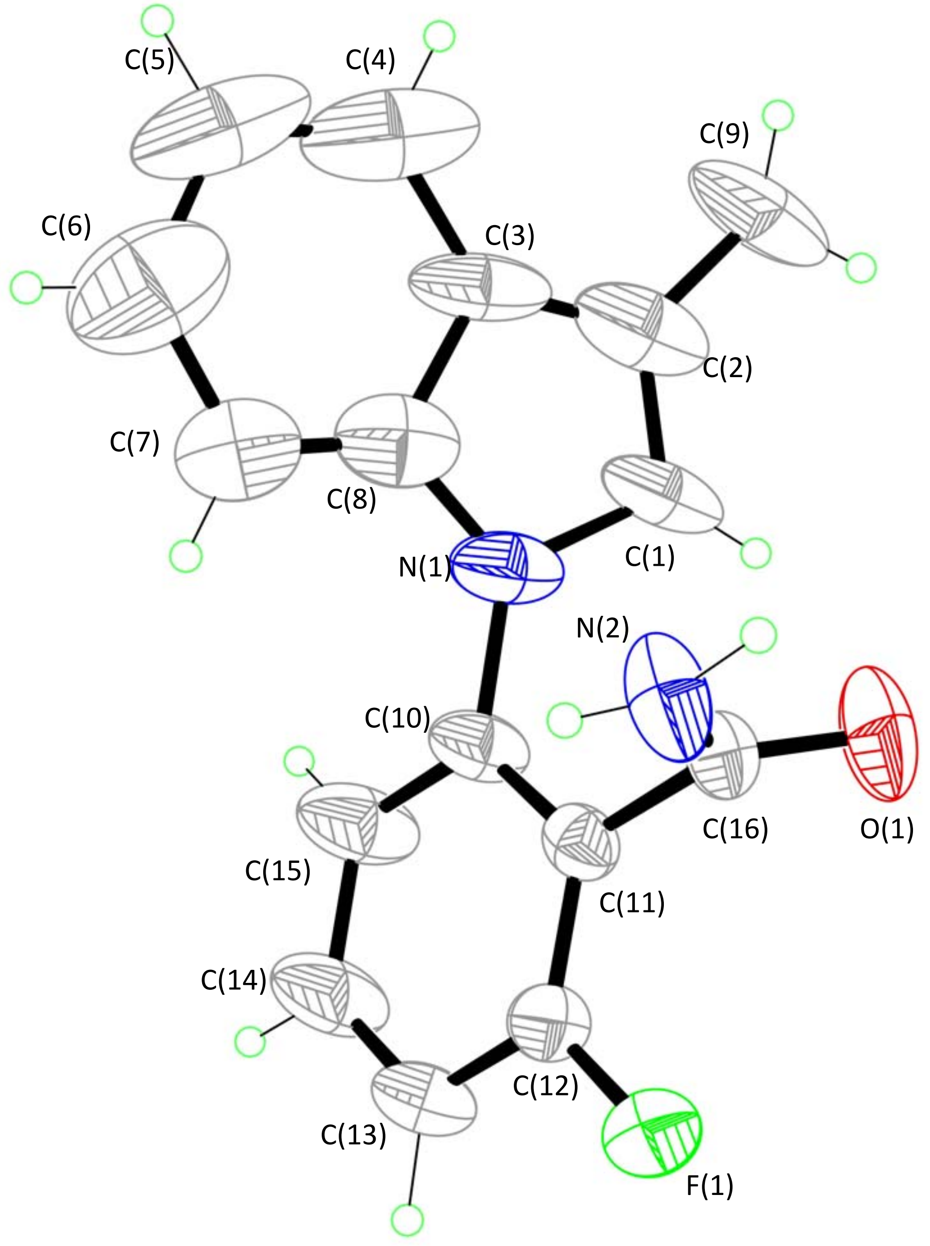

Molecule **I**

ORTEP drawing of $C_{16}H_{13}FN_2O$ with 35% probability ellipsoids, showing the atomic numbering scheme.

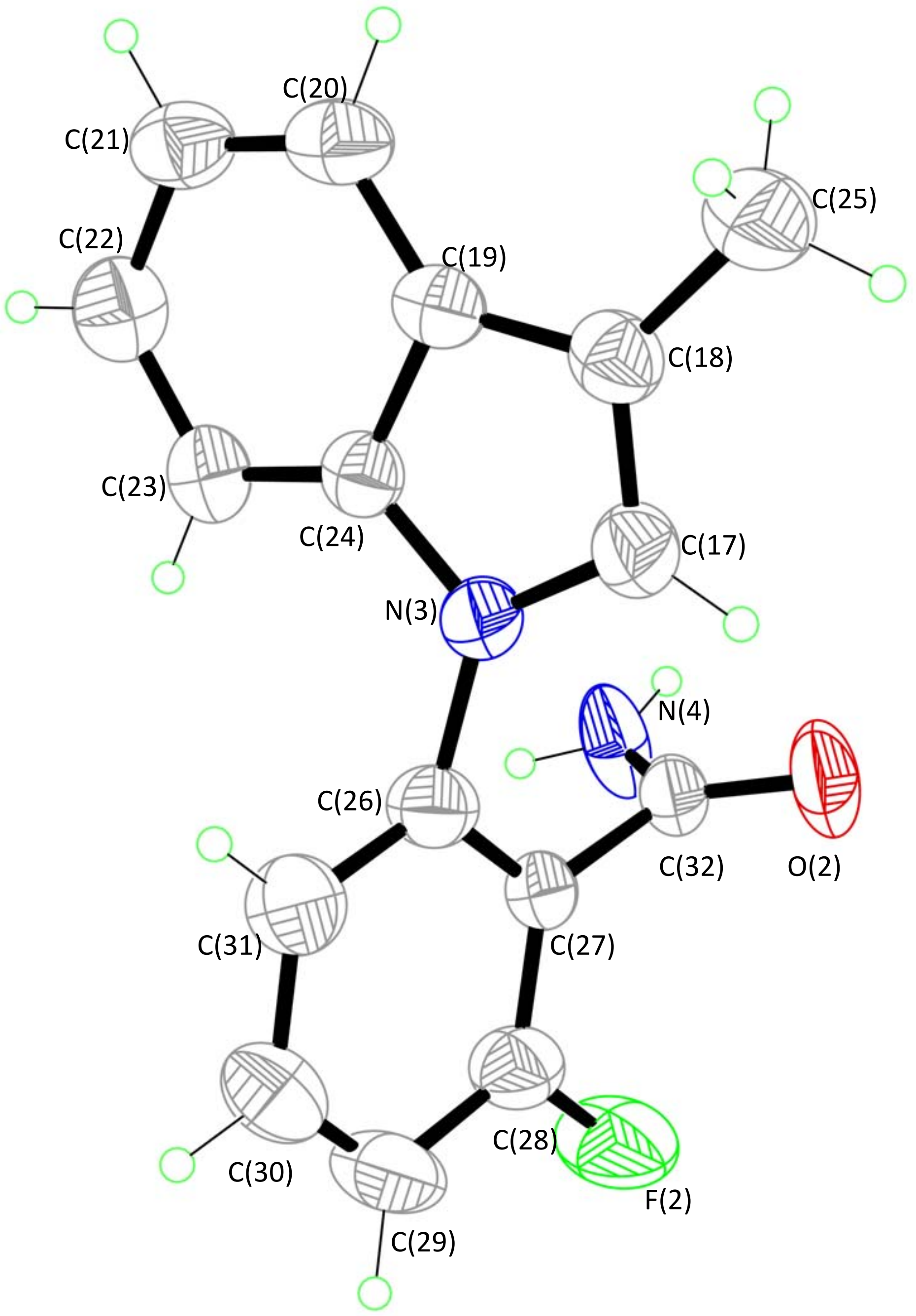

Molecule **II**

ORTEP drawing of $C_{16}H_{13}FN_2O$ with 35% probability ellipsoids, showing the atomic numbering scheme and an orientation of isoindolyl presented by N(3), C(17)-C(25).

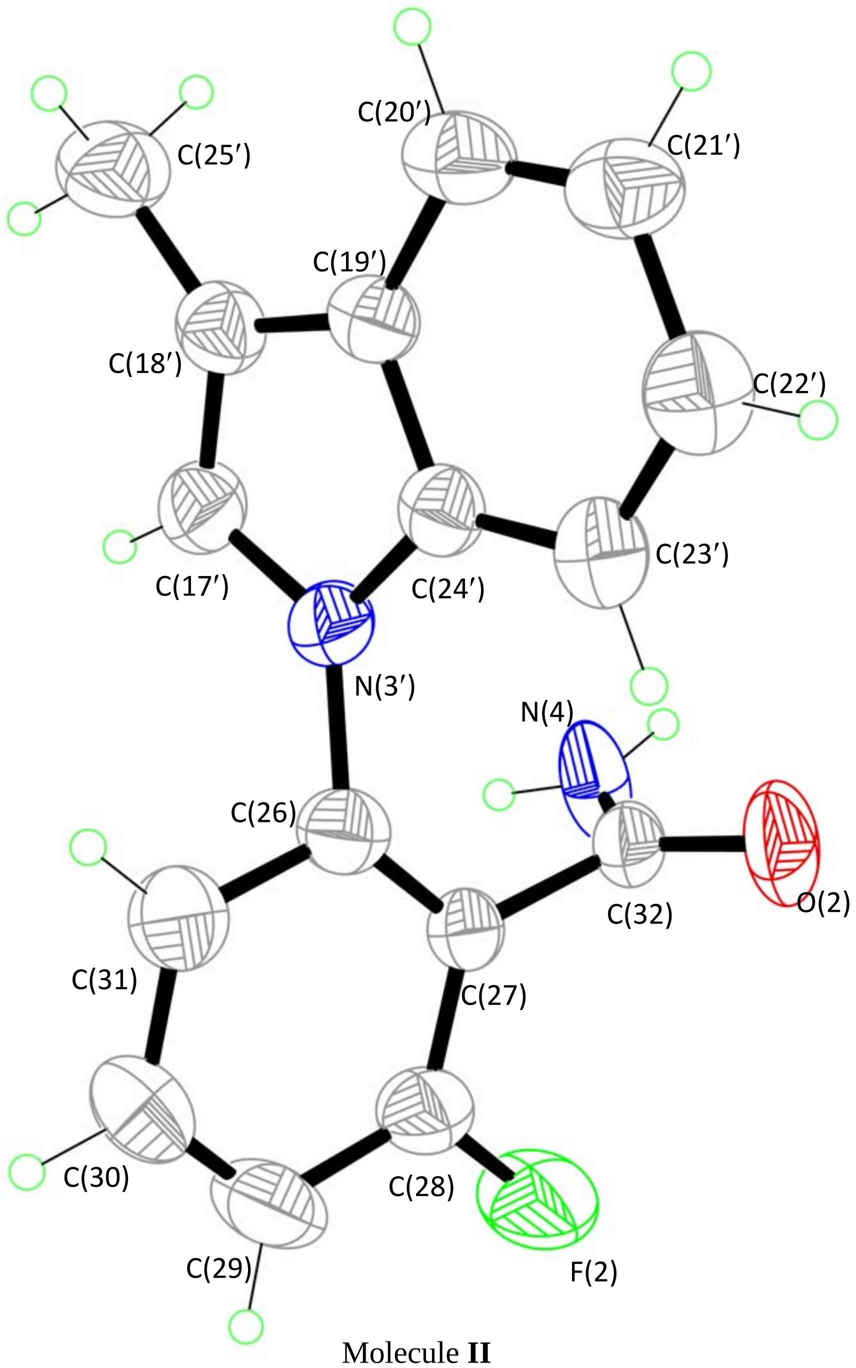

Molecule **II**

ORTEP drawing of $C_{16}H_{13}FN_2O$ with 35% probability ellipsoids, showing the atomic numbering scheme and an orientation of isoindolyl presented by N(3′), C(17′)-C(25′).

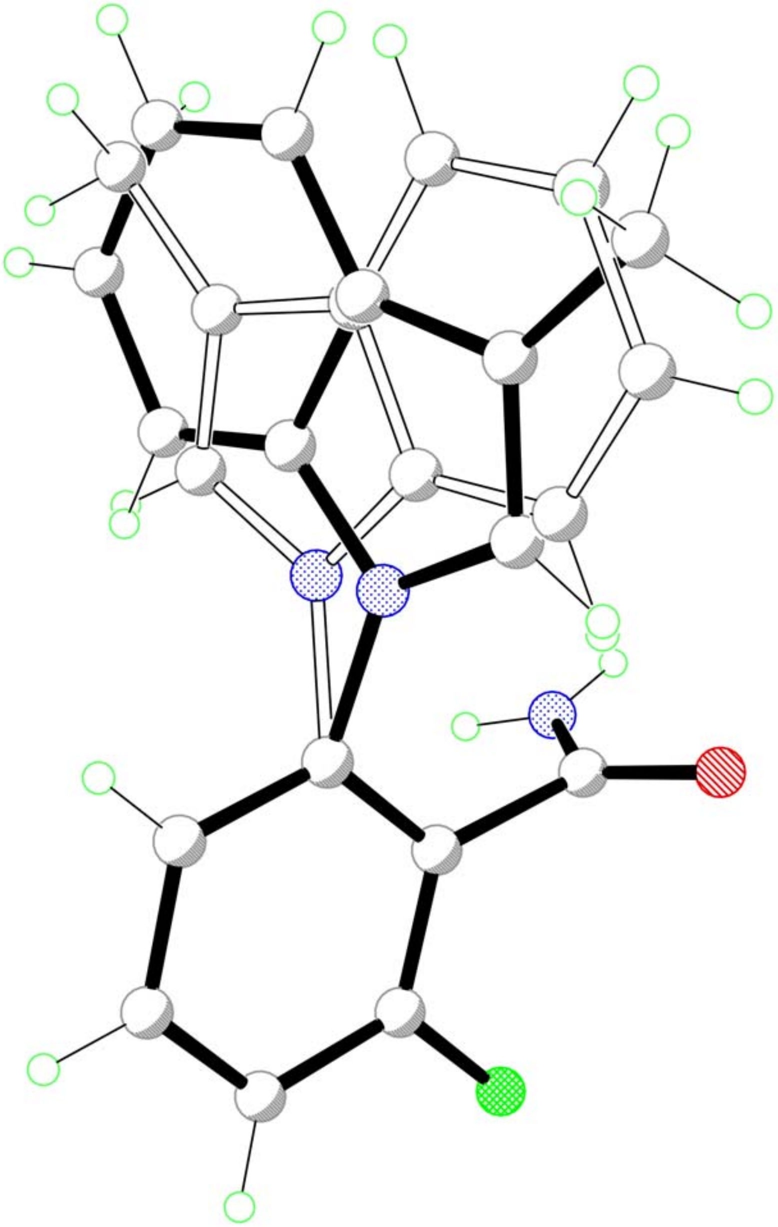

The different orientations of the isoindolyl groups in Molecule **II,** with solid bonds for an orientation and open bonds for another.

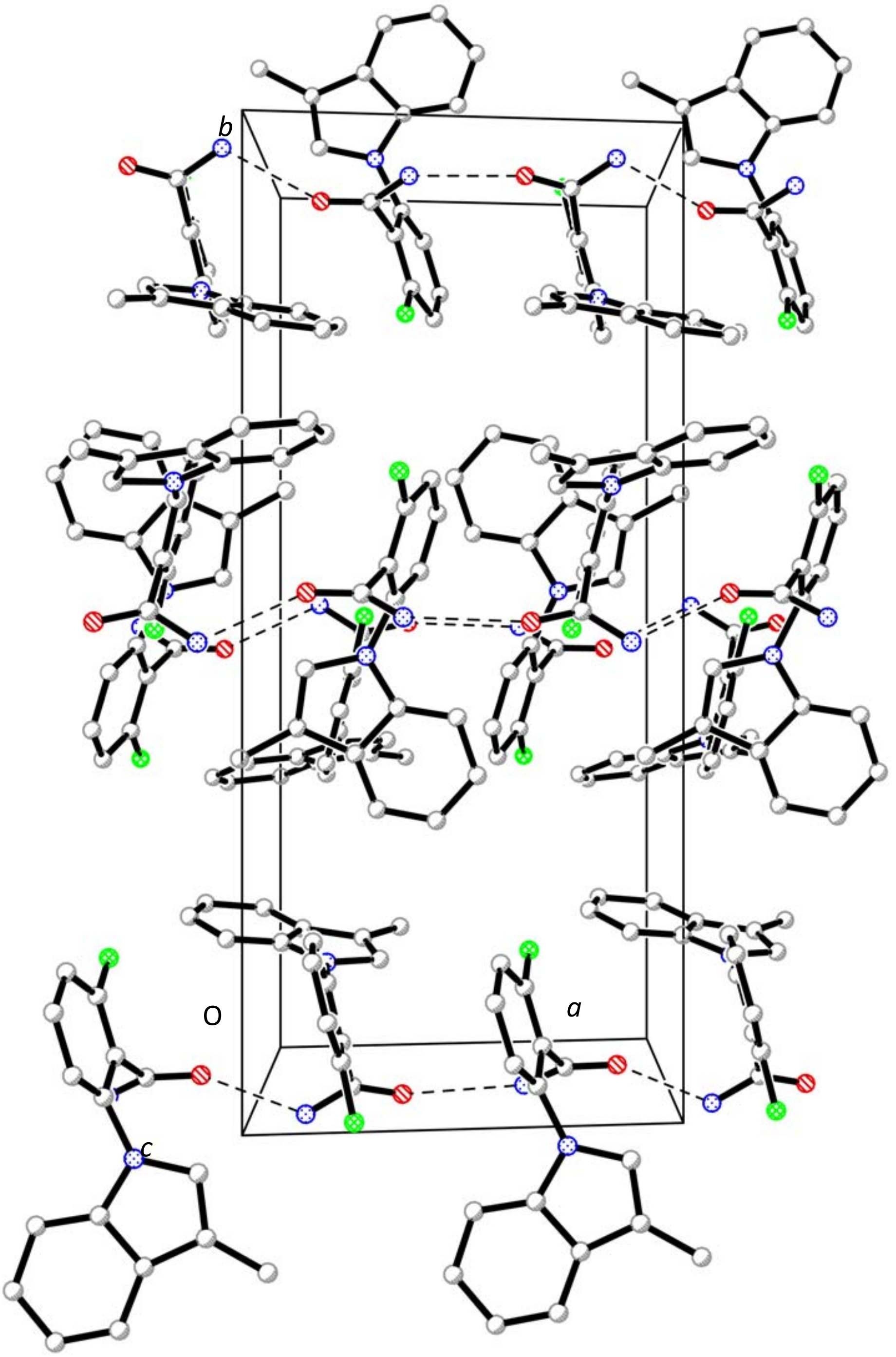

A packing view along the *c* direction
